# Tip-dated phylogeny of whirligig beetles reveals ancient lineage surviving on Madagascar

**DOI:** 10.1038/s41598-017-08403-1

**Published:** 2017-08-22

**Authors:** Grey T. Gustafson, Alexander A. Prokin, Rasa Bukontaite, Johannes Bergsten, Kelly B. Miller

**Affiliations:** 1 Department of Ecology and Evolutionary Biology, University of Kansas, Lawrence, KS 66046 USA; 2 Papanin Institute for Biology of Inland Waters, Russian Academy of Sciences, Borok, Nekouzsky District, Yaroslavl Oblast 152742 Russia; 3 Department of Zoology, Swedish Museum of Natural History, Box 50007, SE-104 05 Stockholm, Sweden; 4 Department of Biology and Museum of Southwestern Biology, University of New Mexico, Albuquerque, NM 87131 USA

## Abstract

The temporal origin of Madagascar’s extraordinary endemic diversity is debated. A preference for Cenozoic dispersal origins has replaced the classical view of Mesozoic vicariance in the wake of molecular dating. However, evidence of ancient origins is mounting from arthropod groups. Using phylogenetic ‘tip-dating’ analysis with fossils, we show that a whirligig beetle species, *Heterogyrus milloti*, inhabiting forest streams in southeastern Madagascar is the last survivor of a once dominant and widespread Mesozoic group. With a Late Triassic to Early Jurassic origin (226–187 Ma) it is the hitherto oldest dated endemic lineage of animal or plant on Madagascar. Island biotas’ sensitivity to extinction is well known, but islands can also provide refuge from continental extinction. *Heterogyrus milloti* is an irreplaceable link to the freshwater biota of the Mesozoic and serves as a reminder of what may be lost without critical conservation efforts on Madagascar.

## Introduction

Madagascar is well known as one of the world’s most important biodiversity hotspots with exceptional levels of endemism among animals and plants, as well as severe habitat degradation threatening their existence^1^. The origin of this unique flora and fauna has long fascinated biologist given its central position in Gondwana and extensive isolation following its separation from India^2, 3^. Few studies, however, have revealed taxa with truly Gondwanan vicariant relationships or ancient origins^2^. Instead the most iconic of Madagascar’s fauna, the lemurs, tenrecs, and unique carnivorans, appear to have rafted to the island^4^ within the past sixty million years^5^. Dated molecular phylogenies further revealed most of the extant endemic vertebrate fauna have colonized via oversea dispersal during the Cenozoic^6, 7^. A few notable exceptions include Malagasy iguanid lizards, podocnemidid turtles, mantellid frogs, and cichlid fish, which show Mesozoic origins^6–8^. While comparatively few phylogenetic studies have been carried out on the invertebrates of Madagascar^2^, some of these groups represent the oldest endemic lineages currently known^9–11^.

The aquatic insects of Madagascar are similarly known for their endemicity^12^. Among these, whirligig beetles (family Gyrinidae) show a typical pattern^12^ with 96% of the Malagasy species being endemic. Whirligig beetles are a family of carnivorous aquatic beetles with c. 1000 species, relatively well known as model organisms for life on the water’s surface^13^. Contrary to semi-aquatic insects that walk on the surface, the whirligig body is partly submerged, much like a boat, inspiring research in biomimetic engineering and robotics^14^. The propulsive efficiency of the swimming legs is believed to be the highest measured for a thrust-generating apparatus within the animal kingdom^15^. This adaptation to the surface of water is a unique specialization within beetles, but may also have represented a stepping-stone towards fully submerged aquatic life in related lineages^16^.

Among beetles, morphological data have supported a sister-group relationship of Gyrinidae to all other families in the suborder Adephaga, aquatic and terrestrial^17^. Molecular data instead largely support a monophyletic origin of all aquatic Adephaga, with whirligig beetles sister to other aquatic families, in line with the stepping-stone hypothesis^16, 18, 19^. As adephagan beetles have a fossil record dating back to the Permian^20^, the basal position of whirligig beetles along with a controversial Permian larva and Triassic adult^17^, feeds the suspicion of an ancient group. While the dated analysis of beetles from the ATOL initiative (Assembling the Beetle Tree of Life) found crown-Gyrinidae was not older than early Cretaceous^19^, this is at odds with the fossil record^20^.

Crown-Gyrinidae includes the ‘primitive’ species *Spanglerogyrus albiventris*, discovered in Alabama as late as 1979^21^. *Spanglerogyrus* displays numerous ancestral characters such as narrowly divided eyes, and unbroadened (non-“paddle-like”) mid- and hind-leg segments^21^. It has been regarded as a lone sister lineage to modern whirligig beetles, but a recent phylogenetic analysis of Gyrinidae revealed a Malagasy species, *Heterogyrus milloti*, represents a second monotypic lineage basal in the phylogeny^22^, but without strong support.

To test the phylogenetic position of the Malagasy *H*. *milloti* and construct a time calibrated tree, we analyzed the morphology of all well-preserved Mesozoic and early Cenozoic fossil gyrinid species known to date (Table 1), scoring morphological characters of extinct species along with >10% of extant whirligig species in a single datamatrix (Table S1). We analyzed this together with an extended molecular dataset from ref. 22, using total evidence dating (TED), or “tip dating”, in a Bayesian framework^23, 24^. We combined TED with the fossilized birth-death process model as a tree- (and relative node age) prior^24, 25^. This landmark model integrates fossil and extant taxa in the same diversification process using parameters for speciation rate, extinction rate, and fossilization rate, while serving as a tree prior^25^.

**Table 1:** Inferred subfamily of known fossil Gyrinidae.

Subfamily	Genus	Species	Authority	Age	Epoch	Fossil deposit
Gyrininae	*Protogyrininus*	*sculpturatus*	(Mjöberg, 1905)	0.126–0.012 Ma	Pleistocene	Härnösand, SE
Gyrininae	*Miodineutes*	*insignis*	(Heer, 1862)	11.62–7.26 Ma	Miocene	Öhningen, CH
Gyrininae	*Dineutus*	*longiventris*	Heer, 1862	11.62–7.26 Ma	Miocene	Öhningen, CH
Gyrininae	*Miodineutes*	*heeri*	Hatch, 1927	11.62–7.26 Ma	Miocene	Öhningen, CH
Gyrininae	*Miodineutes*	*oeningenensis*	Hatch, 1927	11.62–7.26 Ma	Miocene	Öhningen, CH
Gyrininae	*Gyrinus*	*aquisextanea*	Nel, 1989	28.4–23.0 Ma	Oligocene	Gypsum level of Aix, Bouches-du-Rhone, FR
Gyrininae	*Gyretes*	*giganteus*	(Piton, 1940)	33.9–23.0 Ma	Oligocene	Menat, Pay-de-Dome, FR
Gyrininae	*Orectochilus*	*sp*	Nel, 1989	33.9–23.0 Ma	Oligocene	Menat, Pay-de-Dome, FR
Gyrininae	*Gyrinoides*	*limbatus*	Motschulsky, 1856	37.2–33.9 Ma	Oligocene	Baltic Amber, Europe
Gyrininae	*Mesodineutes*	*amurensis*	Ponomarenko, 1977	66.0–61.7 Ma	Paleocene	Arkhara Site, Darmakan Form., RU
Heterogyrinae	*Cretotortor*	*archarensis*	Ponomarenko, 1977	66.0–61.7 Ma	Paleocene	Arkhara Site, Darmakan Form., RU
Heterogyrinae	*Cretotortor*	*zherichini*	Ponomarenko, 1973	93.9–89.8 Ma	Upper Cretaceous	Kzyl-Zhar dep. NE Karatau Range, KZ
Spanglerogyrinae	*Angarogyrus*	*mongolicus*	Ponomarenko, 1986	125.0–113.0 Ma	Lower Cretaceous	Gurvan-Eren Form., Govi-Altai, MN
Heterogyrinae	*Mesogyrus*	*striatus*	Ponomarenko, 1973	125.0–113.0 Ma	Lower Cretaceous	Turga Form., Undurga River, RU
Heterogyrinae	*Baissogyrus*	*savilovi*	Ponomarenko, 1973	125.0–113.0 Ma	Lower Cretaceous	Zaza Form., Buryatia, RU
Heterogyrinae	*Mesogyrus*	*anglicus*	Ponomarenko *et al*., 2005	145.0–140.2 Ma	Lower Cretaceous	Durlston Form., Dorset England, UK
Heterogyrinae	*Mesogyrus*	*elongatus*	Ponomarenko, 2014	152.1–145.0 Ma	Upper Jurassic	Sharteg Form., Altai Prov. MN
Heterogyrinae	*Mesogyrus*	*antiquus*	Ponomarenko, 1973	166.1–157.3 Ma	Upper Jurassic	Karabastau Form., Karatau-Mikhailovka, KZ
Heterogyrinae	*Cretotortor*	*sp*	Nel, 1989	182.7–174.1 Ma	Lower Jurassic	Liège dep. N°IB 974, Bascharage, LU
Spanglerogyrinae	*Angarogyrus*	*minimus*	Ponomarenko, 1977	182.7–174.1 Ma	Lower Jurassic	Cheremkhovskaya Form., Irkutsk, RU
Heterogyrinae	*Mesogyrus*	*sibiricus*	Ponomarenko, 1985	189.6–182.7 Ma	Lower Jurassic	Abasheva Form., Lyagush’ye, RU

## Results

**Figure 1:**
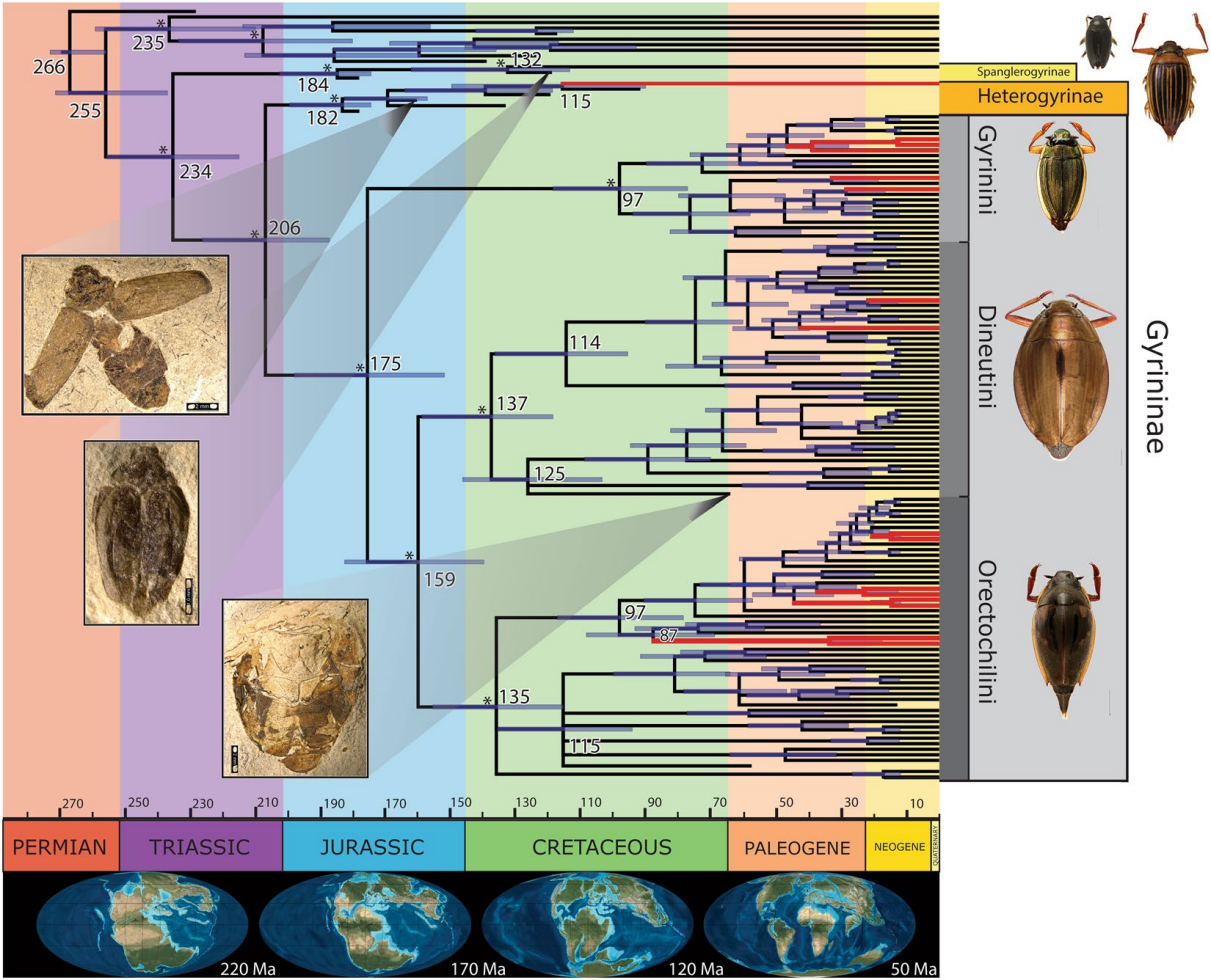
Total evidence dated phylogeny of Gyrinidae. Majority-rule consensus tree from Bayesian analysis using fossils as terminals under the FBD tree prior. Dates at selected nodes represent median posterior age estimate and blue bars show the 95% highest posterior density age range. The asterisk above nodes indicates support of ≥0.95 posterior probability (only indicated for basal nodes). Malagasy lineages are coloured red. Fossils and imaged exemplars not to scale. The paleogeographic maps below© 2012 Colorado Plateau Geosystems used with permission.

**Figure 2:**
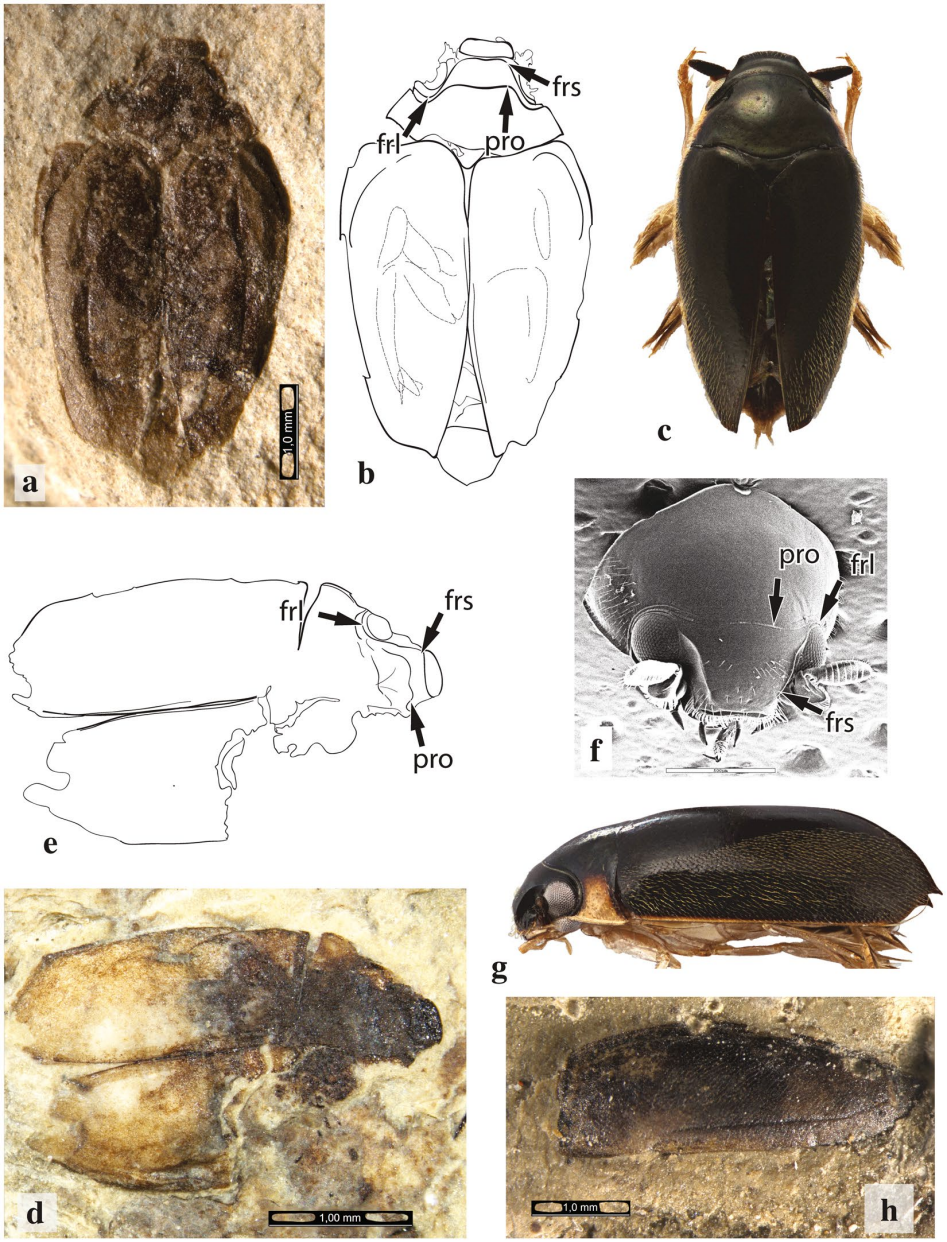
Fossil and extant species of Spanglerogyrinae. Arrows indicate important morphological features, abbreviations pro = pronotum, frs = frons, frl = frons lateral margin. (**a**) *Angarogyrus mongolicus* fossil no. 3149/970; (**b**) line drawing of specimen. (**c**) *Spanglerogyrus albiventris* dorsal habitus, scale bar = 1 mm. (**d**) *Angarogyrus minimus* fossil no. 1670/385, scale bar = 1 mm; (**e**), line drawing of specimen. (**f**) scanning electron microscope (SEM) image of *Spanglerogyrus albiventris* pronotum and head, scale bar = 500 μm. (**g**) *Spanglerogyrus albiventris* lateral habitus to scale with (**c**). (**h**) *Angarogyrus minimus* elytron fossil no. 1670/385 scale bar = 1 mm.

The analysis resulted in a well-resolved phylogeny of extant and extinct taxa with all higher-level relationships discussed below strongly supported (posterior probability 0.98–1.00; Fig. 1 and Fig. S1). The tree was consistently stable across methods of analysis, models, and priors (Supplementary Materials). Notably, not a single fossil was recovered as stem taxa to extant Gyrinidae. Two Mesozoic fossils described in the genus *Angarogyrus*, with previous uncertain affinity, grouped with *Spanglerogyrus*, and this clade (hereafter Spanglerogyrinae) was sister to a clade with the remaining extant and fossil Gyrinidae. Re-examination of the *Angarogyrus* fossils (Fig. 2a and d) reveals remarkable similarity to the living *Spanglerogyrus* (Fig. 2c). Both have a unique quadrate frons, with the frontolateral margins continued posteriorly over the dorsal eye (Fig. 2b,e,f), a pronotum with a strong medial lobe projecting anteriorly onto the head capsule (Fig. 2b,e,f), and a very small body size (Fig. 2a,c,d). An exceptionally well-preserved elytron of the fossil *Angarogyrus minimus* shows a similar coloration and covering of setae to the modern *Spanglerogyrus albiventris* (Fig. 2g and h).

**Figure 3:**
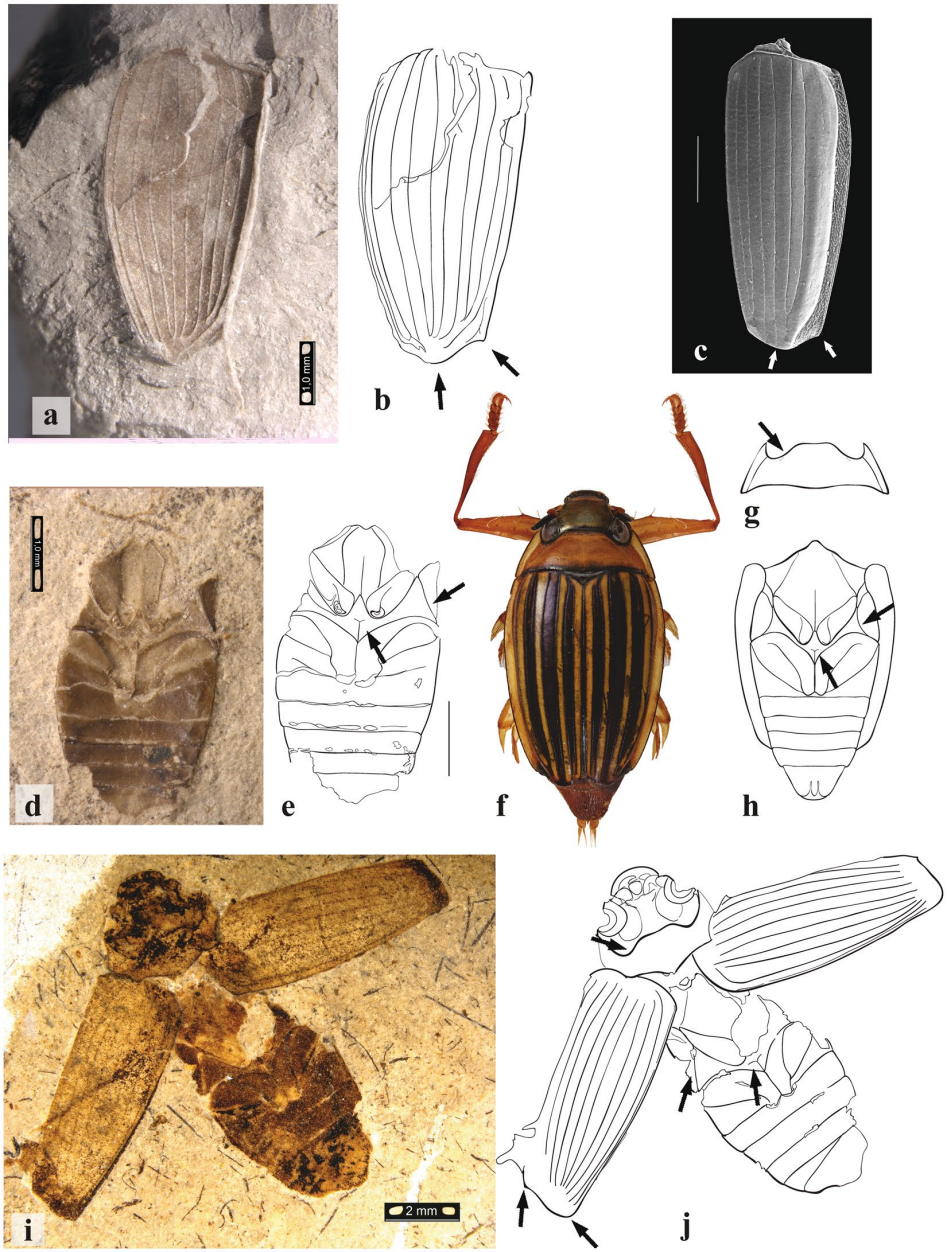
Fossil and extant species of Heterogyrinae. Arrows indicate important morphological features. (**a**) *Cretotortor zherichini* elytron fossil no. 3149/970; (**b**) line drawing of specimen. (**c**) *Heterogyrus milloti* elytron SEM, scale bar = 1 mm. (**d**) *Baissogyrus savilovi* holotype fossil no. 1668/1787, scale bar = 1 mm; (**e**) line drawing of specimen. (**f**) *H*. *milloti* dorsal habitus, scale bar 2 mm; (**g**), pronotum of *H*. *milloti*; (**h**), thorax and abdomen of *H*. *milloti* ventral view, scale bar = 2 mm. (**i**) *Mesogyrus antiquus* fossil no. 2997/1846, scale bar = 2 mm; (**j**), line drawing of specimen.

**Figure 4:**
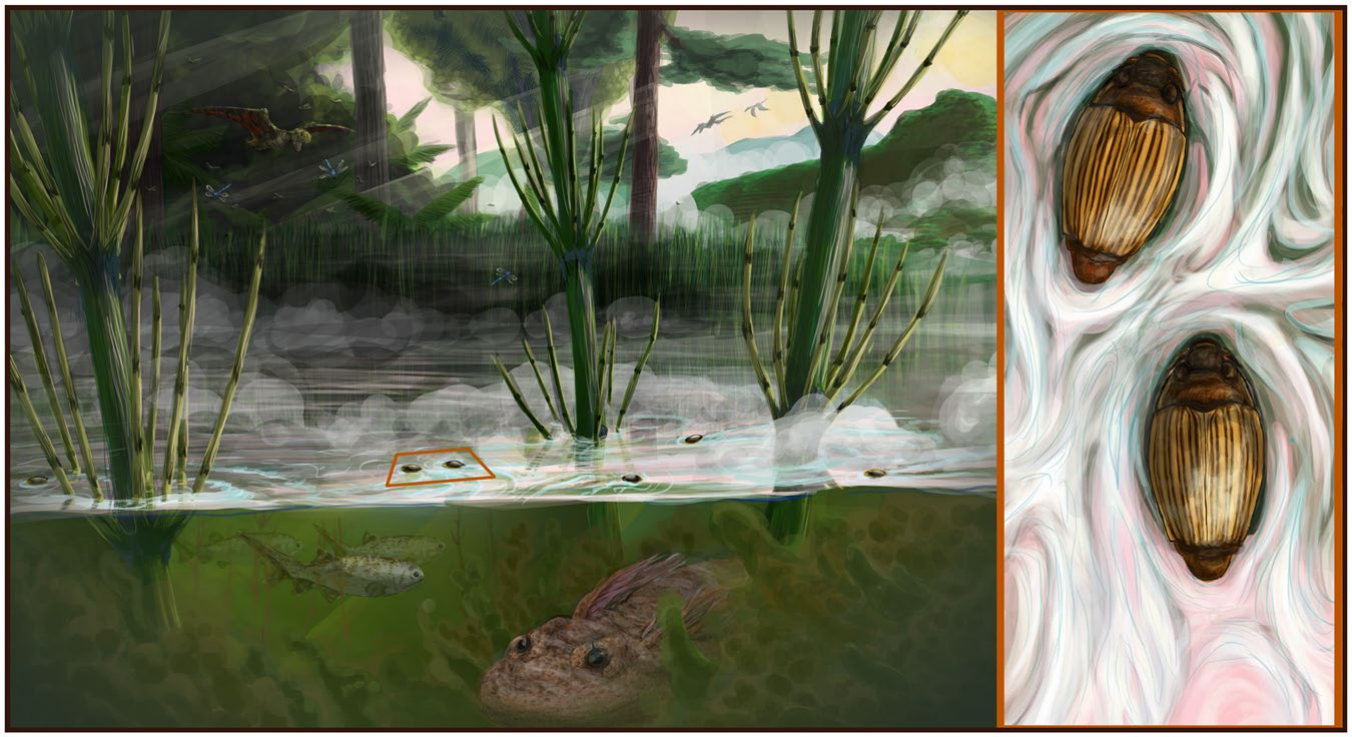
Habitat reconstruction of *Mesogyrus antiquus* from the Karabastau Formation. The Karabastau formation is the remains of a large, permanent, mountain lake from the Late Jurassic of Karatau, Kazakhstan. In the background the pterosaur, *Batrachognathus volans*, chases an odonate over the lake, while on waters’ surface the heterogyrine whirligig, *Mesogyrus antiquus*, spins about. Beneath, some *Pteroniscus* fish swim by, while the salamander *Karaurus sharovi* settles into the sediment. The right panel shows the dorsal habitus of *M*. *antiquus* in detail. Artwork by Trevor Fristoe.

Five other Mesozoic fossils described in the genera *Cretotortor*, *Baissogyrus*, and *Mesogyrus* were monophyletic together with the extant Malagasy genus *Heterogyrus* (hereafter Heterogyrinae). Heterogyrinae was sister to the subfamily Gyrininae, including all other extant taxa, as well as three Cenozoic fossils of the genera *Miodineutes*, *Mesodineutes* and *Gyretes*. Morphology of the well-preserved fossil *Mesogyrus antiquus* (Fig. 3i and j) from the Karatau deposits^26^, shows marked similarity to *Heterogyus milloti* (Fig. 3f,g,h) including a short transverse sulcus of the metaventral discrimen, the triangular lateral wing of the metaventrite, the lobiform metanepisternum, and the elytron with nine elytral striae and sutural border. The Karatau deposits are the remains of a large, stable, freshwater, Jurassic lake^27^ (Fig. 4). This indicates *M*. *antiquus* was lentic, compared to the extant *H*. *milloti*, known only from small trickling forest streams^22^, suggesting increased ecological diversity in the past. The heterogyrine genera were widely distributed and found throughout the Jurassic and Cretaceous (Table 1), forming the dominant gyrinid fauna at that time. That all fossil heterogyrines are Laurasian is very likely a result of sampling bias, as the vast majority of known Mesozoic insect deposits are Palearctic^26^. The origin of Gyrinidae was estimated to be Late Permian or early Triassic at 255 Ma (95% highest posterior density 236–271 Ma). Divergence of Spanglerogyrinae from the remaining Gyrinidae was dated to be Triassic at 234 Ma (214–255 Ma). The divergence of Heterogyrinae from Gyrininae was dated to the Late Triassic or Early Jurassic at 206 Ma (187–226 Ma).

Previous TED analyses have, in some cases, resulted in unrealistically old ages, postulating extremely long ghost lineages with no trace in the fossil record^28, 29^. This has been attributed to the type of relaxed clock model, morphological-molecular data conflict, and not accounting for systematic tip sampling bias^28, 30^. We therefore tested the stability of estimated divergence times extensively (Supplementary Materials). The largest impact on divergence time estimation was the tree prior (uniform instead of FBD), which gave significantly older ages (Heterogyrinae-Gyrininae divergence: 248 Ma; Supplementary Materials), similar to ref. 30. Standard node calibration analyses with fossils excluded as terminal taxa gave 21–24 Ma younger estimates but still early Jurassic (182–185 Ma) for the Heterogyrinae-Gyrininae divergence (Supplementary Materials).

## Discussion

A distinct transition is seen in the fossil record of whirligig beetles following the K-Pg boundary (Table 1). The Heterogyrinae are replaced by the Gyrininae as the dominant whirligig beetle fauna, following the end Cretaceous extinction event. At the Arkhara site, bordering the K-Pg boundary, heterogyrines and gyrinines co-occur, but all younger fossils are gyrinines, with all older fossils being heterogyrines, apart from two spanglerogyrines. By the time of the K-Pg boundary, Madagascar was already isolated, as India had previously drifted away from the IndoMadagascan subcontinent^2, 7^. Therefore, as an island Madagascar likely served as a refugium allowing the last heterogyrine lineage’s persistence through the K-Pg extinction event, while all other continental relatives went extinct and were replaced by gyrinines. Madagascar’s gyrinine fauna show a typical colonization pattern, diverging from African sister taxa predominantly during the Cenozoic through oversea dispersal (Fig. 1). The current distribution of *Heterogyrus milloti* is limited to humid mountain forests of Andringitra and Ranomafana, in the southern part of the eastern escarpment of Madagascar. It is only encountered in small forest streams, where no other gyrinids are found. A variety of gyrinine species occupy the larger streams, rivers, and ponds in the surrounding area at both lower and higher elevations. Given fossil heterogyrines occupied habitats similar to todays’ gyrinines (Table 1), such as large lakes (i.e. Karabastau –Fig. 4, and Sharteg formation), as well as fluvial deposits (Kzyl-Zhar deposits), the current habitat of *Heterogyrus milloti* likely represents a final marginalized stronghold for this relictual species following the arrival of gyrinines in the Cenezoic.

**Figure 5:**
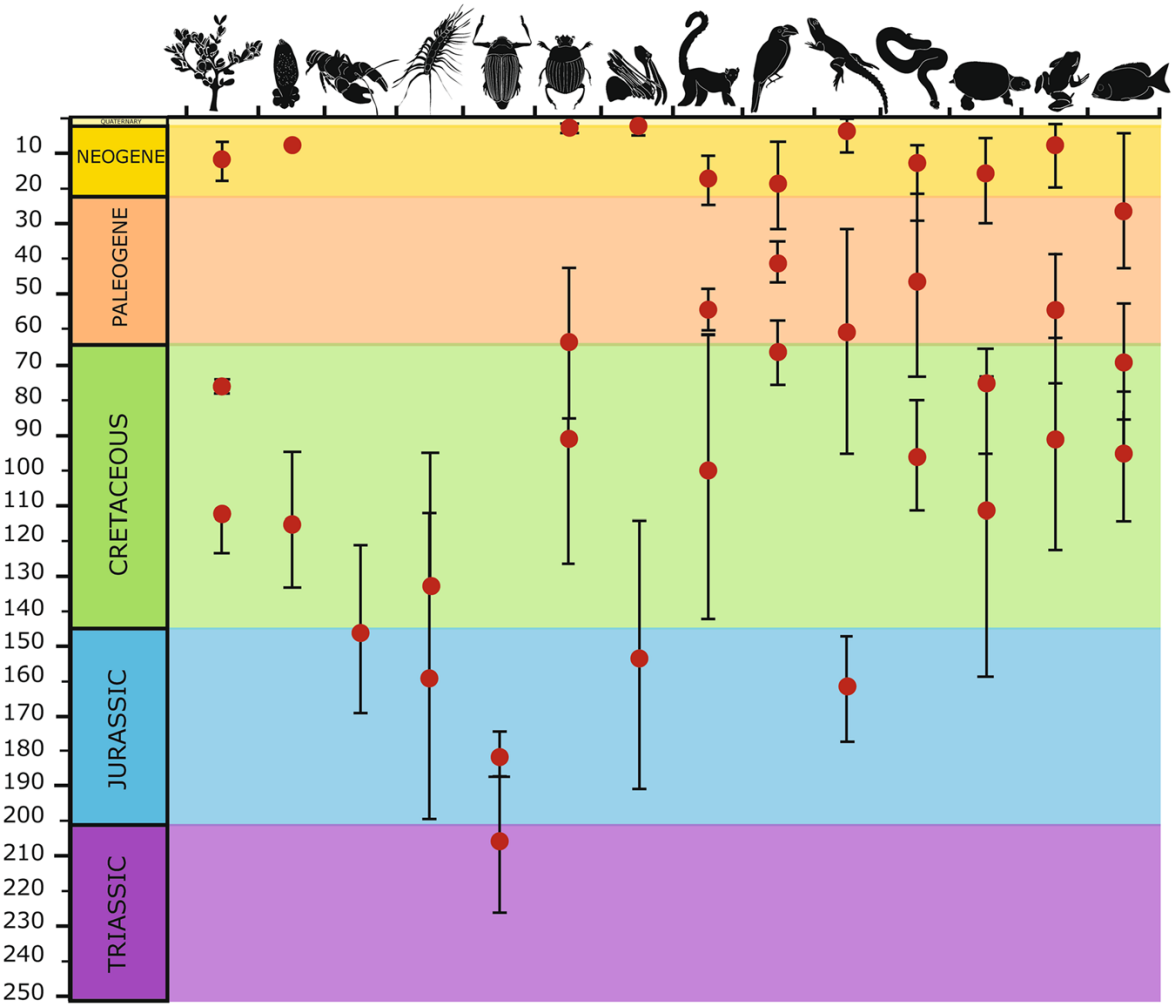
Summary diagram of age estimates of endemic Malagasy lineages. Within each group of organisms the oldest, a middle, and youngest estimated clade ages are depicted. Red dot is the median age estimate and error bars are the min and max intervals from the original study compiled from the literature (Table S2). Only those lineages with median, minimum and maximum age estimates were included in the figure. Groups from left to right are plants, trematodes, decapods, centipedes, heterogyrine whirligig beetles (showing tip and node dating ages), insects (excluding whirligig beetles), spiders, mammals, birds, lizards, snakes, testudines, amphibians, and fish.

At 206 Ma (187–226 Ma), *Heterogyrus milloti* is the oldest dated endemic lineage on Madagascar (Fig. 5; Table S2) and breaks a conceptual barrier rooted in the scientific community. Limited to discourse on the relative frequency of dispersal versus vicariance origins^2, 6, 7^, accounting for the fact that islands can serve as refugia from continental extinction, vicariance from India (88 Ma) or even Africa (160–130 Ma)^2^, is not necessarily the upper limit for Madagascar’s ‘paleoendemics’. Oplurid lizards, podocnemid turtles, xenotyphlopid blind and boid snakes, mantellid and microhylid frogs, and some cichlid fish, are the oldest endemic vertebrate clades (Fig. 5); but these are all at most Cretaceous or late Jurassic in age^5–8^. A few examples of endemic arthropod lineages in Madagascar (Fig. 5), such as astacoid freshwater crayfish^10^, scutigerine centipedes^11^ and archaeid spiders^9^, are older, dated to the Mid- or Late Jurassic. Vascular plants of Madagascar, although rich in endemism at higher taxonomic levels, have but a few examples of Cretaceous ancestry^31^. In contrast, *Heterogyrus milloti* diverged from its closest living continental relative before the Gondwanan break-up. This is unprecedented in Madagascar but echoes the survival of the Tuatara on New Zealand^32^. Both represent the last surviving species of formerly widespread Triassic-Jurassic lineages ‘rescued’ from extinction by microcontinental islands. Madagascar serving as such a refugium sets the island in a new perspective and demonstrates that increased attention to arthropods will likely change our view of this famous natural laboratory of evolution.

## Methods

### Morphological data

Fourteen fossil taxa were included in the analysis, four representing outgroup taxa, and ten ingroup taxa, representing almost 50% of the known, unambiguous, gyrinid fossils (Table S3, Table 1). Fossil taxa were selected for inclusion based on availability and preservation of morphological features. Some described fossil gyrinid species could not be located in the indicated depository, such as *Dineutus longiventris* Heer, 1862 and *Gyrinoides limbatus* Motschulsky, 1856, and were unavailable for study. Gyrinid taxa described only from elytra (e.g. *Protogyrininus sculpturatus*, *Mesogyrus anglicus*, *M*. *elongatus*, *M*. *sibiricus*, *Cretotortor archarensis*) were not included in the final analysis, due to introduction of significant phylogenetic uncertainty. However, the elytral characters present in these fossils were accounted for through congeners (*Protogyrininus* is likely a synonym of *Gyrinus*) included in the analysis, which presented additional morphology. In total all unambiguous fossil gyrinid genera except one, *Gyrinoides*, were analyzed. A complete list of the fossil specimens included in the analysis and their depositories is provided in Table S3. The fossil *Coptoclava longipoda* was originally included in the dataset but was removed from all analyses except one due to ambiguous coptoclavid monophyly and affinity^17^. The result of the analysis with *C*. *longipoda* included is discussed in the Supplementary Materials.

Extant taxa were coded from specimens included in the study from ref. 22 and those listed in Table S4. Extant specimens were examined using a Zeiss Discovery.V8 SteREO microscope, as well as a scanning electron microscope. A novel morphological character set of 120 characters was generated (see Character Coding under Supplementary Materials) with fossil and extant taxa coded as in Table S1.

### Molecular data

Thirty-three additional ingroup gyrinid taxa and one outgroup taxon, *Haliplus lineatocollis*, were added to the five-gene dataset from ref. 22 (Table S4). Additional taxa included sequences for all genes except EF1α which was found to have multiple copies in Gyrinidae^22^. In total, taxon sampling (extinct and extant taxa) for the analysis included ten outgroup taxa from Hydradephaga and 129 gyrinid taxa. The same primers were used as in ref. 22. New sequences are deposited in Genbank with accession numbers available from Table S4.

### Phylogenetic analyses

All bayesian phylogenetic analyses were implemented using the MPI version of MrBayes 3.2.6^33^ and were run on the super computer cluster ‘Ulam’ at the Center for Advanced Research Computing (CARC), University of New Mexico. The same 8-part partitioning scheme was used as in ref. 22. Each molecular partition was allowed a separate gamma distributed rate variation across sites parameter. Reversible-jump MCMC was used to integrate over the 203 possible symmetrical substitution rate models during analysis^33, 34^. The morphological partition was given a Markov k model^35^, accounting for that only parsimony informative characters were included, and with a gamma shaped rate variation parameter across characters. Fifteen characters were treated as ordered (see Supplemental Material). A topological constraint defined Adephaga, all ingroup and outgroup taxa except *Triaplus laticoxa*, as re- examination of the fossil material suggests Triaplidae is not a member of Adephaga as previously proposed^20^. Each analysis included two independent runs starting at random topologies, each with one cold and three incrementally heated chains (temp = 0.1) run for 20 million generations, sampling every 1000th generation. MCMC convergence was monitored using Tracer v.1.6^36^ and statistics provided by MrBayes^33^.

### Clocks and calibration

For a detailed discussion on the total evidence dating (TED)^23, 28, 30^ under the fossilized birth- death (FBD)^25^ process model and the sensitivity analyses performed, see Supplementary Materials. In all analysis, TED as well as node dating, topology and divergence times were inferred simultaneously, hence our inferred divergence times are not dependent on a topology presumed to be known without error. To set a prior on the base clock rate the method outlined by ref. 23 was followed. Here the tree height in units of expected number of substitutions per site from root to tips is estimated under a strict clock as an average across partitions, while making sure the root prior is unimportant (exp (0.1), (1.0) or (10)). The median of the tree height is divided by the minimum and maximum age of the root based on fossils to get a base clock rate interval in substitutions per site per million years. The age of the earliest Tshekardocoleidae (c. 298 Ma) the oldest known coleopteran^20, 37, 38^ was used to set a lower substitution rate level, and the min age of *Triaplus laticoxa* (Min age 221 Ma), the oldest fossil included in the dataset and hence the minimum age of the root, was used to inform on a maximum likely substitution rate. This guided the design of a lognormal (−5.7, 0.3) prior on the base clock rate. The tree prior was set to FBD with beta (1, 1) priors for the [0–1 interval] parameters (turnover and fossil sampling proportion) and an exp (10) prior for the net diversification parameter^25^. The proportion of sampled tips was set to 0.1 as about 10% of known species (ca. 1000) were sampled. The FBD tree prior is contingent on a root age, tmrca^25^. We used a uniform prior between 252–273 Ma for the root based on the oldest known Triaplidae fossil and the mid to late Permian boundary which also defines the border between only reticulated beetle elytra and the first smooth beetle elytra (see Supplementary Materials).

We used the uncorrelated relaxed clock model IGR^33, 39^ in MrBayes with the prior on rate variation across lineages set to exponential (10). The fossil terminals were assigned uniform age priors based on the dated period of respective fossil deposit (Table S3). Sampling assumption was set to diversified sampling for extant tips^30, 40^.

### Sensitivity analysis

To test the sensitivity of inferred divergence time estimates to model and parameter settings we ran a series of additional analysis (for full details see Supplementary Materials). The clock rate variation among lineages parameter was run with priors exp (1), (10) and (100). The variation of the base clock rate prior was increased to lognormal (−5.7, 0.6). The root age tmrca was set to a conservative uniform prior of [221 Ma − 298 Ma] based on oldest included fossil, and oldest known Coleoptera, as well as to offset exponential priors with offsets at 221 or 252 Ma. The net diversification prior of the FBD was run under an exponential (1), (10) and (100) prior. The sampling proportion of extant species was reduced to 0.01 to account for the possibility of a large proportion of unknown cryptic diversity. We ran an autocorrelated relaxed clock model (TK02)^33, 41^ together with the FBD tree prior as well as both uncorrelated (IGR) and autocorrrelated (TK02) clock models under a uniform tree prior. We allowed the FBD process to be piecewise divided into two or three time intervals^30, 42^. Finally, we tested the effect on the topology and clade support of excluding the fossils and compared the divergence time estimates from TED with a standard node dating approach^28, 29^. In the node dating analysis, we first used the inferred fossil placements from a non-clock analysis. Then we constrained the nodes Orectochilini, Dineutini and Heterogyrinae + Gyrininae to be monophyletic and set offset exponential calibration priors on these three nodes using the three oldest fossils from respective clades as the offset values (see Supplementary Materials). To be directly comparable with the TED analysis we ran this node dating analysis with an FBD tree prior but fixed the fossilization rate to 0 (no fossils sampled) since fossils were excluded as terminal taxa in this analysis.

### Literature review

We performed a literature review through 2016 of studies dating extant endemic Malagasy lineages of animal and plants, to compare ages. Multiple estimates for the same taxon were included, as were different dating techniques. The compiled age data are available in Table S2 of the Supplemental Materials.

## Electronic supplementary material

Supplementary Material
