# Supplementary material for: Tip-dated phylogeny of whirligig beetles reveals ancient lineage surviving on Madagascar
Source: Sci Rep. 2017 Aug 22;7:8619. doi: 10.1038/s41598-017-08403-1 (PMC5567340; doi:10.1038/s41598-017-08403-1)
Supplement: Supplementary file 1 — Supplementary Material [file 41598_2017_8403_MOESM1_ESM.pdf]

Tip-dated phylogeny of whirligig beetles reveals ancient lineage surviving on Madagascar

Grey T. Gustafson[1*], Alexander A. Prokin[2], Rasa Bukontaite[3], Johannes Bergsten[3], Kelly B. Miller[4]

Affiliations:

[1]Department of Ecology and Evolutionary Biology, University of Kanas, Lawrence, KS, 66046, USA.

[2]Papanin Institute for Biology of Inland Waters, Russian Academy of Sciences, Borok, Nekouzskii District, Yaroslavl Region, 152742, Russia.

[3]Department of Zoology, Swedish Museum of Natural History, Box 50007, SE-104 05 Stockholm, Sweden.

[4]Department of Biology and Museum of Southwestern Biology, University of New Mexico, Albuquerque, NM 87131, USA.

*correspondance to: gtgustafson@gmail.com

Supplementary Materials

SUPPLEMENTARY TEXT

Bayesian Phylogenetic Dating

Methods for species divergence time estimation are continuously improving and have taken a number of significant steps since the original postulation of the molecular clock hypothesis (*44, 45;* review by *46,47*). The relaxation of the strict clock hypothesis to allow for rate variation across lineages was one significant step leading to more realistic clock models and inferences (*48*). Allowing for the uncertainty of both the age estimates of fossil layers, and how the fossil information translates to hard upper but soft lower node age bounds was another significant step (*49*). This led to the last ten year's paradigm of defining prior distributions for node age calibrations such as the exponential, lognormal and gamma distributions (*50,51*).

The latest development showing great promise for taking yet another significant step forward can be referred to by firstly total-evidence dating (TED, also known as tip dating) and secondly the fossilized birth-death prior (or FBD). Total-evidence dating integrates over the uncertainty of fossil placement in the tree by co-estimating the topology and divergence times simultaneously using a morphological data matrix scored for both fossils and extant taxa, and commonly a molecular dataset for only extant taxa (*23*,*52*). Fossils are here included as terminals just as

extant taxa, while the fossil ages helps to date the tree and the morphological data helps estimating fossil branch lengths. Apart from accomodating the uncertainty related to the fossil placement in the tree, compared to node-dating total-evidence dating also circumvents i) the need for often arbitrarily defining the soft upper bounds on prior node age calibrations, ii) the need for topological constraints on calibration nodes which are of course never known with certainty, iii) discarding evidence in the fossil record since in node-dating only the oldest fossil for each clade is of any use (*23*).

The fossilised birth-death prior shares some of the same advantages as total-evidence dating - all fossils are potentially useful for instance, not just the oldest one for each clade. The FBD is an attempt to improve the treeprior (which is also a prior on node times) used for dating analyses, and recognises that extant and fossil taxa are all part of the same macroevolutionary diversification process involving speciation and extinction (*53*, *25*). In node-dating, the external node calibrations and the information on node times from the treeprior may conflict or interact in ways not realized by the user and significantly influence dating results (*54-56*). The FBD process model act as a more appropriate treeprior for dating analyses with multiple fossils and requires parameters for speciation rate, extinction rate, fossil recovery rate and proportion of sampled extant species, along with an informative prior on root age (*25*). There is seldom information regarding the first three parameters why vague uninformative priors are commonly used here.

The last year has seen the two methodological advances combined -total evidence dating using the FBD as treeprior, and made available in packages such as MrBayes and Beast (*31*,*42*). We take advantage of these developments but also acknowledges that some empirical studies have seen unrealistic outcomes, in particular seemingly too old ages (*38* and references therein, *55*, *57*; see also *30*), why we also perform extensive testing of the stability of the results in light of varied prior and model settings as well as with traditional node dating. This also included testing for the effect of sampling assumptions which has been shown to affect dating analyses significantly (*30,31,45*).

<u>Preferred analysis</u>
In the preferred analysis 14 fossils were included as terminal taxa, scored for morphological characters and their ages given as a uniform priors with the upper and lower hard bounds given by the time period of the dated fossil layer (Table S3). The fossils included four outgroup fossils and ten ingroup fossils varying from Triassic to Neogene in age. No topological constraints or node calibrations were imposed apart from for the root which was given a uniform prior (252.3-272.3) and the Adephaga (all taxa except *Triaplus*) which was constrained as monophyletic. *Triaplus* of the family Triaplidae was previously considered an Adephagan family, but has recently been re-evaluated as an Archostemata (Prokin, unpublished data). The lower bound is based on the age of the oldest representative of the genus *Triaplus*, one representative of which was also included as terminal. This use rests on the assumption of a monophyletic Triaplidae which seems justified given the characteristic enlarged metacoxal plates paralleled by Haliplidae but without being closely related (*17*). The upper bound is set at the border between mid and early Permian, before which plenty of fossil beetles are known but none with smooth elytra like the Hydradephaga. All beetle fossils before mid Permian consist of Archostematan or Proto-Coleopteran fossils with lattice-like elytra (*20*). This possibility of a rather robust restricted and informative root prior puts us in a privileged analytical situation related to otherwise potentially

problematic "deep-root attraction" artefacts of TED (*30*, also see *34*). In the sensitivity analysis however, we also examined the effect of a less informative root prior.

Sampling assumption of extant taxa was set to diversified sampling (see *30*, *31*, *35*). For the base clockrate the method of ref [*23*] was used to calculate an appropriate prior on the average base clock rate. The prior was set to a lognormal distribution (-5.7, 0.3). We used the IGR relaxed clock model implemented in MrBayes (*26*) with the variance parameter set to exponential (10) in the preferred analysis, but also examined the effect of an autocorrelated relaxed clock model.

For the FBD parameter priors the sampling proportion was set to 0.1 as about 10% of the about 1000 known species of Gyrinids were sampled. We also evaluated the effect of lowering this proportion to 0.01, assuming a large proportion of extant yet unknown or cryptic species. As we have no prior information on the speciation rate, extinction rate or fossilization rate, these were given vague priors as default in MrBayes (3.2.6). The re-parametrization of the model into a net diversification, a turnover and a fossil sampling proportion (*36, 58*), means that two of the parameters are on the interval of 0-1, and were given a beta (1,1) prior, and only the net diversification parameter is on the 0-infinity range. The latter was given an exponential (10) prior but the effect of changing this prior was evaluated. In the preferred analysis we used a single timeperiod, but in the sensitivity analysis we examined the effect of letting the FBD process vary across time in a piecewise manner (*31*, *37*).

Coptoclava longipoda, a representative of the extinct Coptoclavids was excluded in the preferred and in all sensitivity analyses except one due to questioned monophyly and affinity of Coptoclavids (*17*). Including *C. longipoda* did not affect the support for any of the major ingroup clades (0.98-1.00 as in the preferred analysis), nor the estimated divergence times (a maximum difference of 8 my for the monitored nodes compared to the preferred analysis). The phylogenetic position for *C. longipoda* was recovered as sister to Gyrinidae but with poor support (0.68).

Sensitivity analysis

FBD parameters

First we examined the effect of priors on the FBD parameters. Varying the prior on the Net diversification using an exponential distribution with a rate parameter of 1, 10 or 100 had negligible effect on estimated species divergence times (Table S5). Assuming a ten fold increase in unknown or cryptic extant species diversity by changing the sampling proportion prior to 0.01 likewise had a small effect towards younger ages (crown Gyrinidae 10my younger, Heterogyrinae-Gyrininae divergence 8my younger). The same was true for changing the standard FBD to a piecewise model with two or three time intervals. Our included fossils, which were based on how well morphological characters could be scored, are unevenly distributed over time with most from the Cretaceous. Dividing the FBD process into a Paleogene, a Cretaceous-Jurassic and a Triassic time slices had little effect on estimated ages (maximum a 9my difference) even though the posterior estimate of the fossil sampling proportion differed with several orders of magnitude (3 timeslices: slice 1: Mean=0.228 (variance= 0.044), slice 2: Mean=0.013 (var=0.0029), slice 3: Mean=0.00011 (var< 0.000001)).

Relaxed clock models, base clock rate and rate variance across branches

The prior for the clock base rate was set following the method outlined in ref [*23*]. First a posterior estimate of the tree height was inferred under a strict clock model with the base rate fixed to 1. This way the treeheight represents the number of substitutions per site from root to tips as an average across all gene partitions. The analysis was run under a treeheight (root) prior of an exponential distribution (1), (0.1) and (10) which showed that this only had a very marginal effect on the treeheight (0.90-0.93). The treeheight posterior estimate for exp (1), 0.92 (95% HPD: 0.82-1.03) was used and divided by the expected [252-273 - see discussion above], minimum [221 - age of oldest included fossil in the tree] and maximum [299 - age of oldest (proto)Coleopteran fossil] root age estimate to inform on an average substitution rate for the dataset. This resulted in a substitution rate of 0.0033-0.0037 with upper and lower limits being 0.0028 to 0.0047. From this we defined a lognormal prior distribution on the base clock rate for the preferred analysis with log mean -5.7 and log stdev 0.3 which gives a distribution with median 0.0035 and the 5 and 95% quantiles 0.002 and 0.0055. To test the effect of a broader range for the base clock rate prior we also ran an analysis with lognormal (-5.7, 0.6) which gives a distribution with the 5 and 95% quantiles of 0.0012 and 0.0090. This had a negligible effect on divergence time estimates (Table S6). The uncorrelated relaxed IGR clock model has a parameter IGRvar defining the amount of rate variance across branches. In the preferred analysis we set the default exp(10) as a prior on this parameter, but changing this prior to exp(1) or exp(100) was likewise without effect for the divergence time estimates (Table S6). Finally, there are two main types of relaxed clock models often discussed and compared - autocorrelated models and uncorrelated models (*34, 48, 59*). IGR belong to the latter category of uncorrelated models (*26, 34*). Auto-correlated models assume some degree of inherited rates from ancestral to descendant nodes so that parent-daughter branch rates are correlated. It seems that whether an autocorrelated or an uncorrelated relaxed clock model is more appropriate is dataset dependent (*48, 59*). Likely, the signature of rate correlation between parent and daughter nodes is stronger in datasets of closely related species, whereas in datasets on deeper relationships (like this one), the signature may disappear (*48*; also see *23*). Running the analysis under the autocorrelated relaxed TK02 clock model (*26*, *36*) had negligible effect on focal nodes but the tribe Gyrinini was pushed back in time (134 my instead of 98 my) whereas Orectochilini was younger (107 my instead of 136 my) (Table S6). The autocorrelated models have a smoothing effect as rapid rate changes are not allowed over the tree (*23*).

Root age prior

As discussed above we were quite privileged by the fossil record allowing us to set an informed root prior with a range of just 20my equivalent to second half of Permian. However, the upper bound, even with the argument already presented, is derived from negative evidence, i.e. no smooth beetle elytra fossils known prior to this time. Due to the well-known poor, biased and inadequate preservation history for many groups in the fossil record it is always a risk interpreting negative evidence as "not yet present" (*47*). Likewise the lower bound is based on a dated layer with some surrounding controversy (although only if the layer is very latest Permian or very earliest Triassic and hence with little impact here), and rest on the assumption of a monophyletic Triaplidae with respect to the sampled extant ingroup and outgroup taxa. Finally, the hard upper bound of a simple uniform prior can be criticised in favor of distributions with soft upper bounds like the exponential. The root prior is a critical parameter that can have large

effects on inferred ages. The FBD model prior is also explicitly conditional on a root age (*25*), as is most other dating methods e.g. TED dating under a uniform treeprior (*23*). We relaxed all of these assumptions in a series of analyses testing the effect of changing the root prior (Table S7). Despite its importance and previously reported examples of significant effects (e.g. *57*), widening the root prior range with either a uniform or a exponential prior had the effect on divergence time estimates limited to changes of less than 20 my older for key clades (Table S7). With the very wide and conservative uniform root prior [221-299my], this changed the estimates most towards older ages: Gyrinidae crown clade=254my instead of 235my and the divergence between Heterogyrinae and Gyrininae 220my instead of 206my.
As expected the difference is greatest at the root node and smallest for nodes close to fossil terminal taxa like for crown Spanglerogyrinae and crown Heterogyrinae.

<u>Sampling assumption</u>
Ref [*35*] showed that the prior assumption on how taxa in a dataset had been sampled could have a large effect on estimated speciation and extinction rates under birth-death models. Whereas the standard models have assumed a random sampling of taxa, in practice it is rather a rule that phylogenetic datasets are sampled to maximise the diversity in the group, i.e. a far from random sample of the clade. This is also the case for the present Gyrinidae dataset. The probability of sampling both Heterogyrinae and Spanglerogyrinae in our dataset if around 100 species were sampled at random from the approx. 1000 available Gyrinidae species is about 0.01, i.e. not very likely. Ref [*31*] realized that this could also be important for divergence time estimations under the FBD prior and implemented the ref [*35*] accommodation of the more realistic diversified sampling into the model. For the Hymenoptera tree-of-life the random sampling assumption gave the age of Hymenoptera to 347my (early Carboniferous) under the FBD tree prior, but accounting for the intentionally diversified sampling of extant species in the dataset gave an age of 279My (early Permian) (*31*). With TED dating including fossils as tips under an FBD model with vague priors this assumption determined whether placental mammals were 118my old (diversified sampling) or 325my old (random sampling)! In our analyses, not accounting for that the dataset has been sampled to maximize the diversity resulted in somewhat older age estimates (Table S8). Under a random sampling assumption the age of crown Gyrinidae was estimated to 244my instead of 235 and the Heterogyrinae-Gyrininae split was estimated to 217 instead of 206my.
The FBD prior actually allows fossil to be inferred as direct ancestors to other fossil or to extant species, that is, to be inferred to sit directly on internodes rather than on side-branches. However in our analysis all fossils were estimated to sit on side branches and the probability of fossils as direct ancestors was low in most analysis (0.12 in preferred analysis, at most 0.25 in the 3 timeslice FBD analysis). Under the assumption that fossils do not leave any descendants (not allowing fossil to be direct ancestors), this had negligible effect and gave the same older node ages as random sampling (3my older) since it was combined with the random sampling assumption of extant species (Table S8).

<u>Treeprior</u>
The FBD treeprior was used in the preferred analysis and all sensitivity analyses above. But we also tested the effect of employing a non-mechanistic uniform treeprior where internal nodes are simply assumed to be uniformly distributed between root and tips. This prior does not involve

any speciation, extinction or fossilization rate parameters. This was the tree-prior used when ref [*23*] introduced total evidence dating treating fossils as terminal taxa. Later when ref [*31*] reanalysed the same Hymenoptera dataset under the FBD treeprior this resulted in either older or younger ages for Hymenoptera dependning on whether random or diversified sampling was assumed under the FBD (347 or 279 my compared to 306my under the uniform treeprior). We analysed the Gyrinidae dataset under a uniform treeprior and employing either an uncorrelated relaxed or an autocorrelated relaxed molecular clock (Table S9). This resulted in significantly older estimated divergence time ages in all cases. It is most appropriate to compare the estimated divergence times between the uniform and the FBD treeprior both under the IGR clock model (Table S9). However, perhaps the uniform treeprior in a sense lies closer to the FBD treeprior under random rather than under the preferred diversified sampling. The uniform treeprior gave somewhat older age estimates compared to FBD random for basal nodes (e.g. Gyrinidae crown 258 vs 244my, older age estimates for intermediate nodes (e.g. Heterogyrinae-Gyrininae split 248 vs 217 my) and much older estimates for more recent clades (e.g. Gyrinini crown 157 vs 113 my, Dineutini crown 211 vs 158 my). Since random sampling under the FBD already gave significantly older ages than in our preferred analysis under diversified sampling, the uniform treeprior estimates are much older than our preferred (Table S9).

Node dating

Since TED was introduced (*23, 52*), several empirical studies have reported that TED generally produces older ages than traditional node dating (*38,57*). Some studies also report that TED cause both too young and too old ages (*55*). The causes of these discrepancies has been evaluated and traced to both specific particularities of TED and effects that equally well applies to node-dating like not accounting for systematic tip-sampling bias, or imperfect relaxed clock models (*30*). The factors specific to TED largely relates to the modelling of morphological characters and arguments related to the 90's debate on the relative merits of molecular versus morphological characters (*47*), and the effect of missing characters in fossils (*38*). The difference here is its effect on divergence time estimation and not only the topology. In order to make sure that our divergence time estimates from TED analysis are not inappropriately more ancient that traditional node-dating analysis we also ran the latter using three node constraints. First we ran a non-clock analysis with fossils included to estimate their position. Second the oldest fossil for Orectochilini (*Gyretes giganteus* - Oligocene), Dineutini (*Mesodineutes amurensis* - Paleocene) and Heterogyrinae+Gyrininae (*Mesogyrus antiquus* - Jurassic) were used to set node calibration priors for respective nodes which were also constrained to be monophyletic; Heterogyrinae+Gyrininae - offsetexponential (min = 174, mean = 200), Dineutini - offsetexponential (min = 62, mean = 85), Orectochilini - offsetexponential (min = 56, mean = 80). As fossils were excluded (including the representative of Triaplidae) in the node calibration analyses, the prior on the root node deserves new attention. In principal, the same root age prior [252-273] could be used following ref [17] who inferred Triaplidae to be closer to Dytiscoidea than to Gyrinidae, but that would rest on their conclusions with poor support values for this placement of Triaplidae. We therefore ran three analyses with uniform root priors set to [253-273], [221-273] and [205-273]. The last prior we consider rather conservative and is based on the age of *Colymbotethis antecessor* a larvae considered to be a Dytiscoidea (*17, 60*). At the earliest Jurassic aquatic Coptoclavidae inferred as sister group to Dytiscoidea were also already well represented (*17*).

Node dating gave somewhat younger divergence time estimates compared to TED under all three root priors, but with broadly overlapping 95% highest posterior density intervals (Table S10). Crown age of Gyrinidae was dated to 213, 213 and 217 Ma under the three root priors compared to 235 Ma with TED. Heterogyrinae-Gyrininae divergence was dated to 182, 184 and 185 Ma under the three root priors compared with 206 Ma with TED. The difference is 21-24 million years younger, but the 95% highest posterior density intervals for the Heterogyrinae-Gyrininae divergence span late Triassic to early Jurassic in all node dating analyses just like with TED (Table S10).

Conclusions from the sensitivity analysis

In summary, the most important factors for divergence time estimation with this dataset are (changes for at least some monitored nodes of >20 my), i) the type of relaxed clock model, ii) the tree prior iii) the sampling assumption under the treeprior and iv) the dating type of method - in particular whether fossils are included as terminals or if only a subset is used to inform node age priors (TED vs. node dating). Similar conclusions were reached by references [*23*, *31*]. Node dating gave 21-24 Ma younger divergence time estimate while a uniform tree prior or a random sampling assumption, gave 11-42 Ma older estimates for the Heterogyrinae-Gyrininae divergence. The type of relaxed clock model did not affect the estimated Heterogyrinae-Gyrininae divergence time, only shallower nodes. Neither case jeopardizes our main conclusions. Notably the difference between node dating and TED is on a significantly smaller scale than commonly reported (*30, 57*). A common argument against including fossil as terminals is the higher proportion and non-randomness of missing data often resulting in poorly supported reconstructions (*31, 47*). This was not the case here as all fossils were resolved with strong support (>0.95 in posterior probability) to extant subfamilies or tribes. This rich and informative fossil record together with the opportunity for a comparatively narrow and informative root age prior likely helped to contain effects due to potential model misspecifications to relatively marginal sources of errors.

Descriptions of Morphological Characters

First number indicates the character number in the morphology matrix only. Numbers given in parentheses correspond to character numbers in total-evidence matrix found in the Nexus file.

Head

1 (3319). Head capsule shape excluding labrum. (0) elongate, longer than wide; (1) broad, wider than long. The head capsule of *Haliplus* and *Hygrobia* species are distinctly elongate, while those of the remaining species studied are clearly broad.

2 (3320). Divided eyes. (0) absent; (1) present. The eyes of two aquatic beetle families are clearly divided into a dorsal and ventral pair, the Coptoclavidae (*60*) and the Gyrinidae. All other species studied exhibit non-divided eyes.

3. (3321) Eyes. (0) bulging; (1) in contour with head. The eyes of most hydradephagans are in contour with the headcapsule. Bulging eyes not in-line with the contour of the headcapsule are present in *Haliplus* and *Hygrobria* species.

4 (3322). Eye division. (0) narrowly divided by thin canthus; (1) widely divided with well developed ocular ridge. *Spanglerogyrus* exhibits narrowly divided eyes separated by a thin canthus (*21*), while the remaining gyrinid species have widely divided eyes separated by a well-developed interorbital ridge (*61 ,62*).

5 (3323). Antennal form. (0) scape elongate and flagellum filiform; (1) compact flagellum, with an expanded pedicel and cup-like scape. The second character state describes the antennae unique to the family Gyrinidae.

6 (3324). Number of antennomeres in flagellum. (0) more than nine; (1) nine antennomeres; (2) eight antennomeres; (3) seven antennomeres; (4) six antennomeres.

7 (3325). Antennal flagellum apex with long setae. (0) absent; (1) present. *Spanglerogyrus* and *Heterogyrus* have antennal scape apices with long setae (fig SE and F). These setae are absent in the remaining Gyrinidae and the other Hydradephaga.

8 (3326). Posterior margin of clypeus. (0) complete; (1) incomplete. *Hygrobia* and the Gyrinidae have a complete posterior margin of the clypeus. In the other hydradephaga studied the clypeal posterior suture is partially effaced.

9 (3327). Ratio of the frontolateral margin to the width of the clypeus at mid-length. (0) frontolateral margin at least 1.5 times the longer than the medial clypeal width; (1) nearly equal or less than one. The frontolateral margin character is specific to the Gyrinidae studied. The frontolateral margin is elongate in *Spanglerogyrus*, *Heterogyrus*, in many orectochilines and dineutines. A reduction of the frons length is seen independently in several gyrinid such as the gyrinines, *Dineutus*, and Gyretes and some *Patrus*.

10 (3328). Lateral margin of frons with a well developed bead. (0) absent; (1) present. This bead appears in some Gyrinidae such as *Heterogyrus*, *Enhydrus*, *Macrogyrus*, and *Gyrinus*.

11 (3329). Frons swollen and quadrate with frontolateral ridge continued dorsally to caudal third of dorsal eye (*62*). (0) absent, fronts not swollen in appearance, frontolateral ridge not continued dorsally to caudal third of dorsal eye; (1) present. The distinctly swollen, quadrate frons (*21*) with the frontolateral margins continue dorsally to caudal third of dorsal eye (*62*) are unique to *Spanglerogyrus* and *Angarogyrus*.

12 (3330). Pseudofrontal ridge. (0) absent; (1) present, but narrow and weakly developed; (2) present and well developed, broad and often setose. The frons of orectochiline species has an additional lateral, depressed ridge, the pseudofrontal ridge (*63*). This ridge is unique to this tribe of whirligig beetles.

13 (3331). Labral shape. (0) transverse; (1) elongate. A transverse labrum is very common within the Hydradephaga and in these analyses a labrum is coded as being transverse if it is less than half as long as wide. An elongate labrum is defined as being at least half as long as wide. An elongate labrum is present in *Orectochilus*, *Orectogyrus*, *Porrorhynchus*.

14 (3332). Labral form. (0) quadrate; (1) rounded, including triangular; (2) emarginate. The *Spanglerogyrus* and *Angarogyrus* species possess a strongly quadrate labrum, all other gyrinid species have a rounded labrum, as well as *Noterus clavicornis*. An emarginate labrum is seen in most of the other dytiscoid species.

15 (3333) Labrum basally. (0) with transverse setose division, ventrad to division lightly colored cuticle present, dorsad to division cuticle darkly colored; (1) entire, no division evident. The labrum of *Angarogyrus, Spanglerogyrus*, and *Heterogyrus* exhibit a unique basal transverse division (fig. S3A and B). All other species studied had the labrum entire.

16 (3334) Labrum dorsally with setae. (0) absent (1) present. The labrum of *Spanglerogyrus*, *Heterogyrus*, and oretochiline species exhibit dorsal setae. These setae are not present in any of the other species studied.

17 (3335) Maxillary galea. (0) two segment; (1) one segmented; (2) absent. The out-group hydradephagan species all have two segmented maxillary galea. Within the Gyrinidae, *Spanglerogyrus* and *Heterogyrus* have two segmented maxillary galea, the gyrinines have maxillary galea with a single segment, and the orectochilines and dineutines have the maxillary galea totally absent (*22, 62, 63*). This character is treated as ordered in the analysis.

18 (3336) Palpi. (0) narrow and elongate; (1) broadened and shortened. Within the Gyrinidae, *Spanglerogyrus* and *Heterogyrus* have narrow and elongate labial and maxillary palpi. The other gyrinines have the palpi broadened and relatively shortened.

19 (3337) Prementum. (0) free, not fused to mentum; (1) fused to mentum. Within the Gyrinidae, *Spanglerogyrus* (*62*) and *Heterogyrus* also have a free prementum, while the remaining gyrinid species have the prementum fused to the mentum.

20 (3338) Mentum. (0) weakly tri-lobed; (1) strongly tri-lobed. *Heterogyrus milloti* and *Cretotortor striatus* have a mentum with a well developed medial lobe, giving the mentum a strongly tri-lobed appearance. All other species studied have a weakly tri-lobed mentum.

21 (3339) Mental lateral lobe. (0) not strongly expanded; (1) strongly expanded. The lateral mental lobes are greatly expanded in the Gyrinidae (*62*).

22 (3340) Clypealium. (0) mostly glabrous, few sparse setae, especially basally; (1) setose with row of long fine setae. The Orectochilini and Dineutini have a strongly setose clypealium. Character not coded for non-gyrinid taxa.

Prothorax

23 (3341) Pronotum with largely expanded medial lobe. (0) absent; (1) present. The pronotum of *Angarogyrus*, *Spanglerogyrus*, *Heterogyrus*, and *Mesogyus* has a strong medial lobe. Other pronotum examined did not exhibit such an expanded medial region to the pronotum.

24 (3342) Expanse of lateral margin of pronotum. (0) not reaching anteriad to medial expanse of pronotum; (1) reaching at least equally anteriad to the medial expanse of pronotum, if not beyond. The lateral margins of the pronotum of *Spanglerogyrus* and *Angarogyrus* do not reach the medial lobe of the pronotum. In *Heterogyrus* and *Mesogyrus* the pronotal lateral margins extend to at least the length of the medial lobe. In the remaining Gyrinidae for which the pronotum is known, the medial lobe of the pronotum is lost and the lateral margins typically extend further anteriad than the medial expanse of the pronotum. Not coded for non-gyrinid taxa.

25 (3343) Pronotal lateral bead. (0) absent; (1) present. Some Hydradephaga have a distinct lateral bead to the pronotum.

26 (3344) Pronotal transverse impressed line. (0) absent; (1) present. Most Gyrinidae have a transverse impressed line following the anterolateral margin of the pronotum (*63*). This line is absent in *Orectochilus* and *Porrorhynchus* species.

27 (3345) Pronotum dorsally with transverse crease. (0) absent; (1) present. Species of *Gyrinus* exhibit a transverse crease dorsally on the pronotum (*22, 64*).

28 (3346) Pronotum with basolateral plicae. (0) absent: (1) present. Species of *Haliplus* exhibit strongly plicae basolaterally on the pronotum (*65*).

29 (3347) Pronotal setation. (0) absent; (1) present. *Spanglerogyrus*, *Heterogyrus*, and the orectochiline species exhibit setation on the pronotum.

30 (3348) Prosternum medially expanded posteriorly and differentiated. (0) Prosternum weakly expanded posteriorly relative to lateral margins, weakly differentiated; (1) Prosternum strongly expanded posteriorly relative to lateral expanse, medially clearly differentiated. In *Spanglerogyrus* the Prosternum is weakly humped, but not strongly ventrally expanded relative to the lateral margins. Other Hydradephagan species have the Prosternum strongly differentiated, either expanded ventrally or modified into a prosternal process.

31 (3349) Prosternal differentiation. (0) not cushion shaped; (1) cushion shaped. In *Heterogyrus*, *Mesogyrus striatus*, and the orectochilines, the Prosternum is medially differentiated into a cushion shape, whose medial region is variously modified.

32 (3350) Prosternal cushion medially. (0) with depression; (1) entire; (2) with elevated process. In *Heterogyrus* and *Mesogyrus striatus* the prosternal cushion has a medial depression. In many orectochilines the prosternal cushion is entire, without a medial depression or elevated process. In some orectochiline species, such as *Orectochilus villosus*, the prosternal cushion has a medial elevated process.

33 (3351) Prosternum medially. (0) without distinct process; (1) with well differentiated process. The genera *Gyrinus* and *Dineutus* have a well differentiated prosternal process as do the out-group hydradephagans studied. Members of *Aulonogyrus* and *Macrogyrus* have neither a well differentiated prosternal process, nor a prosternal cushion. However, their prosterna medially are strongly expanded posteriorly, becoming strongly differentiated from the lateral expanse, different from the situation in *Spanglerogyrus*.

34 (3352) Prosternal process extent. (0) ending at or prior to posterior margin of procoxae; (1) extending just beyond posterior margin of the procoxae; (2) extending between the mesocoxae. The Gyrinidae to not have the prosternal process extending beyond the posterior margin of the procoxae. *Coptoclava longipoda* and *Liadytes longus* have the prosternal process extending just beyond the procoxae (*60*). The remainder of the out-group hydradephagan species have the procoxae extending between the mesocoxae.

35 (3353) Prosternal process form. (0) not strongly raised and plat-form-like, without truncate posterior margin; (1) strongly raised and plat-form-like, posterior margin truncate. The Haliplidae have strongly raised, plat-form-like prosternal process with a truncate posterior margin (*66*).

36 (3354) Pronotum with lateral explanate margin. (0) absent; (1) present. The pronotum of many gyrinid species have a lateral explanat margin to the pronotum.

37 (3355) Pronotum lateral explanate margin color. (0) lightly colored, normally yellow; (1) darkly colored. The lateral explanate margin of most gyrinid species is lightly colored, in some it is darkly colored, as in *Enhydrus* species.

Foreleg

38 (3356) Natatory setae. (0) absent; (1) present. Natatory setae is present on the foreleg of out-group hydradephaga species, with the exception of *Coptoclava longipoda*.

39 (3357) Protibial medial spur number. (0) absent; (1) one spur; (2) two spurs. The species of the subfamily Gyrininae lack protibial spurs. *Heterogyrus* and *Spanglerogyrus* both have a single spur. All the out-group hydradephagans have two medial protibial spurs, with the exception of *Noterus clavicornis*.
40 (3358) Protibial medial spur modification. (0) unmodified; (1) modified for digging; (2) modified raptorially. *Coptoclava longipoda* has the protibial medial spurs modified raptorially (*60*), being elongate and sharp. *Hygrobia* and *Noterus* have the protibial medial spurs modified for digging (*67, 68*). All other species studied with protibial medial spurs unmodified.
41 (3359) Fringe of setae along dorsal and anterior protibial margins. (0) absent; (1) present. *Hygrobia* and *Noterus* have a fringe of short stout setae along the dorsal and anterior apical margins of their protibia (*69*).
42 (3360) Protrochanter ventral face. (0) without series of short stout setae; (1) with series of short stout setae apically; (2) with series of short stout setae extending nearly the entire length of the ventral face. The protrochanters of certain dineutines and orectochilines have a series of short stout setae along their ventral face, either limited apically or extending most the protrochanters length.
43 (3361) Protrochanteric setose patch. (0) absent; (1) present. The protrochanters of *Porrorhynchus* have a distinct setose patch.
44 (3362) Profemoral sub-apicoventral tooth/teeth. (0) absent; (1) present. Certain species of *Dineutus* have a profemoral sub-apicoventral tooth.
45 (3363) Setigerous punctures of anterior face of profemur. (0) absent; (1) present. Most species of gyrinid have at least one or more setigerous punctures present on the anterior face of the profemur (*70*).
46 (3364) Ventral face of profemur. (0) without lines of setae on either anterior or posterior margin; (1) with one line of setae present on posterior margin only; (2) with two lines of setae on the posterior and anterior margin. *Spanglerogyrus* lacks lines of setae of the ventral face of the profemur, *Heterogyrus* has one line of setae along the posterior margin, and nearly all Gyrininae species have either two lines of setae on or at least one.
47 (3365) Setation of ventral face of profemur. (0) not composed of think tufts of setae; (1) composed of thick tufts of setae becoming distally. *Porrorhynchus* species have the two lines of setae of the ventral face of the profemur modified into thick tufts of setae that becoming denser distally.
48 (3366) Setose brush of posterior face of protibia. (0) absent; (1) reduced; (2) fully present. The protibia of most gyrinid species has some sort. In many species the setose brush is reduced to a small patch at the protibial apex, sometimes continue posteriorly by a very narrow strip of setae. The fully present state is a large triangular brush of setae beginning apically on the protibia and continue down the protibia. Most *Dineutus* have a fully present setose brush, as do *Aulonogyrus* species and some *Orectogyrus*. This character is treated as ordered.
49 (3367) Distolateral corner of protibia. (0) not expanded laterally; (1) expanded laterally. The protibia of orectochiline species and those of most dineutines, except *Dineutus* species, have the distolateral corner of the protibia laterally expanded and triangular in form, if not pointed. This character is also exhibited in *Coptoclava longipoda*.

50 (3368) Male protarsomere I posterior face. (0) without recessed pit; (1) with recessed pit containing differently shaped sucker-disc setae. The males of *Macrogyrus* species possess a recessed pit containing differently shaped sucker-disc setae (*71*).

51 (3369) Female protarsomere V posterior face with setae. (0) absent; (1) present but reduced to small patch; (2) present as a line of numerous setae. This character is absent in gyrinines and *Spanglerogyrus*. But appears variously developed within dineutine and orectochilines (*60*). Line of setae is fully present on the posterior face of female protarsomere V in *Heterogyrus*.

Mesoventrite

52 (3370) Modification for proleg reception. (0) absent; (1) present. The mesoventrite of all gyrinids except *Spanglerogyrus* has recessed areas for receiving the prolegs (*22,72*).

53 (3371) Mesoventrite size. (0) smaller than metaventirte; (1) larger than metaventrite. The Gyrinidae have a modified and greatly enlarged metaventrite. The out-group hydradephagan species all have the metaventrite much larger than the mesoventrite.

54 (3372) Mesoventrite with recessed hexagonal area for reception of prosternal process. (0) absent; (1) present. Most of the out-group hydradephagan species have the mesoventrite with a hexagonal recessed area for reception of the prosternal process (*66-68*), with the exception of *Coptoclava longipoda*.

55 (3373) Mesoventrite shape. (0) not triangular; (1) triangular and extensive, often shaped similar to the bow of a ship. All gyrinid species with the exception of *Spanglerogyrus* have the mesoventrite triangular and extensive, shaped similarly to the bow of a ship.

56 (3374) Mesoventral discrimen. (0) absent; (1) present. A medial discrimen of the mesoventirte is present in all Gyrinidae except for *Spanglerogyrus*.

57 (3375) Mesoventrite with paramedical ridges. (0) absent; (1) present. The fossil gyrinid *Baissogyrus savilovi* has distinct paramedical ridges on the mesoventrite.

58 (3376) Mesoventral pit. (0) absent; (1) present. Some *Gyrinus* species have a the mesoventrite basomedially with a distinct pit.

59 (3377) Scutellar shield. (0) visible with elytra close; (1) invisible with elytra closed. The scutellar shield is variously invisible with the elytra closed of the species studied.

60 (3378) Scutellar shield shape. (0) more evenly triangular; (1) transversely triangular. The scutellar shield of *Metagyrinus* is transversely triangular (*73*) and used as a character to distinguish it form *Aulonogyrus*.

61 (3379) Elytral length. (0) not covering abdominal apex; (1) covering abdominal apex. The elytra cover the apex of the abdomen in most of the hydradephagan out-group taxa. The abdominal apex is not covered in the Gyrinidae and in some out-group taxa.

62 (3380) Elytral setation. (0) absent; (1) present but with distinct glabrous regions to the elytra; (2) present, elytra nearly entirely pubescent. Within Gyrinidae the elytra are glabrous in the gyrinines and the dineutines. Pubescence is present on the elytra but with distinct glabrous regions in *Spanglerogyrus*, *Heterogyrus*, *Orectogyrus*, *Patrus*, and most *Gyretes*. Completely pubescent elytra is found in *Orectochilus* and some *Gyretes* like *Gyretes sericeus.*

63 (3381) Elytral explanate lateral margin. (0) absent; (1) present. Many gyrinid species exhibit a broad explanate lateral margin to the elytra.

64 (3382) Elytral explanate lateral margin color. (0) lightly colored, yellow often; (1) darkly colored. Most gyrinids with an explanate latera margin have the margin lightly colored, normally yellow. Rarely is the lateral margin darkly colored, similar in color to the elytral disc, as in *Enhydrus* species.

65 (3383) Ten or more primary punctures accompanied by numerous secondary punctures. (0) absent; (1) present. Species of *Haliplus* have distinctly punctate elytra, with ten or more primary punctures associated with numerous secondary punctures (*65, 66*).

66 (3384) Serial striae number. (0) none evident; (1) nine visible; (2) eleven visible. Within the Gyrinidae the orectochilines have no visible elytral striae, at least dorsally, similarly with *Spanglerogyrus*, *Angarogyrus*, and *Porrorhynchus*. *Heterogyrus*, *Mesogyrus*, *Cretotortor* and most the dineutines, with the exception of *Porrorhynchus,* have nine elytral striae visible. The Gyrinini all have eleven elytral striae.

67 (3385) Elytral strial appearance. (0) punctures; (1) well impressed lines; (2) faintly evident lines. The elytral striae appear as punctures in the gyrinines, as well as in *M.* (*Andogyrus*) *seriatopunctatus* and the fossil *Meiodineutes amurensis*, suggesting that dineutines. Strongly impressed lines are evident in *Heterogyrus*, *Mesogyrus*, *Cretotortor*, *Metagyrinus* and *Enhydrus*. Weakly impressed lines are present primarily in *Dineutus* and *Macrogyrus*. This character is treated as ordered as several *Aulonogyrus* and *Gyrinus* species exhibit intermediate stages between punctate to strongly impressed lines, suggesting a trend from punctures to strongly impressed lines, with weakly impressed lines as a step towards loss of impressed lines and elytral striae in general.

68 (3386) Elytral sutural border. (0) absent; (1) present. The elytra is bordered by an additional, non-serial striae. This border is present in most gyrinid species, being completely lost in dineutines, and lost in some orectochilines.

69 (3387) Elytral lateral plica. (0) absent; (1) present. The two species of *Angarogyrus* studied have a distinct longitudinal plica laterally on the elytra. This character is unique to *Angarogyrus*.

70 (3388) Elytral apices. (0) unmodified; (1) modified. Unmodified elytra, those that are regularly rounded and attenuated towards the apex are common in the out-group hydradephagans studied, but relatively rare in the Gyrinidae. Unmodified elytra are primarily found in *Dineutus* and some *Gyrinus*.

71 (3389) Elytral apex with sutural production. (0) absent; (1) present. The sutural angle of the elytra has a production (*74*) in many species of Gyrinidae. Importantly a sutural production is present in both *Spanglerogyrus* and *Angarogyrus*.

72 (3390) Elytral apex with parasutural production. (0) absent; (1) present. The elytral apex may have a production between the sutural and epipleural angles, the parasutural production. This is present in dineutine species in members of *Dineutus*, *Porrorhynchus*, and *Macrogyrus*.

73 (3391) Elytral apex with epipleural angle modified. (0) absent; (1) present as prominence; (2) present, spinose. The epipleural angle is modified as a strong spine in many orectochilines and some dineutines. The epipleural prominence is variously present among gyrinid species.

74 (3392) Elytral apices with straight truncation. (0) absent; (1) present. In many gyrinid species the elytral apex has a straightly truncate margin. This is common in many orectochilines and dineutines.

75 (3393) Elytral apices with oblique truncation. (0) absent; (1) present. An oblique truncation to the apex of the elytra is less common within Gyrinidae. *Heterogyrus*, *Mesogyrus*, and *Cretotortor* exhibit this type of truncation, as do some oretochilines and very few dineutines.

76 (3394) Elytral apices with serrations/irregularities. (0) absent; (1) present. Some *Dineutus* species exhibit serration and/or irregularities to the elytral apices (*74*).

77 (3395) Elytral apicolateral margins with buzz-saw shaped serration. (0) absent; (1) present. Most species of *Porrorhynchus* exhibit this time of elytral modification. It is also present in *Dineutus micans*.

78 (3396) Elytral with canaliculated microsculpture. (0) absent; (1) present. This microsculpture appears as minute "scratch-like" sculpturing of the elytra. It is present on the elytra of the *Macrogyrus* s. str. species.

Mid-legs

79 (3397) Mid-legs. (0) not broadened nor flattened; (1) not broadened but dorsoventrally flattened with expanded mesotibia; (2) broadened and flattened dorsoventrally; (3) broadened, flattened dorsoventrally, but also shortened and paddle-like. Most of the out-group hydradephagan species have mid-legs that are not broadened or flattened. *Spanglerogyrus* has mid-legs that are not broadened but are dorsoventrally flattened with an expanded mesotibia. The fossil *Angarogyrus mongolicus* has the mid-legs visible, these are interpreted as being similar to those of *Spanglerogyrus* given the majority of other similarities the species share in morphology. The legs appear slightly broader than the totally unmodified legs of the out-group taxa, but certainly not broadened like those of *Coptoclava longipoda* nor of the other gyrinid species. State (2) is unique in the analysis to *Coptoclava longipoda* which exhibits broad and flattened midlegs. State (3) is unique to all Gyrinidae except *Spanglerogyrus* and *Angarogyrus mongolicus*.

80 (3398) Mesocoxal shape. (0) rounded; (1) triangular and strongly transverse. Triangular and strongly transverse mesocoxae are unique to the Gyrinidae (*75*).

81 (3399) Mesocoxae separation (*63*). (0) narrowly separated; (1) broadly separated. The mesocoxae are broadly separated in the dineutines, and most of the orectochiline genera *Patrus* and *Gyretes*.

82 (3400) Meso- and metatibial medial spurs. (0) both spurs large, greater than or nearly equal to half the length of the first tarsomere; (1) both spurs small, less than half the length of the first tarsomere; (2) the metatibia with the posterior spur larger than or nearly equal to half the length of the first metatarsomere; (3) both the meso- and metatibia with the posterior spur larger than or nearly equal to half the first tarsomere. In the out-group hydradephagan species as well as in *Spanglerogyrus* (*62*) and *Heterogyrus* the meso- and metatibial medial spurs are both large. In the majority of the Gyrinidae both spurs are short. In some *Gyrinus* species and orectochiline species the metatibia has the posterior spur large and the anterior spur small. Only in *Macrogyrus howittii* and in one *Gyretes* species was a large posterior spur observed in both the meso- and metatibia.

83 (3401) Mesotarsal claws. (0) not sexually dimorphic; (1) weakly sexually dimorphic; (2) strongly sexually dimorphic. The male mesotarsal claws of *Dineutus* species are strongly sexually dimorphic (*74*). Within *Porrorhynchus* the mesotarsal claws are weakly sexually dimorphic. In the remaining Gyrinidae the mesotarsal claws are not sexually dimorphic.

Metaventrite

84 (3402) Mesoventrite with anteromedial process. (0) absent; (1) present, receiving posterior expansion of prosternal process. Most of the out-group hydradephagan species have the mesoventrite with an anteromedial process that receives the posterior expansion of the prosternal process, with the exception of *Liadytes longus* and *Coptoclava longipoda*[1].

85 (3403) Metaventrite. (0) not largely expanded anteriorly; (1) largely expanded anterior. *Haliplus* species have the metaventrite largely expanded anteriorly (*66*).

86 (3404) Metaventrite paramedially. (0) not constricted; (1) weakly constricted; (2) distinctly constricted. In the out-group hydradephaga species the metaventrite is not noticeably constricted paramedially by the mesocoxae with the exception of *Coptoclava longipoda*, which has a weak constriction. Within the Gyrinidae only *Spanglerogyrus* has the metaventrite weakly constricted paramedially by the mesocoxae, all the remaining Gyrinidae have the metaventrite distinctly constricted, resulting in the formation of "metaventral wings" (*63*).

87 (3405) Metaventral wings. (0) absent; (1) in the form of a near equilateral triangle; (2) narrowed and strap-like. The out-group hydradephagan species and *Spanglerogyrus* lack metaventral wings like other gyrinid species, as per the above character. *Heterogyrus*, *Mesogyrus*, *Mesodineutes*, and the dineutines have the metaventral wings in the form of a near equilateral triangle. The gyrinines and the orectochilines have the metaventral wings strap-like, strongly narrowed medially then gradually broadened laterally.

88 (3406) Medial expanse of metaventrite. (0) relatively narrower and diamond shaped; (1) very broad and pentagonal shaped. The species of *Enhydrus* have a very broad medial expanse of the metaventrite (the area medial to the mesocoxae) that is strongly pentagonal shape with a nearly straight posterior margin. Some *Macrogyrus* species in the subgenus *Andogyrus* come close to have a similarly shaped medial expanse, however, the posterior margin is not nearly as straight.

89 (3407) Discrimen of metaventrite with transverse sulcus. (0) present and long; (1) present but short; (2) absent. Some of the out-group hydradephagan taxa retain a long transverse sulcus associated with the metaventral discrimen, as does *Spanglerogyrus* and interesting many *Macrogyrus* species. An intermediate stage is present in the Gyrinidae, where a transverse sulcus is present but is greatly shortened (*22*). This character is present in *Heterogyrus*, *Mesogyrus antiquus*, and *Baissogyrus*.

90 (3408). Metepisternal ostiole. (0) absent; (1) present. The metepisternum of *Gyrinus* species has an ostiole that is variously developed (*64*).

91 (3409). Metanepisternum shape. (0) largely triangular; (1) lobiform; (2) narrow and trapezoidal in form. The out-group hydradephaga have a largely triangular metanepisternum, as do *Spanglerogyrus*, the gyrinines, and a few *Patrus* species. A lobiform Metanepisternum is found in *Heterogyrus*, *Mesogyrus*, *Mesodineutes*, and the dineutines. A strongly narrowed and trapezoidal shaped Metanepisternum is unique to most of the orectochilines, with the exception of a few *Patrus*.

92 (3410). Metanepisternum reaching coxal cavities. (0) absent, ending prior to coxal cavities; (1) present. This character is found in some of the out-group hydradephagan taxa, but is never present in the Gyrinidae.

93 (3411). Noterid platform. (0) absent; (1) present. The distinct "noterid platform" (*69*) is found only in *Noterus clavicornis*.

Hind legs

94 (3412). Hind legs. (0) narrow; (1) narrow and weakly dorsoventrally flattened with expanded metatibia; (2) broadened for swimming, but not dorsovenrally flattened; (3) broadened and significantly dorsoventrally flattened; (4) broadened, significantly dorsoventrally flattened, and shortened to a paddle-like form. Unmodified narrow hind legs are found in the hydradephagan out-group species of *Hygrobia*, *Haliplus*, and *Liadytes*. Narrow and weakly dorsoventrally flattened hindlegs with expanded metatibia are found in *Spanglerogyrus*, the hind legs of *Angarogyrus mongolicus* are treated similarly as justified for the mid-legs. Hind legs that are broadened for swimming, but not dorsoventrally flattened, are found in the Dytiscidae and *Noterus* species examined. The broadened and significantly dorsoventrally flattened hind-legs, that are not shortened, are found in *Coptoclava longipoda*. Finally the majority of gyrinid species exhibit the paddle-like leg form, with hid legs that are expanded, dorsoventrally flattened, and shortened.

95 (3413). Anterior margin of metacoxae. (0) more transverse; (1) distinctly oblique to very strongly oblique. Most of the hydradephagan out-group species studied (with the exception of the Dytiscidae), *Spanglerogyrus*, the dineutines, and a few orectochilines have a more transverse anterior margin to the metacoxae. In *Heterogyrus*, *Mesogyrus*, the gyrinines, and most of the orectochilines have a distinctly more oblique anterior margin to the metacoxae.

96 (3414). Posterolateral margin of metacoxae. (0) without border; (1) bordered. Many gyrinid species have the posterolateral margin of the metacoxae bordered. *Heterogyrus*, *Mesogyrus*, *Aulonogyrus*, *Orectochilus, Porrorhynchus*, and *Dineutus* do not have a border along the posterolateral margin of the metacoxae.

97 (3415). Metacoxae. (0) not expanded anteriorly; (1) greatly expanded anteriorly. The Dysticidae have the metacoxae greatly expanded anteriorly.

98 (3416). Metacoxal plate (sensu ref [*76*]). (0) present as large plates concealing the basal portion of leg and part of abdomen; (1) present, not concealing leg and abdomen, but continued laterally along anterior margin of metacoxae, (2) strongly reduced present only medially. As ref [*76*] defines metacoxal plates: "the excavation of the metacoxae to form at least weak coxal plates", we consider the medial raised region of the metacoxae to be reduced coxal plates, which in the out-group hydradephagan species studied are present only medially, with the exception of *Haliplus* and *Triaplus* species (*60*), which have large metacoxal plates covering the basal portion of the hind legs (*66*). In the Gyrinidae the metacoxal plates are present medially but also continued laterally along the anterior margin of the metacoxae.

99 (3417). Metacoxal plate secondary reduction in Gyrinidae. (0) absent, metacoxal plate region largely triangular; (1) present, metacoxal plate medially rectangular in form with a narrow anterior bridge or with bridge totally reduced. Within the Gyrinidae the dineutines and *Spanglerogyrus* have large broadly triangular metacoxal plates. Within the orectochilines and gyrinines the metacoxal plates are consricted to a medial rectangular

area, with a narrow anterior bridge continued laterally or with the anterior bridge totally reduced in some taxa. This character is associated with more oblique metacoxae.

100 (3418). Metacoxal process apex. (0) straightly truncate; (1) obliquely truncate; (2) rounded and lobiform. The metacoxal process apex of Gyrinidae is most often obliquely truncate. Many orectochilines have this apex straightly truncate, with many *Orectogyrus* having a rounded or lobiform metacoxal process apex.

Abdomen

101 (3419). Suture of abdominal sternite II. (0) totally or mostly obliterated; (1) present. The species of *Enhydrus* still have the suture of abdominal sternite II present (*61,77*).

102 (3420). Overal shape of abdominal apex. (0) not cylindrical, clearly broadly rounded; (1) distinctly cylindrical and strongly narrowed apically. The orectochilines have a strongly cylindrical and stinctly narrowed abdominal apex.

103 (3421). Abdominal sternites VII & VIII. (0) glabrous; (1) with row of long fine setae. The orectochilines have a row of long fine setae posteromedially on abdominal sternites VII & VIII forming a 'keel' (*22*).

104 (3422). Abdominal sternite VIII. (0) apically longitudinally divided; (1) apically bi-emarginate; (2) entire. This character is discussed in ref [*22*] and treated as ordered in the analysis.

105 (3423). Posterior margin of penultimate abdominal tergite. (0) not trilobed; (1) weakly trilobed; (2) strongly trilobed. This character is discussed in ref [*22*].

106 (3424). Tergite VIII divided medially in half. (0) absent, tergite VIII entire; (1) present. The out-group hydradephagan species have tergite VIII divided medially in half.

107 (3425). Venter coloration. (0) entirely lightly colored; (1) distinctly infuscate; (2) entirely darkly colored. Most gyrinid species have either light (ranging from light red, orange, yellow, and even white) or darkly colored venters (dark reddish brown, brown, to black). Some gyrinine and *Orectogyrus* species exhibit distinctly infuscate venters.

Female reproductive tract

108 (3426). Spermatheca. (0) not largely expanded nor sac-like; (1) largely expanded and sac-like. The spermatheca of dineutines and *Orectochilus* and *Orectogyrus* are largely expanded and sac-like to varying degrees. The spermathecae of other gyrinidea are not nearly as expanded or sac-like.

109 (3427). Spermathecal accessory gland. (0) absent; (1) present. An accessory gland attached to the spermatheca is present in *Spanglerogyrus*, *Heterogyrus*, and the gyrinines.

110 (3428). Bursal accessory gland. (0) absent; (1) present. An accessory gland attached to the bursa is present in *Heterogyrus*, the orectochilines, and *Enhydrus* and *Macrogyrus*.

111 (3429). Vaginal shield. (0) absent; (1) present. The vaginal shield (*78-80*) is present in *Porrorhynchus* and *Dineutus*.

112 (3430). Fertilization duct expansion. (0) absent; (1) weakly expanded; (2) strongly expanded. Most orectochilines have the fertilization duct weakly to strongly expanded. The remaining gyrinid species typically do not have the fertilization duct expanded[7]. This character is treated as ordered in the analysis.

113 (3431). Fertilization duct convolution. (0) absent; (1) randomly convoluted; (2) cork-screw shaped convolution. The fertilizaiton duct of orectochilines is often convoluted with random twists and turns. In certain *Gyretes* species the convolutions are arranged serially into a cork-screw shape (*22*). This character is treated as ordered in the analysis.

114 (3432). Fertilization duct curling. (0) absent: (1) weakly curled; (2) strongly curled. The fertilization duct of *Orectochilus* and *Orectogyrus* species are curled. In *Orectochilus* this curling is weakly, with only a single turn back into itself, similarly with some *Orectogyrus* species. Many *Orectogyrus* species of the *s. str.* subgenus have the fertilization duct strongly curled, with numerous recurves, creating a snail-shell shape. Other gyrinids do not have the fertilization duct curled. This character is treated as ordered in the analysis.

115 (3433). Gonocoxae with medial apodeme. (0) absent; (1) present. This character is described by ref [*22*].

Aedeagus

116 (3434). Orientation of aedeagus in repose. (0) no rotated; (1) rotated. The aedeagus of Gyrinidae is not rotated in repose, unlike other hydradephaga.

117 (3435). Basal piece. (0) distinctly present; (1) present but fused to parameres; (2) absent/indistinguishable. *Spanglerogyrus* has a distinct basal piece (*62*) which was reconfirmed in this study. *Heterogyrus* has a distinct additional sclerite fused to the parameres, which is interpreted here as the basal piece. The remaining Gyrinidae and the out-group hydradephaga species no longer have a distinguishable basal piece. This character is treated as ordered in the analysis.

118 (3436). Paramere position. (0) ventral to median lobe; (1) lateral to median lobe. The genus *Orectogyrus* has parameres that are situated lateral to the median lobe. All other gyrinid psecies have the parameres situated ventral to the medial lobe.

119 (3437). Parameres with ventromedial project. (0) absent; (1) present. The gyrinines have a distinct ventromedial projection extending off the parameres. This projection is in a serially homologous postion to the fused basal piece of *Heterogyrus*. This character is coded here are a distinct character, but it seems reasonable that this character could be interpreted as a transformation series step, supporting the position of the gyrinines as sister to Dineutini + Orectochilini, where this medially project is reduced, representing the total loss of the basal piece.

Sperm

120 (3438). Spermostyle type sperm conjugation. (0) absent; (1) present. The dineutines and *Orectogyrus* and *Orectochilus* have sperm conjugation utilizing a specialized structure, the spermostyle (*81*). This type of sperm conjugation appears associated with female reproductive tracts that have a large sac-like spermatheca.

Supplementary text references

43. Zhang, C. Z. Molecular Clock Dating using MrBayes. https://arxiv.org/pdf/1603.05707.pdf (2016).

44. Zuckerkandl, E. & Pauling, L. In *Horizons in biochemistry* (eds M. Kasha & B. Pullman) 189–249 (Academic Press, New York, NY, 1962).

45. Zuckerkandl, E. & Pauling L. In *Evolving genes and proteins* (eds V. Bryson & H. J. Vogel) 97–166 (Academic Press, New York, NY, 1965).

46. dos Reis, M., Donoghue, P. C. J., & Yang, Z. Bayesian molecular clock dating of species divergences in the genomics era. *Nat. Rev. Genet.* **17**, 71–80 (2015).

47. Donoghue, P. C. J. & Yang, Z. The evolution of methods for establishing evolutionary timescales. *Phil. Trans. R. Soc. B* **371**, 20160020 (2016)

48. Drummond, A. J. , Ho, S. Y. W., Phillips, M. J. & Rambaut, A. Relaxed phylogenetics and dating with confidence. *PLoS Biol.* **4**, e88 (2006).

49. Yang, Z. & Rannala, B. Bayesian estimation of species divergence times under a molecular clock using multiple fossil calibrations with soft bounds. *Mol. Biol. Evol.* **23**, 212–226 (2006).

50. Ho, S. Y. W. Calibrating molecular estimates of substitution rates and divergence times in birds. *J. Avian Biol.* **38**, 409–414 (2007).

51. Ho, S. Y. W. & Phillips, M. J. Accounting for calibration uncertainty in phylogenetic estimation of evolutionary divergence times. *Syst. Biol.* **58**, 367–380 (2009).

52. Pyron, R. A. Divergence time estimation using fossils as terminal taxa and the origins of Lissamphibia. *Syst. Biol.* **60**, 466–481 (2011).

53. Stadler, T. Sampling-through-time in birth–death trees. *J. Theor. Biol.* **267**, 396–404 (2010).

54. Heled, J. & Drummond, A. J. Calibrated tree priors for relaxed phylogenetics and divergence time estimation. *Syst Biol.* **61**(1):138-149 (2012).

55. O'Reilly, J. E. & Donoghue, P. C. J. Tips and nodes are complementary not competing approaches to the calibration of molecular clocks. *Biol. Lett.* **12**: 20150975 (2016). DOI: 10.1098/rsbl.2015.0975

56. Rannala, B. Conceptual issues in Bayesian divergence time estimation *Phil. Trans. R. Soc. B* **371**: 20150134 (2016). DOI: 10.1098/rstb.2015.0134.

57. Arcila, D., Pyron, R. A., Tyler, J. C., Ortí, G. & Betancur-R., R. An evaluation of fossil tip-dating versus node-age calibrations in tetraodontiform fishes (Teleostei: Percomorphaceae). *Mol. Phylogenet. Evol.* **82**, 131–145 (2015).

58. Heath, T. A. Divergence time estimation using Beast v.2.3.2. Dating species divergences with the fossilized birth-death process. Tutorial. http://treethinkers.org/divergence-time-estimation-using-beast/ (2016).

59. Lartillot, N., Phillips, M. J. & Ronquist, F. A mixed relaxed clock model. *Phil. Trans. R. Soc. (B)* **371**, 20150132 (2016).

60. Ponomarenko, A. G. [Mesozoic Coleoptera. Description of new taxa]. *Trudy Paleontologicheskogo Instituta Akademiya Nauk SSSR* **161**, 17–96 (1977).

61. Hatch, M. H. The morphology of Gyrinidae. *Pap. Mich. Acad. Sci., Arts and Letters* **7**, 311–350 (1926).

62. Beutel, R. G. & Roughley, R. E. In *Handbuch der Zoologi/ Handbook of Zoology* Vol. 1: Morphology and Systematics (Archostemata, Adephaga, Myxophaga, Polyphaga partim) (eds R. G. Beutel & R. A. B. Leschen) 55–64 (Walter de Gruyter, inc., Berlin, New York, 2005).

63. Hatch, M. H. The phylogeny and phylogenetic tendencies of Gyrinidae. *Pap. Mich. Acad. Sci., Arts and Letters* **5**, 429–467 (1926).

64. Oygur, S. & Wolfe, G. W. Classification, distribution, and phylogeny of North American north of Mexico species of *Gyrinus* Müller (Coleoptera: Gyrinidae). *B. Am. Mus. Nat. Hist.* **207**, 1–97 (1991).

65. Holmen, H. The aquatic Adephaga (Coleoptera) of Fennoscandia and Denmark. I. Gyrinidae, Haliplidae, Hygrobiidae and Noteridae. *Fauna Entomologica Scandinavica* **20**, 1–168 (1987).

66. van Vondel, B. J. In *Handbuch der Zoologi/ Handbook of Zoology* Vol. 1: Morphology and Systematics (Archostemata, Adephaga, Myxophaga, Polyphaga partim), (eds R. G. Beutel & R. A. B. Leschen) 64–71 (Walter de Gruyter, inc., Berlin, New York, 2005).

67. Dettner, K. In *Handbuch der Zoologi/ Handbook of Zoology* Vol. 1: Morphology and Systematics (Archostemata, Adephaga, Myxophaga, Polyphaga partim) (eds R. G. Beutel & R. A. B. Leschen) 72–79 (Walter de Gruyter, inc., Berlin, New York, 2005).

68. Dettner, K. In *Handbuch der Zoologi/ Handbook of Zoology* Vol. 1: Morphology and Systematics (Archostemata, Adephaga, Myxophaga, Polyphaga partim) (eds R. G. Beutel & R. A. B. Leschen) 85–89 (Walter de Gruyter, inc., Berlin, New York, 2005).

69. Miller, K. B. On the systematics of Noteridae (Coleoptera: Adephaga: Hydradephaga): Phylogeny, description of new tribe, genus and species, and survey of female genital morphology. *Syst. Biodivers.* **7**, 191–214 (2009).

70. Ochs, G. On the West Indian Gyrinidae and a new species of *Gyretes* from northern Brazil. *Am. Mus. Novit.* **125**, 1–8 (1924).

71. Régimbart, M. Essai monographique de la famille des Gyrinidae. 1e partie. *Ann. Soc. Entomol. Fr.* **(6)** 379–458 (1882).

72. Beutel, R. G. Phylogenetic analysis of the family Gyrinidae (Coleoptera) based on mesothoracic and metathoracic characters. *Quaest. Entomol.* **26**, 163–192 (1990).

73. Mazzoldi, P. & Jäch, M. A. In *Water Beetles of China Vol. III* (eds M. A. Jäch & L. Ji) 43–47 (Zoologisch-Botanische Gesellschaft in Österreich and Wiener Coleopterologenverein, 2003).

74. Gustafson, G. T. & Miller, K. B. The New World whirligig beetles of the genus *Dineutus* Macleay, 1825 (Coleoptera, Gyrinidae, Gyrininae, Dineutini). *Zookeys* **476**, 1–135, doi:10.3897/zookeys.476.8630 (2015).

75. Beutel, R. G. & Roughley, R. E. On the systematic postion of the family Gyrinidae (Coleoptera: Adephaga). *Z. Zool. Syst. Evol.* **26**, 380 – 400 (1988).

76. Lawrence, J. F. et al. Phylogeny of the Coleoptera based on morphological characters of adults and larvae. *Ann. Zool.* **61**, 1–217 (2011).

77. Brinck, P. Derivation, taxonomy and history of distribution of whirligig beetle genus *Enhydrus* (Coleoptera - Gyrinidae). *Entomol. Ger.* **4**, 317–326 (1978).

78. Brinck, P. *Porrorhynchus indicans* Walker (Coleoptera: Gyrinidae). A representative of the relict montane forest ecosystem in Sri Lanka. . *P.E.P. Deraniyagala Commemoration Volume (Sri Lanka 1980)*, 103–108 (1980).

79. Brinck, P. A revision of *Rhombodineutus* Ochs in New Guinea (Coleoptera: Gyrinidae). *Entomol. Scand.* **14**, 205–233 (1983).

80. Brinck, P. Evolutionary trends and specific differentiation in *Merodineutus* (Coleoptera: Gyrinidae). *Int. J. Entomol.* **26**, 175–189 (1984).

81. Breland, O. P. & Simmons, E. Preliminary studies of the spermatozoa and the male reproductive system of some whirligig beetles (Coleoptera: Gyrinidae). *Entomol. News* **81**, 101–110 (1970).

Supplementary Figures

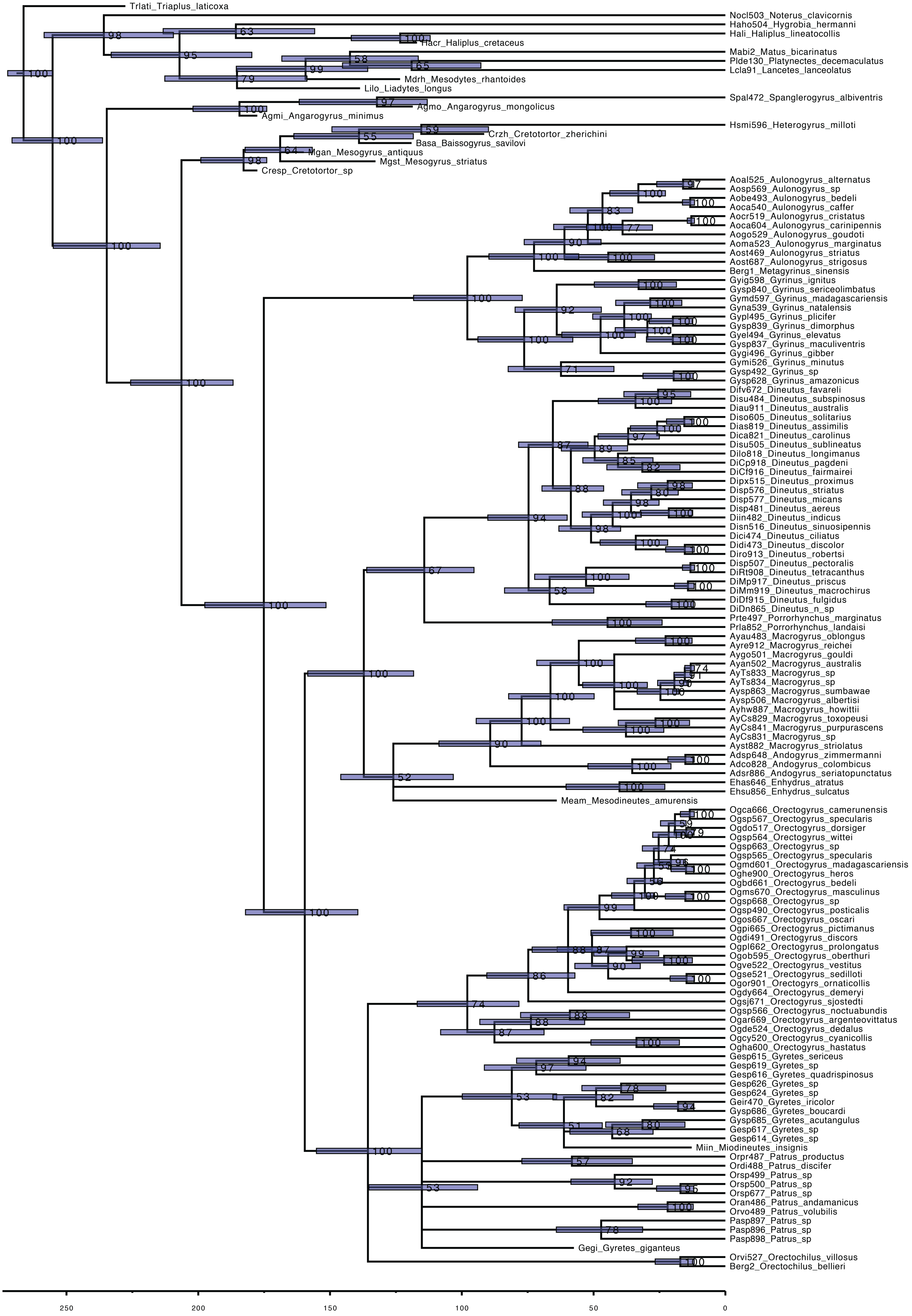

Figure S1: Preferred phylogenetic tree. Bayesian consensus tree resulting from analysis, from which Figure 1 was constructed. Number at nodes indicate posterior probabilities. Bars at nodes denote 95% highest probability density interval for age. Scale in millions of years.

!

Supplementary Tables

Table S1: Character coding for morphology dataset.

Character #	1	5	10	15	20	25	30	35	40
Trlati Triaplus laticoxa	101-	0???-	?000?	?????	0?-0-	?000?	00-00	00-??	????
Nocl503 Noterus clavicornis	101-	0001-	00001	10010	00-0-	10000	10-12	00-11	1100
Haho504 Hygrobia hermanni	000-	0000-	00002	10010	00-0-	00000	10-02	00-12	1100
Mabi2 Matus bicarinatus	101-	0001-	00002	10000	00-0-	10000	10-02	00-12	0000
Plde130 Platynectes decemaculatus	101-	0001-	00002	10010	00-0-	10000	10-02	00-12	0000
Lcla91 Lancetes lanceolatus	101-	0001-	00002	10000	00-0-	10000	10-02	00-12	0000
Hali Haliplus lineatocollis	000-	0001-	00002	100-0	00-0-	10010	10-02	10-12	0000
Hacr Haliplus cretaceus	????	?????	?????	1????	??-0?	?0000	10-12	1????	????
Colg Coptoclava longipoda	011?	?????	000??	1????	00-0-	00000	10-01	00-02	20??
Lilo Liadytes longus	101-	010?-	00002	1??00	00-0-	10000	10-01	00-12	00??
Mdrh Mesodytes rhantoides	101-	0??1-	00002	10???	??-0-	10000	10-12	00-??	????
Spal472 Spanglerogyrus albiventris	1110	11100	01000	01000	01010	11001	00-00	00-01	0000
Hsmi596 Heterogyrus milloti	1111	11100	10001	01000	11011	01001	110-0	01001	0000
Aoal525 Aulonogyrus alternatus	1111	11001	00001	10111	01001	01000	10-00	01000	-000
Aoma523 Aulonogyrus marginatus	????	?????	?????	?????	?????	?????	?????	?????	????
Aocr519 Aulonogyrus cristatus	????	?????	?????	?????	?????	?????	?????	?????	????
Aoca604 Aulonogyrus carinipennis	1111	11001	10001	10111	01001	01000	10-00	01000	-000
Aosp569 Aulonogyrus sp	1111	11001	10001	10111	01001	01000	10-00	01000	-000
Aobe493 Aulonogyrus bedeli	1111	11001	10001	10111	01001	01000	10-00	01100	-000
Aoca540 Aulonogyrus caffer	1111	11001	10001	10111	01001	01000	10-00	00-00	-000
Aost469 Aulonogyrus striatus	1111	11001	00001	10111	01001	01000	10-00	01000	-000
Aogo529 Aulonogyrus goudoti	1111	11001	00001	10111	01001	01000	10-00	00-00	-000
Berg1 Metagyrinus sinensis	1111	1??01	?0001	10???	?1001	??000	?????	?1000	-0??
Gyig598 Gyrinus ignitus	1111	11001	10001	10111	01001	01100	10-10	00-00	-000
Aost687 Aulonogyrus strigosus	1111	11001	10001	10111	01001	01000	10-00	01000	-000
Gymi526 Gyrinus minutus	1111	11101	00001	10111	01001	01100	10-00	00-00	-000

Gymd597 Gyrinus madagascariensis	1111	11001	10001	10111	01001	01100	10-10	00-00	-000
Gyna539 Gyrinus natalensis	1111	11001	10001	10111	01001	01100	10-10	00-00	-000
Gypl495 Gyrinus plicifer	1111	11001	10001	10111	01001	01100	10-10	00-00	-000
Gygi496 Gyrinus gibber	1111	11001	10001	10111	01001	01100	10-10	00-00	-000
Gyel494 Gyrinus elevatus	1111	11001	10001	10111	01001	01100	10-10	00-00	-000
Gysp492 Gyrinus sp	1111	11001	00001	10111	01001	01100	10-10	00-00	-000
Gysp628 Gyrinus amazonicus	1111	11001	00001	10111	01001	01100	10-10	00-00	-000
Difv672 Dineutus fauveli	1111	14001	00001	10211	01101	01000	10-10	00-00	-010
Disp507 Dineutus pectoralis	1111	14001	00001	10211	01101	01000	10-10	00-00	-000
Disu484 Dineutus subspinosus	1111	14001	00001	10211	01101	01000	10-10	00-00	-010
Diso605 Dineutus solitarius	1111	14001	00001	10211	01101	01000	10-10	00-00	-010
Dipx515 Dineutus proximus	1111	14001	00001	10211	01101	01000	10-10	00-00	-010
Disp576 Dineutus striatus	1111	14001	00001	10211	01101	01000	10-10	00-00	-010
Dici474 Dineutus ciliatus	1111	14001	00001	10211	01101	01000	10-10	00-00	-010
Didi473 Dineutus discolor	1111	14001	00001	10211	01101	01000	10-10	00-00	-010
Disn516 Dineutus sinuosipennis	1111	14001	00001	10211	01101	01000	10-10	00-00	-010
Disp481 Dineutus aereus	1111	14001	00001	10211	01101	01000	10-10	00-00	-010
Disu505 Dineutus sublineatus	1111	14001	00001	10211	01101	01000	10-10	00-00	-010
Diin482 Dineutus indicus	1111	14001	00001	10211	01101	01000	10-10	00-00	-010
Disp577 Dineutus micans	1111	14001	00001	10211	01101	01000	10-10	00-00	-010
Ayau483 Macrogyrus oblongus	1111	11000	10001	10211	01101	01000	10-00	00-00	-000
Aygo501 Macrogyrus gouldi	1111	11000	10001	10211	01101	01000	10-00	01000	-000
Ayan502 Macrogyrus australis	1111	11000	10001	10211	01101	01000	10-00	00-00	-000
Aysp506 Macrogyrus albertisi	1111	11000	10001	10211	01101	01000	10-10	00-00	-000
Ehas646 Enhydrus atratus	1111	13000	10001	10211	01101	01000	10-10	01100	-000
Adsp648 Andogyrus zimmermanni	1111	11000	10001	10211	01101	01000	10-00	00-00	-000
Prte497 Porrorhynchus marginatus	1111	14000	00011	10211	01101	00000	10-10	01000	-001
Ogca666 Orectogyrus camerunensis	1111	14000	-0211	11211	01101	01001	111-0	01000	-020
Gesp615 Gyretes sericeus	1111	11000	10001	11211	01101	01002	112-0	00-00	-000

Ogsp566 Orectogyrus noctuabundis	1111	14000	-0201	11211	01101	01001	111-0	01000	-000
Orvi527 Orectochilus villosus	1111	11000	10011	11211	01101	00002	112-0	00-00	-000
Berg2 Orectochilus bellieri	1111	11000	10011	11211	01101	00002	112-0	00-00	-000
Ogcy520 Orectogyrus cyanicollis	1111	14000	-0211	11211	01101	01001	111-0	01000	-020
Ogha600 Orectogyrus hastatus	1111	14000	-0211	11211	01101	01001	111-0	01000	-000
Orpr487 Patrus productus	1111	14000	-0101	11211	01101	01001	111-0	01000	-000
Ordi488 Patrus discifer	1111	14001	10001	11211	01101	01001	111-0	01000	-000
Orsp499 Patrus sp	1111	14000	-0101	11211	01101	00001	112-0	00-00	-020
Oran486 Patrus andamanicus	1111	14001	-0101	11211	01101	01001	111-0	01000	-010
Orvo489 Patrus volubilis	1111	14001	-0101	11211	01101	01001	111-0	01000	-010
Orsp500 Patrus sp	1111	14001	-0101	11211	01101	00001	111-0	00-00	-020
Orsp677 Patrus sp	1111	14000	-0101	11211	01101	00001	111-0	00-00	-020
Ogar669 Orectogyrus argenteovittatus	1111	14000	-0201	11211	01101	01001	111-0	01000	-000
Ogsj671 Orectogyrus sjostedti	1111	14001	-0211	11211	01101	01001	112-0	01000	-020
Ogpi665 Orectogyrus pictimanus	1111	14000	-0101	11211	01101	01001	111-0	01000	-020
Ogde524 Orectogyrus dedalus	1111	14000	-0211	11211	01101	01001	111-0	01000	-000
Ogdi491 Orectogyrus discors	1111	14000	-0101	11211	01101	01001	111-0	01000	-020
Ogos667 Orectogyrus oscari	1111	14000	-0211	11211	01101	01001	111-0	01000	-020
Ogpl662 Orectogyrus prolongatus	1111	14001	-0211	11211	01101	01001	111-0	01000	-010
Ogdy664 Orectogyrus demeryi	1111	14000	-0101	11211	01101	01001	111-0	01000	-020
Ogsp565 Orectogyrus specularis	1111	14000	-0211	11211	01101	01001	111-0	01000	-020
Ogms670 Orectogyrus masculinus	1111	14000	-0211	11211	01101	01001	111-0	01000	-020
Ogsp663 Orectogyrus sp	1111	14000	-0211	11211	01101	01001	111-0	01000	-020
Ogmd601 Orectogyrus madagascariensis	1111	14000	-0211	11211	01101	01001	111-0	01000	-010
Ogdo517 Orectogyrus dorsiger	1111	14000	-0211	11211	01101	01001	111-0	01000	-020
Ogsp490 Orectogyrus posticalis	????	?????	?????	?????	?????	?????	?????	?????	????
Ogsp564 Orectogyrus wittei	1111	14000	-0211	11211	01101	01001	111-0	01000	-020
Ogbd661 Orectogyrus bedeli	1111	14000	-0201	11211	01101	01001	111-0	01000	-020
Ogsp567 Orectogyrus specularis	1111	14000	-0211	11211	01101	01001	111-0	01000	-020

Ogsp668 Orectogyrus sp	????	?????	?????	?????	?????	?????	?????	?????	????
Ogob595 Orectogyrus oberthuri	1111	14000	-0211	11211	01101	01001	111-0	01000	-020
Ogse521 Orectogyrus sedilloti	1111	14000	-0201	11211	01101	01001	111-0	01000	-000
Ogve522 Orectogyrus vestitus	1111	14000	-0211	11211	01101	01001	111-0	01000	-020
Gesp626 Gyretes sp	1111	11001	-0101	11211	01101	01001	112-0	01000	-010
Gesp619 Gyretes sp	1111	11001	-0101	11211	01101	01001	111-0	01000	-010
Gesp616 Gyretes quadrispinosus	1111	11001	-0101	11211	01101	01001	112-0	01000	-010
Gesp624 Gyretes sp	1111	11001	-0101	11211	01101	01001	111-0	00-00	-010
Geir470 Gyretes iricolor	1111	11001	-0101	11211	01101	01001	111-0	00-00	-010
Gysp686 Gyretes boucardi	1111	11001	-0101	11211	01101	01001	111-0	00-00	-010
Gysp685 Gyretes acutangulus	1111	11001	-0101	11211	01101	01001	112-0	00-00	-010
Gesp617 Gyretes sp	1111	11001	-0101	11211	01101	01001	112-0	00-00	-010
Gesp614 Gyretes sp	1111	11001	-0101	11211	01101	01001	112-0	00-00	-010
Ayhw887 Macrogyrus howittii	1111	11000	10001	10211	01101	01000	10-00	00-00	-000
Ayre912 Macrogyrus reichei	1111	11000	10001	10211	01101	01000	10-00	00-00	-000
Ayst882 Macrogyrus striolatus	1111	11000	10001	10211	01101	01000	10-00	00-00	-000
Adsr886 Andogyrus seriatopunctatus	1111	11000	10001	10211	01101	01000	10-00	00-00	-010
Adco828 Andogyrus colombicus	1111	11000	10001	10211	01101	01000	10-00	00-00	-000
AyCs829 Macrogyrus toxopeusi	1111	11001	10001	10211	01101	01000	10-00	00-00	-000
AyCs841 Macrogyrus purpurascens	1111	11001	10001	10211	01101	01000	10-00	00-00	-000
Aysp863 Macrogyrus sumbawae	1111	11000	10001	10211	01101	01000	10-00	00-00	-000
AyTs834 Macrogyrus sp	1111	11000	10001	10211	01101	01000	10-00	00-00	-000
AyTs833 Macrogyrus sp	1111	11000	10001	10211	01101	01000	10-00	00-00	-000
AyCs831 Macrogyrus sp	1111	11001	10001	10211	01101	01000	10-00	00-00	-000
DiDf915 Dineutus fulgidus	1111	14001	00001	10211	01101	01000	10-10	00-00	-000
DiDn865 Dineutus n sp	1111	14001	00001	10211	01101	01000	10-10	00-00	-000
DiRt908 Dineutus tetracanthus	1111	14001	00001	10211	01101	01000	10-10	00-00	-000
Dilo818 Dineutus longimanus	1111	14001	00001	10211	01101	01000	10-10	00-00	-010
DiCp918 Dineutus pagdeni	1111	14001	00001	10211	01101	01000	10-10	00-00	-010
Dica821 Dineutus carolinus	1111	14001	00001	10211	01101	01000	10-10	00-00	-010
Dias819 Dineutus assimilis	1111	14001	00001	10211	01101	01000	10-10	00-00	-010

Diro913 Dineutus robertsi	1111	14001	00001	10211	01101	01000	10-10	00-00	-010
Prla852 Porrorhynchus landaisi	1111	12000	00011	10211	01101	00000	10-10	01000	-001
Ehsu856 Enhydrus sulcatus	1111	13000	10001	10211	01101	01000	10-00	01100	-000
DiCf916 Dineutus fairmairei	1111	14001	00001	10211	01101	01000	10-10	00-00	-010
Diau911 Dineutus australis	1111	14001	00001	10211	01101	01000	10-10	00-00	-010
DiMp917 Dineutus priscus	1111	14001	00001	10211	01101	01000	10-10	00-00	-000
DiMm919 Dineutus macrochirus	1111	13001	00001	10211	01101	01000	10-10	00-00	-000
Ogor901 Orectogyrs ornaticollis	1111	14000	-0201	11211	01101	01001	111-0	01000	-000
Oghe900 Orectogyrus heros	1111	14000	-0211	11211	01101	01001	111-0	01000	-020
Pasp897 Patrus sp	1111	14000	-0101	11211	01101	00001	111-0	01000	-020
Pasp896 Patrus sp	1111	14000	-0101	11211	01101	00001	111-0	01000	-000
Pasp898 Patrus sp	1111	14000	-0101	11211	01101	00001	111-0	01000	-010
Gysp840 Gyrinus sericeolimbatus	1111	11001	10001	10111	01001	01100	10-10	00-00	-000
Gysp839 Gyrinus dimorphus	1111	11001	10001	10111	01001	01100	10-10	00-00	-000
Gysp837 Gyrinus maculiventris	1111	11001	10001	10111	01001	01100	10-10	00-00	?000
Agmo Angarogyrus mongolicus	111?	?????	01000	0????	???10	?????	?????	?????	????
Agmi Angarogyrus minimus	111?	?????	?1?00	?????	?????	?????	?????	?????	????
Basa Baissogyrus savilovi	????	?????	?????	?????	?????	?????	?????	?????	????
Mgan Mesogyrus antiquus	111?	?????	?0?01	?????	???11	0100?	?????	?????	????
Mgst Mesogyrus striatus	111?	?????	???01	?????	11???	?????	110-0	0????	????
Crzh Cretotortor zherichini	111?	?????	???01	?????	???11	?????	?????	?????	????
Meam Mesodineutes amurensis	????	?????	?????	?????	?????	?????	?????	?????	????
Gegi Gyretes giganteus	1111	???00	-0101	11???	???01	?????	?????	?????	????
Miin Miodineutes insignis	111?	?????	?0?01	11???	???01	?????	?????	?????	????
Cresp Cretotortor sp	111?	?????	10001	?????	?1?1?	0?00?	110-0	0????	????

Character #	**44**	**50**	**55**	**60**	**65**	**70**	**75**	**80**	**85**
	**|**	**|**	**|**	**|**	**|**	**|**	**|**	**|**	**|**
Trlati Triaplus laticoxa	??????	??000	0-000	0??0-	00-10	00000	00000	0-??0	00
Nocl503 Noterus clavicornis	000-00	00001	0-001	-100-	00-00	00000	00000	0-001	00
Haho504 Hygrobia hermanni	000-00	00001	0-000	0000-	00-00	00000	00000	0-001	00

Mabi2 Matus bicarinatus	000-00	00001	0-000	0100-	00-00	00000	00000	0-001	00
Plde130 Platynectes decemaculatus	000-00	00001	0-000	0100-	00-00	00000	00000	0-001	00
Lcla91 Lancetes lanceolatus	000-00	00001	0-000	0000-	00-00	10001	00000	0-001	00
Hali Haliplus lineatocollis	000-00	00001	0-001	-100-	1--00	00000	00000	0-001	10
Hacr Haliplus cretaceus	??????	???00	????1	-100-	1--00	00000	0000?	0-??0	10
Colg Coptoclava longipoda	0????1	??000	00000	0100-	00-00	00000	00002	0-000	01
Lilo Liadytes longus	0????0	0?00?	0?0?0	0100-	00-00	?????	????0	0-0?0	00
Mdrh Mesodytes rhantoides	0?????	??001	0-000	0000-	00-00	10001	0000?	0-??1	00
Spal472 Spanglerogyrus albiventris	000-00	00010	00000	0010-	00-10	11000	00001	10000	01
Hsmi596 Heterogyrus milloti	011010	02110	11000	00110	01110	10010	10003	10000	02
Aoal525 Aulonogyrus alternatus	012020	0?110	11000	00010	02010	10001	00003	10100	02
Aoma523 Aulonogyrus marginatus	??????	?????	?????	?????	?????	?????	?????	?????	??
Aocr519 Aulonogyrus cristatus	??????	?????	?????	?????	?????	?????	?????	?????	??
Aoca604 Aulonogyrus carinipennis	012020	00110	11000	00010	02110	10001	00003	10100	02
Aosp569 Aulonogyrus sp	012020	0?110	11000	00010	02010	10001	00003	10100	02
Aobe493 Aulonogyrus bedeli	012010	00110	11000	00011	02010	10001	00003	10100	02
Aoca540 Aulonogyrus caffer	012020	00110	11000	0000-	02010	10001	00003	10100	02
Aost469 Aulonogyrus striatus	012020	00110	11000	00010	02010	10001	00003	10100	02
Aogo529 Aulonogyrus goudoti	012020	00110	11000	0000-	02110	10001	00003	10100	02
Berg1 Metagyrinus sinensis	?????0	??110	1?0?0	10010	021?0	10001	00003	1?10?	?2
Gyig598 Gyrinus ignitus	012010	00110	11010	0000-	02010	10001	00003	10100	02
Aost687 Aulonogyrus strigosus	012020	00110	11000	00010	02010	10001	00003	10100	02
Gymi526 Gyrinus minutus	012010	00110	11000	0000-	02010	10001	00003	10100	02
Gymd597 Gyrinus madagascariensis	012010	00110	11010	0000-	02010	00000	00003	10100	02
Gyna539 Gyrinus natalensis	012010	00110	11000	0000-	02010	10001	00003	10100	02
Gypl495 Gyrinus plicifer	012010	00110	11010	0000-	02010	00000	00003	10100	02
Gygi496 Gyrinus gibber	012010	00110	11010	0000-	02010	00000	00003	10100	02
Gyel494 Gyrinus elevatus	012010	00110	11010	0000-	02010	00000	00003	10100	02
Gysp492 Gyrinus sp	012010	00110	11000	0000-	02010	10011	00003	10200	02
Gysp628 Gyrinus amazonicus	012010	00110	11000	0000-	02010	00000	00003	10200	02

Difv672 Dineutus favareli	012010	02110	11001	-000-	01200	11120	01003	11120	02
Disp507 Dineutus pectoralis	011020	02110	11001	-000-	01200	10010	10003	11120	02
Disu484 Dineutus subspinosus	012010	02110	11001	-000-	01200	10020	11003	11120	02
Diso605 Dineutus solitarius	112020	00110	11001	-000-	01200	00000	00003	11120	02
Dipx515 Dineutus proximus	012020	00110	11001	-000-	01200	00000	00003	11120	02
Disp576 Dineutus striatus	012020	00110	11001	-000-	01200	11021	01003	11120	02
Dici474 Dineutus ciliatus	012020	00110	11001	-000-	01200	00000	00003	11120	02
Didi473 Dineutus discolor	112020	00110	11001	-000-	01200	11000	00003	11120	02
Disn516 Dineutus sinuosipennis	012020	00110	11001	-000-	01200	11100	00003	11120	02
Disp481 Dineutus aereus	112020	00110	11001	-000-	01200	11000	01003	11120	02
Disu505 Dineutus sublineatus	112020	00110	11001	-000-	01200	00000	00003	11120	02
Diin482 Dineutus indicus	112020	00110	11001	-000-	01200	00000	00003	11120	02
Disp577 Dineutus micans	012020	00110	11001	-000-	01200	10000	01103	11120	02
Ayau483 Macrogyrus oblongus	011021	12110	11000	00011	01200	11110	00013	11100	02
Aygo501 Macrogyrus gouldi	011011	12110	11000	00010	01210	11120	00013	11100	02
Ayan502 Macrogyrus australis	011011	11110	11000	0000-	01200	11120	00013	11100	02
Aysp506 Macrogyrus albertisi	011011	12110	11000	00010	01200	11120	00013	11100	02
Ehas646 Enhydrus atratus	001001	00110	11000	00010	01100	10001	00003	11100	02
Adsp648 Andogyrus zimmermanni	012001	10110	11000	0000-	00-00	00000	00003	11100	02
Prte497 Porrorhynchus marginatus	002121	02110	11001	-0010	01200	11110	01103	11110	02
Ogca666 Orectogyrus camerunensis	011021	02110	11000	00110	00-10	11021	00003	10100	02
Gesp615 Gyretes sericeus	011001	00110	11001	-020-	00-00	10020	10003	11100	02
Ogsp566 Orectogyrus noctuabundis	001001	00110	11000	00110	00-00	10001	00003	10100	02
Orvi527 Orectochilus villosus	001001	00110	11000	0020-	00-10	00000	00003	10100	02
Berg2 Orectochilus bellieri	001001	00110	11000	0020-	00-10	00000	00003	10100	02
Ogcy520 Orectogyrus cyanicollis	011021	02110	11000	00110	00-10	10010	00003	10100	02
Ogha600 Orectogyrus hastatus	011021	01110	11000	00110	00-10	10011	00003	10100	02

Orpr487 Patrus productus	011001	01110	11001	-0110	00-00	10021	00003	11100	02
Ordi488 Patrus discifer	011001	00110	11000	00110	00-00	10011	00003	11200	02
Orsp499 Patrus sp	001011	02110	11000	00111	00-00	10010	10003	11200	02
Oran486 Patrus andamanicus	011001	00110	11001	-0110	00-00	10021	00003	11100	02
Orvo489 Patrus volubilis	011001	00110	11000	00110	00-00	10021	00003	11100	02
Orsp500 Patrus sp	011001	00110	11000	00111	00-00	10011	00003	11200	02
Orsp677 Patrus sp	011011	01110	11000	00110	00-00	10001	00003	11200	02
Ogar669 Orectogyrus argenteovittatus	001011	00110	11000	00110	00-00	10000	10003	10100	02
Ogsj671 Orectogyrus sjostedti	011021	02110	11000	00110	00-10	10011	00003	10100	02
Ogpi665 Orectogyrus pictimanus	011011	02110	11000	00110	00-00	10010	10003	10100	02
Ogde524 Orectogyrus dedalus	001011	00110	11000	00110	00-00	111-0	00003	10100	02
Ogdi491 Orectogyrus discors	001011	01110	11000	00110	00-00	10010	10003	10100	02
Ogos667 Orectogyrus oscari	001001	01110	11000	00110	00-00	10020	00003	10100	02
Ogpl662 Orectogyrus prolongatus	001011	0?110	11000	00110	00-10	10011	00003	10100	02
Ogdy664 Orectogyrus demeryi	011011	01110	11000	00110	00-00	00000	00003	10100	02
Ogsp565 Orectogyrus specularis	011021	02110	11000	00110	00-10	10021	00003	10100	02
Ogms670 Orectogyrus masculinus	011011	02110	11000	00110	00-10	10021	00003	10100	02
Ogsp663 Orectogyrus sp	011021	0?110	11000	00110	00-10	10021	00003	10100	02
Ogmd601 Orectogyrus madagascariensis	011021	02110	11000	00110	00-10	10021	00003	10100	02
Ogdo517 Orectogyrus dorsiger	011021	02110	11000	00110	00-10	10020	00003	10100	02
Ogsp490 Orectogyrus posticalis	??????	?????	?????	?????	?????	?????	?????	?????	??
Ogsp564 Orectogyrus wittei	011021	02110	11000	00110	00-10	10021	00003	10100	02
Ogbd661 Orectogyrus bedeli	011021	02110	11000	00110	00-10	10021	00003	10100	02
Ogsp567 Orectogyrus specularis	011021	02110	11000	00110	00-10	10021	00003	10100	02
Ogsp668 Orectogyrus sp	??????	?????	?????	?????	?????	?????	?????	?????	??
Ogob595 Orectogyrus oberthuri	001021	02110	11000	00110	00-10	10011	00003	10100	02
Ogse521 Orectogyrus sedilloti	001011	01110	11000	00110	00-10	10011	00003	10100	02
Ogve522 Orectogyrus vestitus	001021	0?110	11000	00110	00-10	10011	00003	10100	02
Gesp626 Gyretes sp	011001	00110	11001	-0110	00-00	10011	00003	11300	02
Gesp619 Gyretes sp	011001	0?110	11001	-0110	00-00	10011	00003	11100	02

Gesp616 Gyretes quadrispinosus	011001	02110	11001	-0110	00-00	11020	00003	11200	02
Gesp624 Gyretes sp	011001	00110	11001	-010-	00-00	00000	00003	11100	02
Geir470 Gyretes iricolor	011001	00110	11001	-010-	00-10	00000	00003	11100	02
Gysp686 Gyretes boucardi	011001	00110	11001	-010-	00-00	10001	00003	11100	02
Gysp685 Gyretes acutangulus	011001	00110	11001	-010-	00-00	10001	00003	11200	02
Gesp617 Gyretes sp	011001	00110	11001	-010-	00-00	10001	00003	11200	02
Gesp614 Gyretes sp	011001	00110	11001	-010-	00-00	10001	00003	11200	02
Ayhw887 Macrogyrus howittii	012001	11110	11000	0000-	01200	10011	00013	11300	02
Ayre912 Macrogyrus reichei	001021	11110	11000	00011	01200	10010	01013	11100	02
Ayst882 Macrogyrus striolatus	011011	11110	11000	00011	01200	10011	00013	11100	02
Adsr886 Andogyrus seriatopunctatus	012001	10110	11000	0000-	01000	10011	00003	11100	02
Adco828 Andogyrus colombicus	012001	10110	11000	0000-	01200	00000	00003	11100	02
AyCs829 Macrogyrus toxopeusi	011001	10110	11000	0000-	01200	11110	00003	11100	02
AyCs841 Macrogyrus purpurascens	012001	1?110	11000	0000-	01200	11110	00003	11200	02
Aysp863 Macrogyrus sumbawae	011011	11110	11000	0000-	01200	11011	00013	11100	02
AyTs834 Macrogyrus sp	011011	1?110	11000	00011	01200	10110	00013	11100	02
AyTs833 Macrogyrus sp	011011	1?110	11000	00011	01200	11110	00013	11200	02
AyCs831 Macrogyrus sp	012001	10110	11000	0000-	01200	11110	00003	11200	02
DiDf915 Dineutus fulgidus	111020	02110	11001	-000-	01200	00000	00003	11120	02
DiDn865 Dineutus n sp	111020	02110	11001	-000-	01200	00000	00003	11120	02
DiRt908 Dineutus tetracanthus	011020	02110	11001	-000-	01200	10120	00003	11120	02
Dilo818 Dineutus longimanus	111020	00110	11001	-000-	01200	11100	01003	11120	02
DiCp918 Dineutus pagdeni	111020	01110	11001	-000-	01200	00000	00003	11120	02
Dica821 Dineutus carolinus	112020	00110	11001	-000-	01200	10000	01003	11120	02
Dias819 Dineutus assimilis	012020	00110	11001	-000-	01200	11000	00003	11120	02
Diro913 Dineutus robertsi	012020	00110	11001	-000-	01200	00000	00003	11120	02
Prla852 Porrorhynchus landaisi	002121	02110	11001	-0010	00-00	11120	00103	11110	02
Ehsu856 Enhydrus sulcatus	000011	00110	11000	00011	01100	00000	00003	11100	02
DiCf916 Dineutus fairmairei	011020	01110	11001	-000-	01200	00000	00003	11120	02
Diau911 Dineutus australis	012020	02110	11001	-000-	01200	10011	01003	11120	02

DiMp917 Dineutus priscus	011020	02110	11001	-000-	01200	00000	00003	11120	02
DiMm919 Dineutus macrochirus	011020	02110	11001	-000-	01200	00000	00003	11120	02
Ogor901 Orectogyrs ornaticollis	011011	0?110	11000	00110	00-10	10011	00003	10100	02
Oghe900 Orectogyrus heros	011021	02110	11000	00110	00-10	10010	10003	10100	02
Pasp897 Patrus sp	001001	0?110	11000	00110	00-00	10001	00003	10100	02
Pasp896 Patrus sp	001001	00110	11000	00110	00-00	10001	00003	10100	02
Pasp898 Patrus sp	001001	0?110	11000	00110	00-00	10021	00003	10100	02
Gysp840 Gyrinus sericeolimbatus	012010	00110	11010	0000-	02010	10001	00003	10100	02
Gysp839 Gyrinus dimorphus	012010	00110	11010	0000-	02010	10001	00003	10200	02
Gysp837 Gyrinus maculiventris	012010	00110	11010	0000-	02010	00000	00003	10100	02
Agmo Angarogyrus mongolicus	??????	?????	????0	00?0-	00-11	11000	00001	?????	??
Agmi Angarogyrus minimus	??????	?????	?????	??10-	00-11	11000	0000?	?????	??
Basa Baissogyrus savilovi	??????	??110	1110?	?????	?????	?????	?????	10??0	02
Mgan Mesogyrus antiquus	??????	??110	11?00	00?10	01110	10010	1000?	10??0	02
Mgst Mesogyrus striatus	??????	?????	?????	?????	?????	1???1	0????	10??0	02
Crzh Cretotortor zherichini	??????	?????	????0	0??10	01110	10000	1000?	10???	??
Meam Mesodineutes amurensis	??????	??110	11???	?0?0-	01000	00000	0000?	11??0	02
Gegi Gyretes giganteus	??????	?????	????0	00?10	00-10	10011	0000?	?????	??
Miin Miodineutes insignis	??????	?????	?????	?????	00-?0	10001	0000?	?????	??
Cresp Cretotortor sp	??????	?????	????0	00?10	01010	10010	1000?	?????	??

	87 |	95 |	100 |	105 |	110 |	115 |	120 |
Trlati Triaplus laticoxa	0-000100	0?00-	-10?2	0????	?????	?????	?
Nocl503 Noterus clavicornis	0-000112	0002-	-0002	0?0--	-----	-12-_	-
Haho504 Hygrobia hermanni	0-000000	0002-	-0002	011--	-----	-12--	-
Mabi2 Matus bicarinatus	0-200102	1012-	-0002	010--	-----	-12--	-
Plde130 Platynectes decemaculatus	0-200102	1012-	-0002	012--	-----	-12--	-
Lcla91 Lancetes lanceolatus	0-200102	1012-	-0002	010--	-----	-12--	-
Hali Haliplus lineatocollis	0-000000	0000-	-0002	010--	-----	-12--	-
Hacr Haliplus cretaceus	0-0?0000	0000-	-?002	01???	?????	?????	?
Colg Coptoclava longipoda	00200003	0002-	-0002	01???	?????	?????	?

Lilo Liadytes longus	0-000100	0012-	?????	?????	?????	?12--	?
Mdrh Mesodytes rhantoides	0-200100	1012?	??0?2	01???	?????	?12--	?
Spal472 Spanglerogyrus albiventris	00000001	01010	10000	00001	00000	00000	0
Hsmi596 Heterogyrus milloti	10101004	00011	10001	00001	10000	00100	0
Aoal525 Aulonogyrus alternatus	20200004	10011	10002	20101	00000	00201	?
Aoma523 Aulonogyrus marginatus	????????	?????	?????	?????	?????	?????	?
Aocr519 Aulonogyrus cristatus	????????	?????	?????	?????	?????	?????	?
Aoca604 Aulonogyrus carinipennis	20200004	10011	10002	20001	00000	00201	?
Aosp569 Aulonogyrus sp	20200004	10011	10002	20001	00000	00201	?
Aobe493 Aulonogyrus bedeli	20200004	10011	10002	20101	00000	00201	?
Aoca540 Aulonogyrus caffer	20200004	10011	10002	20101	00000	00201	?
Aost469 Aulonogyrus striatus	20200004	10011	10002	20201	00000	00201	?
Aogo529 Aulonogyrus goudoti	20200004	11011	10002	20201	00000	00201	0
Berg1 Metagyrinus sinensis	??????04	1?0??	??002	10?01	00000	0020?	?
Gyig598 Gyrinus ignitus	20210004	11011	00002	00201	00000	00201	0
Aost687 Aulonogyrus strigosus	20200004	11011	10002	20201	00000	00201	0
Gymi526 Gyrinus minutus	20200004	11011	10002	00101	00000	00201	?
Gymd597 Gyrinus madagascariensis	20210004	11011	00002	00201	00000	00201	0
Gyna539 Gyrinus natalensis	20210004	10011	00002	00201	00000	00201	?
Gypl495 Gyrinus plicifer	20210004	11011	10002	00201	00000	00201	0
Gygi496 Gyrinus gibber	20210004	11011	10002	00201	00000	00201	0
Gyel494 Gyrinus elevatus	20210004	11011	10002	00201	00000	00201	?
Gysp492 Gyrinus sp	20200004	11011	10002	00001	00000	00201	?
Gysp628 Gyrinus amazonicus	20200004	11011	00002	00001	00000	00201	?
Difv672 Dineutus favareli	10201004	00010	10002	00010	01000	10200	?
Disp507 Dineutus pectoralis	10201004	00010	10002	00110	01000	10200	?
Disu484 Dineutus subspinosus	10201004	00010	10002	00210	01000	10200	?
Diso605 Dineutus solitarius	10201004	00010	10002	00210	01000	10200	?
Dipx515 Dineutus proximus	10201004	00010	10002	00210	01000	10200	1

Disp576 Dineutus striatus	10201004	00010	10002	00010	01000	10200	?
Dici474 Dineutus ciliatus	10201004	00010	10002	00010	01000	10200	1
Didi473 Dineutus discolor	10201004	00010	10002	00210	01000	10200	1
Disn516 Dineutus sinuosipennis	10201004	00010	10002	00210	01000	10200	1
Disp481 Dineutus aereus	10201004	00010	10002	00210	01000	10200	?
Disu505 Dineutus sublineatus	10201004	00010	10002	00210	01000	10200	1
Diin482 Dineutus indicus	10201004	00010	10002	00210	01000	10200	?
Disp577 Dineutus micans	10201004	00010	10002	00210	01000	10200	?
Ayau483 Macrogyrus oblongus	10001004	01010	10002	00210	10000	10200	1
Aygo501 Macrogyrus gouldi	10001004	01010	10002	00010	10000	10200	?
Ayan502 Macrogyrus australis	10001004	01010	10002	00210	10000	10200	?
Aysp506 Macrogyrus albertisi	10001004	01010	10002	00210	10000	10200	?
Ehas646 Enhydrus atratus	11201004	00010	11002	00210	10000	10200	1
Adsp648 Andogyrus zimmermanni	10201004	01010	10002	00210	10000	10200	?
Prte497 Porrorhynchus marginatus	10201004	00010	10002	00010	01000	10200	1
Ogca666 Orectogyrus camerunensis	20202004	11011	10112	00010	10202	10210	?
Gesp615 Gyretes sericeus	20202004	11011	00112	00200	10110	10200	?
Ogsp566 Orectogyrus noctuabundis	20202004	11011	00112	00010	10101	10210	?
Orvi527 Orectochilus villosus	20202004	10011	00112	00010	10101	10200	1
Berg2 Orectochilus bellieri	20202004	10011	00112	000??	?????	?????	?
Ogcy520 Orectogyrus cyanicollis	20202004	11011	00112	00210	10201	10210	?
Ogha600 Orectogyrus hastatus	20202004	11011	00112	00110	10201	10210	?
Orpr487 Patrus productus	20202004	11011	20112	00200	10010	10200	?
Ordi488 Patrus discifer	20200004	11011	00112	00100	10010	10200	?
Orsp499 Patrus sp	20200004	01011	00112	00200	10210	10200	?
Oran486 Patrus andamanicus	20202004	11011	00112	00200	10210	10200	?
Orvo489 Patrus volubilis	20202004	01011	00112	00200	10210	10200	?
Orsp500 Patrus sp	20202004	11011	00112	00200	10???	10200	?
Orsp677 Patrus sp	20202004	01011	00112	00200	10???	10200	?
Ogar669 Orectogyrus argenteovittatus	20202004	11011	00112	00010	10201	10210	?

Ogsj671 Orectogyrus sjostedti	20202004	11011	10112	00110	10202	10210	?
Ogpi665 Orectogyrus pictimanus	20202004	11011	00112	00010	10201	10210	?
Ogde524 Orectogyrus dedalus	20202004	11011	00112	00010	10101	10210	?
Ogdi491 Orectogyrus discors	20202004	11011	20112	00010	10101	10210	?
Ogos667 Orectogyrus oscari	20202004	11011	20112	00010	10202	10210	?
Ogpl662 Orectogyrus prolongatus	20202004	11011	20112	00010	10???	10210	?
Ogdy664 Orectogyrus demeryi	20202004	11011	20112	00010	10202	10210	?
Ogsp565 Orectogyrus specularis	20202004	11011	20112	00110	10202	10210	?
Ogms670 Orectogyrus masculinus	20202004	11011	20112	00010	10202	10210	?
Ogsp663 Orectogyrus sp	20202004	11011	20112	00010	10???	10210	?
Ogmd601 Orectogyrus madagascariensis	20202004	11011	20112	00110	10202	10210	1
Ogdo517 Orectogyrus dorsiger	20202004	11011	20112	00210	10202	10210	?
Ogsp490 Orectogyrus posticalis	????????	?????	?????	?????	?????	?????	?
Ogsp564 Orectogyrus wittei	20202004	11011	20112	00010	10202	10210	?
Ogbd661 Orectogyrus bedeli	20202004	11011	20112	00110	10202	10210	?
Ogsp567 Orectogyrus specularis	20202004	11011	20112	00110	10202	10210	?
Ogsp668 Orectogyrus sp	????????	?????	?????	?????	?????	?????	?
Ogob595 Orectogyrus oberthuri	20202004	11011	20112	00010	10202	10210	?
Ogse521 Orectogyrus sedilloti	20202004	11011	20112	00110	10202	10210	1
Ogve522 Orectogyrus vestitus	20202004	11011	10112	00110	10202	10210	?
Gesp626 Gyretes sp	20202004	11011	00112	00200	10???	10200	?
Gesp619 Gyretes sp	20202004	11011	00112	00200	10???	10200	?
Gesp616 Gyretes quadrispinosus	20202004	11011	20112	00200	10110	10200	?
Gesp624 Gyretes sp	20202004	11011	00112	00200	10???	10200	?
Geir470 Gyretes iricolor	20202004	11011	00112	00200	10220	10200	0
Gysp686 Gyretes boucardi	20202004	11011	00112	00200	10220	10200	?
Gysp685 Gyretes acutangulus	20202004	11011	00112	00200	10220	10200	?
Gesp617 Gyretes sp	20202004	11011	00112	00200	10???	10200	?
Gesp614 Gyretes sp	20202004	11011	00112	00200	10???	10200	?
Ayhw887 Macrogyrus howittii	10001004	01010	10002	00210	10000	10200	?

Ayre912 Macrogyrus reichei	10001004	01010	10002	00210	10000	10200	1
Ayst882 Macrogyrus striolatus	10001004	01010	10002	00210	10000	10200	1
Adsr886 Andogyrus seriatopunctatus	10001004	01010	10002	00210	10000	10200	?
Adco828 Andogyrus colombicus	10201004	01010	10002	00210	10000	10200	?
AyCs829 Macrogyrus toxopeusi	10201004	01010	10002	00210	10000	10200	?
AyCs841 Macrogyrus purpurascens	10201004	01010	10002	00010	10000	10200	?
Aysp863 Macrogyrus sumbawae	10001004	01010	10002	00210	10000	10200	?
AyTs834 Macrogyrus sp	10001004	01010	10002	00210	10000	10200	?
AyTs833 Macrogyrus sp	10001004	01010	10002	00210	10000	10200	?
AyCs831 Macrogyrus sp	10201004	01010	10002	00010	10000	10200	?
DiDf915 Dineutus fulgidus	10201004	00010	10002	00210	01000	10200	?
DiDn865 Dineutus n sp	10201004	00010	10002	00210	01000	10200	?
DiRt908 Dineutus tetracanthus	10201004	00010	10002	00210	01000	10200	?
Dilo818 Dineutus longimanus	10201004	00010	10002	00010	01000	10200	?
DiCp918 Dineutus pagdeni	10201004	00010	10002	00010	01000	10200	?
Dica821 Dineutus carolinus	10201004	00010	10002	00210	01000	10200	1
Dias819 Dineutus assimilis	10201004	00010	10002	00210	01000	10200	1
Diro913 Dineutus robertsi	10201004	00010	10002	00010	01000	10200	?
Prla852 Porrorhynchus landaisi	10201004	00010	10002	00010	01000	10200	?
Ehsu856 Enhydrus sulcatus	11201004	00010	11002	00210	10000	10200	?
DiCf916 Dineutus fairmairei	10201004	00010	10002	00010	01000	10200	?
Diau911 Dineutus australis	10201004	00010	10002	00210	01000	10200	?
DiMp917 Dineutus priscus	10201004	00010	10002	00210	01000	10200	?
DiMm919 Dineutus macrochirus	10201004	00010	10002	00210	01000	10200	?
Ogor901 Orectogyrs ornaticollis	20202004	11011	20112	00110	10202	10210	1
Oghe900 Orectogyrus heros	20202004	11011	20112	00110	10202	10210	1
Pasp897 Patrus sp	20202004	11011	20112	0020?	?????	?????	0
Pasp896 Patrus sp	20202004	10011	20112	0020?	?????	?????	0
Pasp898 Patrus sp	20202004	11011	20112	0010?	?????	?????	0

Gysp840 Gyrinus sericeolimbatus	202?0004	11011	10002	00201	00000	00201	?
Gysp839 Gyrinus dimorphus	20210004	11011	10002	00201	00000	00201	?
Gysp837 Gyrinus maculiventris	20210004	11011	10002	00101	00000	00201	?
Agmo Angarogyrus mongolicus	???????1	?????	??0??	?0???	?????	?????	?
Agmi Angarogyrus minimus	????????	?????	??0??	?????	?????	?????	?
Basa Baissogyrus savilovi	101?100?	00011	1000?	??0??	?????	?????	?
Mgan Mesogyrus antiquus	1010100?	00011	1000?	??0??	?????	?????	?
Mgst Mesogyrus striatus	10??100?	0?011	1?0??	?????	?????	?????	?
Crzh Cretotortor zherichini	????????	?????	?????	?????	?????	?????	?
Meam Mesodineutes amurensis	1000100?	00010	10002	00???	?????	?????	?
Gegi Gyretes giganteus	????????	?????	??1??	00???	?????	?????	?
Miin Miodineutes insignis	????????	?????	??1??	00???	?????	?????	?
Cresp Cretotortor sp	????????	?????	??0??	?0???	?????	?????	?

Table S2: Dated Endemic Malagasy lineages assembled from the literature. The endemic Madagascar clades sometimes contains species inferred as secondary dispersals out of Madagascar, e.g. to neighbouring islands in the Comoros or Mascarene archipalegos. Ages, in million years, represent stem ages except when a "*" indicates that the age refer to crown age. Lower and upper age estimates are as given from the study, most often they represent the 95% HPD interval, but in a few case the standard deviation. Gene source indicates nc = nuclear, mt = mitochondrial or cp = chloroplast genes. The same endemic taxon from the same study may occur multiple times due to alternative analytical settings.

Organism	Higher cl./vern. name	Endemic taxon	Age	Lower	Upper	Gene src.	Reference
Birds	Elephant birds	Aepyornithidae	**50**	**40.1**	**61.5**	mt	Mitchell et al. 2016
	Vangas	Vangidae	**18.5**	**16.6**	**20.0**	mt	Reddy et al. 2012
	Vangas	Vangidae	**25***			mt&nc	Jönsson et al. 2012
	Vangas	Vangidae	**28.9**	**24.9**	**32.9**	mt&nc	Fuchs et al 2006
	Vangas	Vangidae	**28.3**	**23.4**	**33.2**	nc	Fuchs et al 2006
	Vangas	Vangidae	**19.7**	**16.8**	**27.0**	nc	Beresford et al. 2005
	Mesites	Mesitornithidae	**54**	**30**	**64**	nc	Prum et al. 2015
	Asitites	Philepittidae	**19**	**7**	**32**	nc	Prum et al. 2015
	Ground rollers	Brachypteraciidae	**34**	**14**	**49**	nc	Prum et al. 2015
	Cuckoo rollers	Leptosomidae	**59**	**55**	**63**	nc	Prum et al. 2015
	Sunbirds	Nectariniidae (*Nectarinia s. souimanga*)	**0.48**			mt	Warren et al. 2003
	Sunbirds	Nectariniidae (*Nectarinia s. souimanga*)	**0.66**			mt	Warren et al. 2003
	Sunbirds	Nectariniidae (*Nectarinia s. souimanga*)	**0.78**			mt	Warren et al. 2003
	Sunbirds	Nectariniidae (*Nectarinia s. souimanga*)	**0.51**			mt	Warren et al. 2003
	Sunbirds	Nectariniidae (*Nectarinia s. souimanga*)	**0.94**			mt	Warren et al. 2003
	Sunbirds	Nectariniidae (*Nectarinia s. souimanga*)	**0.69**			mt	Warren et al. 2003
	Parrots	Psittaculidae (*Caracopsis*)	**67**	**58**	**76**	mt&nc	Wright et al. 2008
	Parrots	Psittaculidae (*Caracopsis*)	**42**	**35.5**	**47.5**	mt&nc	Wright et al. 2008
	Parrots	Psittaculidae (*Caracopsis*)	**62.5**	**62**	**63**	mt&nc	Wright et al. 2008
	Parrots	Psittaculidae (*Caracopsis*)	**39**	**39**	**40**	mt&nc	Wright et al. 2008
	Malagasy songbirds	Bernieridae	**25.2**	**21.4**	**31.7**	mt&nc	Beresford et al. 2005
	Wagtails	Motacillidae (*Motacilla flaviventris*)	**4.5**			mt	Voelker 2002
	Bulbuls	Pycnonotidae (*Hypsipetes madagascariensis*)		**0.4**	**1.8**	mt	Warren et al. 2005
	White-eye songbirds	Zosteropidae (*Zosterops maderaspatanus*)	**0.44**			mt	Warren et al. 2005
Centipedes	Scutigeromorph centipedes	Scutigerinidae (Madagascar *Scutigerina*)	**158**	**120**	**199**	mt&nc	Giribet & Edgecombe 2013
	Scutigeromorph centipedes	Scutigeridae (*Lassophora nossibei*)	**133**	**95**	**165**	mt&nc	Giribet & Edgecombe 2013
Decapods	Freshwater crayfish	Parastacidae (*Astacoides*)	**147**	**122**	**169**	mt&nc	Toon et al. 2010
	Freshwater crabs	Madagascan Potamonautidae		**73.1**	**76.2**	mt	Daniels et al. 2006
Fish	Cichlid fish	Cichlidae (Ptychochrominae)	**96**	**78**	**115**	mt	Azuma et al 2008
	Cichlid fish	Cichlidae (*Paretroplus*)	**87**	**69**	**106**	mt	Azuma et al 2008
	Cichlid fish	Cichlidae (*Paretroplus*)	**27**	**5**	**43**	mt&nc	Vences et al. 2001
	Cichlid fish	Cichlidae (*Paretroplus*)	**69.5**	**53.1**	**85.9**	mt&nc	Matschiner et al. 2016
	Cichlid fish	Cichlidae (Ptychochrominae)	**85.7**	**77.8**	**93.8**	mt&nc	Matschiner et al. 2016
	Cichlid fish	Madagascar Cichlidae	**58**	**38**	**86**	nc	Crottini et al. 2012
	Cichlid fish	Madagascar Cichlidae	**76**	**46**	**103**	nc	Crottini et al. 2012
	Madagascar rainbowfish	Bedotiidae	**42**	**24**	**68**	nc	Crottini et al. 2012
	Madagascar rainbowfish	Bedotiidae	**51**	**26**	**82**	nc	Crottini et al. 2012
	Cyprinodontiform fish	Madagascar Aplocheilidae	**41**	**24**	**65**	nc	Crottini et al. 2012

	Cyprinodontiform fish	Madagascar Aplocheilidae	**53**	**28**	**82**	nc	Crottini et al. 2012
Amphibians	Mantellid frogs	Mantellidae		**56.0**	**86.2**	nc	Van Bocxlaer et al. 2006
	Mantellid frogs	Mantellidae		**50.5**	**62.8**	nc	Van Bocxlaer et al. 2006
	Mantellid frogs	Mantellidae	**73.1**	**51.6**	**100.1**	nc	van der Meijden et al. 2005
	Mantellid frogs	Mantellidae	**64**			nc	Vences et al. 2003
	Mantellid frogs	Mantellidae	**82.7**	**58.5**	**111.8**	mt&nc	Bossuyt et al. 2006
	Mantellid frogs	Mantellidae	**76**	**50**	**108**	nc	Crottini et al. 2012
	Mantellid frogs	Mantellidae	**87**	**55**	**122**	nc	Crottini et al. 2012
	Microhylid frogs	Microhylidae (Dyscophinae)		**65.1**	**77.6**	nc	Van Bocxlaer et al. 2006
	Microhylid frogs	Microhylidae (Dyscophinae)		**65.0**	**65.0**	nc	Van Bocxlaer et al. 2006
	Microhylid frogs	Microhylidae (Dyscophinae)	**55**	**39**	**76**	nc	van der Meijden et al. 2007
	Microhylid frogs	Microhylidae (Dyscophinae)	**62**	**37**	**94**	nc	Crottini et al. 2012
	Microhylid frogs	Microhylidae (Dyscophinae)	**65**	**38**	**95**	nc	Crottini et al. 2012
	Microhylid frogs	Microhylidae (Cophylinae)		**48.7**	**75.8**	nc	Van Bocxlaer et al. 2006
	Microhylid frogs	Microhylidae (Cophylinae)		**49.6**	**66.1**	nc	Van Bocxlaer et al. 2006
	Microhylid frogs	Microhylidae (Scaphiophryninae)		**65.4**	**88.1**	nc	Van Bocxlaer et al. 2006
	Microhylid frogs	Microhylidae (Scaphiophryninae)		**51.7**	**69.1**	nc	Van Bocxlaer et al. 2006
	Microhylid frogs	Microhylidae (Cophylinae/ Scaphiophryninae)	**77**	**49**	**114**	nc	Crottini et al. 2012
	Microhylid frogs	Microhylidae (Cophylinae/ Scaphiophryninae)	**92**	**63**	**123**	nc	Crottini et al. 2012
	Microhylid frogs	Microhylidae (Cophylinae/ Scaphiophryninae)	**55**	**40**	**76**	nc	van der Meijden et al. 2007
	Hyperoliid frogs	Hyperoliidae (*Heterixalus*)		**19**	**30**	nc	Vences et al. 2003
	Hyperoliid frogs	Hyperoliidae (*Heterixalus*)	**57**	**30**	**94**	nc	Crottini et al. 2012
	Hyperoliid frogs	Hyperoliidae (*Heterixalus*)	**53**	**25**	**85**	nc	Crottini et al. 2012
	Grassland frogs	Ptychadenidae	**8**	**2**	**20**	nc	Crottini et al. 2012
	Grassland frogs	Ptychadenidae	**13**	**4**	**24**	nc	Crottini et al. 2012
Insects	Ants	Formicidae (*Adetomyrna*)	**42**	**37.1**	**46.9**	nc	Brady et al. 2006
	Ants	Formicidae (*Adetomyrna*)	**46**	**40.1**	**51.9**	nc	Brady et al. 2006
	Ants	Formicidae (*Adetomyrna*)	**43**	**38.6**	**48.4**	nc	Brady et al. 2006
	Ants	Formicidae (*Adetomyrna*)	**49**	**43.2**	**54.8**	nc	Brady et al. 2006
	Ants	Formicidae (*Adetomyrna*)	**53**	**45.9**	**60.1**	nc	Brady et al. 2006
	Ants	Formicidae (*Adetomyrna*)	**49**	**42.6**	**55.4**	nc	Brady et al. 2006
	Allodapine bees	Apidae (*Hasinamelisa*)	**38**			mt&nc	Chenoweth & Schwarz 2011
	Allodapine bees	Apidae (Madagascan *Macrogalea*)	**9**			mt&nc	Chenoweth & Schwarz 2011
	Carpenter bees	Apidae (*Hirashima sp.*)	**23***	**14***	**32***	mt&nc	Rehan et al. 2010
	Carpenter bees	Apidae (*Hirashima sp.*)	**15***	**6***	**24***	mt&nc	Rehan et al. 2010
	Carpenter bees	Apidae (*Malgatina*)	**25**	**16.6**	**33.4**	mt&nc	Rehan et al. 2010
	Carpenter bees	Apidae (*Malgatina*)	**19**	**4**	**36**	mt&nc	Rehan et al. 2010
	Allodapine bees	Apidae (*Halterapis*)	**43**	**27**	**60**	mt&nc	Schwarz et al. 2006
	Allodapine bees	Apidae (*Halterapis*)	**31**	**28**	**44**	mt&nc	Schwarz et al. 2006
	Termites	Termitidae (Madagascan *Microtermes)*	**13.2**	**3.3**	**18.8**	mt&nc	Nobre et al. 2010
	Butterflies	Nymphalidae (Madagascan *Heteropsis* clade1)	**23**	**18.7**	**32.4**	mt&nc	Kodandaramaiah et al. 2010
	Butterflies	Nymphalidae (Madagascan *Heteropsis* clade2)	**14.1**	**11.2**	**23**	mt&nc	Kodandaramaiah et al. 2010

Butterflies	Nymphalidae (*Euxanthe madagascariensis*)	**3.2**	**2.1**	**4.3**	mt&nc	Aduse-Poku et al. 2009
Butterflies	Nymphalidae (*Charaxes antamboulous*)	**3.7**	**2.1**	**5.9**	mt&nc	Aduse-Poku et al. 2009
Swallowtail butterflies	Papilionidae (*Papilio dardanus meriones*)	**0.5**			mt&nc	Clark & Vogler 2009
Millipede assassin bugs	Reduviidae (*Gibbosella quadocris*	**30**	**13**	**46**	mt	Forthman & Weirauch 2016
Millipede assassin bugs	Reduviidae (*Toxopus+Tanindrazanus+ Marojejycoris*)	**30**	**19**	**43**	mt	Forthman & Weirauch 2016
Millipede assassin bugs	Reduviidae (*Distirogaster*)	**18**	**9**	**28**	mt	Forthman & Weirauch 2016
Millipede assassin bugs	Reduviidae (*Glymmatophora crassipes*)	**21**	**13**	**30**	mt	Forthman & Weirauch 2016
Dung beetles	Scarabaeidae (Helictopleurini)	**44**	**29**	**64**	mt	Wirta et al. 2008
Dung beetles	Scarabaeidae (Helictopleurini)	**28**	**18**	**39**	mt	Wirta et al. 2008
Dung beetles	Scarabaeidae (Helictopleurini)	**61.1-66.1**			mt&nc	Gunter et al. 2016
Dung beetles	Scarabaeidae (*Helictopleurus*)		**56.1-60.9**		mt&nc	Gunter et al. 2016
Dung beetles	Scarabaeidae (*Epactoides*)	**38.0**	**20.2**	**62.8**	mt	Wirta et al. 2010
Dung beetles	Scarabaeidae (*Epactoides*)	**23.8**	**12.6**	**39.2**	mt	Wirta et al. 2010
Dung beetles	Scarabaeidae (*Arachnodes* and *Epilissus*)	**40.2***	**25.7***	**55.1***	mt	Wirta et al. 2010
Dung beetles	Scarabaeidae (*Arachnodes* and *Epilissus*)	**64.0***	**43.6***	**86.0***	mt	Wirta et al. 2010
Dung beetles	Scarabaeidae (*Apotolamprus* and *Nanos*)	**23.5***	**14.5***	**35.8***	mt	Wirta et al. 2010
Dung beetles	Scarabaeidae (*Apotolamprus* and *Nanos*)	**14.7***	**9.0***	**22.3***	mt	Wirta et al. 2010
Dung beetles	Scarabaeidae (Madagascar Scarabaeini)	**15.2**	**11.9**	**18.8**	mt	Sole et al. 2011
Dung beetles	Scarabaeidae (Madagascar Scarabaeini)	**24.2**	**18.9**	**29.8**	mt	Sole et al. 2011
Water scavenger beetles	Malagasy cascade beetles (*Tritonus*)	**91.6**	**63.7**	**126.8**	mt&nc	Toussaint et al. 2016
Diving beetles	Dytiscidae (*Hydaticus ornatus*)	**10**	**5**	**16**	mt&nc	Bukontaite et al. 2015
Diving beetles	Dytiscidae (*Hydaticus lineatus*)	**29**	**19**	**39**	mt&nc	Bukontaite et al. 2015
Diving beetles	Dytiscidae (*Hydaticus kolbei*)	**8**	**6**	**11**	mt&nc	Bukontaite et al. 2015
Diving beetles	Dytiscidae (*Hydaticus sobrinus*)	**23**	**17**	**29**	mt&nc	Bukontaite et al. 2015
Diving beetles	Dytiscidae (*Hydaticus nigrotaeniatus*)	**31**	**23**	**42**	mt&nc	Bukontaite et al. 2015
Diving beetles	Dytiscidae (*Hydaticus petiti*)	**23**	**17**	**30**	mt&nc	Bukontaite et al. 2015
Diving beetles	Dytiscidae (*Hydaticus madagascariensis*)	**6**	**3**	**9**	mt&nc	Bukontaite et al. 2015
Diving beetles	Dytiscidae (*Cybister guignoti*)	**10**	**5**	**16**	mt&nc	Bukontaite et al. 2015
Diving beetles	Dytiscidae (*Cybister tibialis*)	**16**	**10**	**23**	mt&nc	Bukontaite et al. 2015
Diving beetles	Dytiscidae (*Cybister operosus*)	**29**	**20**	**39**	mt&nc	Bukontaite et al. 2015
Whirligig beetles	Gyrinidae (Heterogyrinae)	**206**	**186.8**	**225.7**	mt&nc	this study
Whirligig beetles	Gyrinidae (Malagasy *Aulonogyrus*)	**46.6**	**35.2**	**56.0**	mt&nc	this study

	Whirligig beetles	Gyrinidae (*Gyrinus ignitus*)	**32.9**	**18.6**	**49.7**	mt&nc	this study
	Whirligig beetles	Gyrinidae (*Gyrinus madagascariensis*)	**28.5**	**16.5**	**41.6**	mt&nc	this study
	Whirligig beetles	Gyrinidae (*Dineutus proximus*)	**21.9**	**12.5**	**33.3**	mt&nc	this study
	Whirligig beetles	Gyrinidae (*Dineutus sinuosipennis*)	**42.8**	**31.9**	**54.3**	mt&nc	this study
	Whirligig beetles	Gyrinidae (*Orectogyrus heros* + *O. madagascariensis*)	**20.7**	**15.2**	**27.2**	mt&nc	this study
	Whirligig beetles	Gyrinidae (*Orectogyrus vestitus* + *O. oberthuri*)	**37.5**	**25.3**	**49.9**	mt&nc	this study
	Whirligig beetles	Gyrinidae (*Orectogyrus sedilloti* + *O. ornaticollis*)	**44.5**	**32.3**	**57.1**	mt&nc	this study
	Whirligig beetles	Gyrinidae (*Orectogyrus hastatus* + *O. cyanicollus*)	**87.6**	**68.8**	**108.0**	mt&nc	this study
Mammals	Lemurs	Lemuriformes	**72.9**	**64.0**	**82.0**	mt	Yoder & Yang 2004
	Lemurs	Lemuriformes	**68.5**	**61.3**	**75.4**	mt&nc	Yoder & Yang 2004
	Lemurs	Lemuriformes	**70.1**	**56.8**	**83.8**	nc	Yoder & Yang 2004
	Lemurs	Lemuriformes	**74.9**	**66.1**	**83.0**	nc	Yoder & Yang 2004
	Lemurs	Lemuriformes	**66**	**55**	**75**	mt&nc	Yoder et al. 2003
	Lemurs	Lemuriformes	**62**	**47**	**75**	mt&nc	Yoder et al. 2003
	Lemurs	Lemuriformes	**64**	**50**	**78**	mt&nc	Yoder et al. 2003
	Lemurs	Lemuriformes	**60.4**	**51.6**	**69.6**	nc	Poux et al. 2005
	Lemurs	Lemuriformes	**71**	**51**	**94**	nc	Crottini et al. 2012
	Lemurs	Lemuriformes	**73**	**46**	**103**	nc	Crottini et al. 2012
	Lemurs	Lemuriformes	**75**	**66.9**	**84.4**	mt&nc	Horvath et al. 2008
	Lemurs	Lemuriformes	**46**	**41**	**51**	mt&nc	Chatterjee et al. 2009
	Lemurs	Lemuriformes	**59**	**39**	**77**	nc	Perelman et al. 2011
	Lemurs	Lemuriformes	**50**	**49**	**51**	mt&nc	Springer et al. 2012
	Lemurs	Lemuriformes	**50**	**42**	**57**	mt	Kistler et al. 2015
	Lemurs	Lemuriformes	**55**	**49**	**61**	mt&nc	Herrera & Dávalos 2016
	Carnivores	Eupleridae	**20**	**15**	**26**	mt&nc	Yoder et al. 2003
	Carnivores	Eupleridae	**23**	**15**	**32**	mt&nc	Yoder et al. 2003
	Carnivores	Eupleridae	**20**	**11**	**31**	mt&nc	Yoder et al. 2003
	Carnivores	Eupleridae	**24**	**16**	**33**	mt&nc	Yoder et al. 2003
	Carnivores	Eupleridae	**18**	**11**	**25**	mt&nc	Yoder et al. 2003
	Carnivores	Eupleridae	**24**	**18**	**31**	mt&nc	Yoder et al. 2003
	Carnivores	Eupleridae	**25.9**	**20.1**	**32.5**	nc	Poux et al. 2005
	Carnivores	Eupleridae	**26**	**16**	**38**	nc	Crottini et al. 2012
	Carnivores	Eupleridae	**26**	**14**	**39**	nc	Crottini et al. 2012
	Tenrecs	Madagascan Tenrecidae	**41.8**	**34.1**	**50.3**	nc	Poux et al. 2005
	Tenrecs	Madagascan Tenrecidae	**43**	**34**	**52**	nc	Doudy et al. 2003
	Tenrecs	Madagascan Tenrecidae	**53**	**51**	**55**	mt&nc	Doudy et al. 2002
	Tenrecs	Madagascan Tenrecidae	**47**	**40**	**55**	nc	Poux et al. 2008
	Tenrecs	Madagascan Tenrecidae	**45**	**37**	**54**	nc	Poux et al. 2008
	Tenrecs	Tenrecidae	**60**	**40**	**84**	nc	Crottini et al. 2012
	Tenrecs	Tenrecidae	**101**	**62**	**142**	nc	Crottini et al. 2012
	Bats	Myzopodidae	**52**	**46**	**57**	nt	Teeling et al. 2005
	Rodents	Muridae (Nesomyinae)	**23.5**	**18.2**	**29.6**	nc	Poux et al. 2005
	Rodents	Muridae (Nesomyinae)	**28**	**17**	**42**	nc	Crottini et al. 2012
	Rodents	Muridae (Nesomyinae)	**47**	**26**	**71**	nc	Crottini et al. 2012
Ferns	Cyatheales	Cyatheaceae (*Gymnosphaera* clade)	**13.4**	**10.9**	**15.8**	cp	Janssen et al. 2008

	Cyatheales	Cyatheaceae (bipinnate clade)	**30.3**	**27.3**	**33.3**	cp	Janssen et al. 2008
	Cyatheales	Cyatheaceae (tripinnate clade)	**19.5**	**16.2**	**22.9**	cp	Janssen et al. 2008
Angiosperms	Asterales	Asteraceae (*Conyza necandolleana*)	**5**			cp&nc	Strijk et al. 2012
	Asterales	Asteraceae (*Psiadia* clade A)	**10**			cp&nc	Strijk et al. 2012
	Malvales	Malvaceae (Megistohibiscus clade)	**16.7**	**8.3**	**26.5**	cp	Koopman & Baum 2008
	Laurales	Monimiaceae (*Tambourissa clade*)	**35.8**			cp	Renner et al. 2010
	Cucurbitales	Cucurbitaceae (*Xerosicyos* clade)	**49**	**40**	**57**	cp	Schaefer et al. 2009
	Cucurbitales	Cucurbitaceae (*Ampelosicyos* clade)	**29**	**19**	**39**	cp	Schaefer et al. 2009
	Cucurbitales	Cucurbitaceae (*Muellerargia jeffreyana*)	**12**	**7**	**18**	cp	Schaefer et al. 2009
	Ericales	Sapotaceae (*Chrysophyllum boivinianum*)	**61.0**	**53.8**	**68.2**	cp	Bartish et al. 2011
	Apiales	Torricelliaceae	**52.4**	**27.0**	**64.3**	cp&nc	Magallón et al. 2015
	Apiales	Torricelliaceae (*Melanophylla*)	**64.0**			cp&nc	Magallón et al. 2015
	Apiales	Torricelliaceae (*Melanophylla*)	**55.8**			cp	Tank et al. 2015
	Apiales	Torricelliaceae (*Melanophylla*)	**60**	**60**	**65**	cp&nc	Wikström et al 2001
	Buxales	Buxaceae (*Didymeles*)	**110.8**			cp&nc	Magallón et al. 2015
	Buxales	Buxaceae (*Didymeles*)	**85.1**	**50.4**	**115.9**	cp&nc	Magallón et al. 2015
	Buxales	Buxaceae (*Didymeles*)	**101.5**			cp	Tank et al. 2015
	Buxales	Buxaceae (*Didymeles*)	**55.3**			cp	Zanne et al. 2014
	Buxales	Buxaceae (*Didymeles*)	**113**	**113**	**124**	cp&nc	Wikström et al 2001
	Sapindales	Burseraceae (Madagascar *Canarium* clade)	**10.9**	**7.1**	**16.1**	cp&nc	Federman et al. 2015
	Canellales	Canellaceae (Cinnamosma)	**25**	**15**	**35**	cp&nc	Müller et al. 2015
	Canellales	Winteraceae (*Takhtajania*)	**68.9**			cp&nc	Magallón et al. 2015
	Canellales	Winteraceae (*Takhtajania*)	**29.7**	**11.5**	**69.3**	cp&nc	Magallón et al. 2015
	Canellales	Winteraceae (*Takhtajania*)	**55.4**			cp	Tank et al. 2015
	Canellales	Winteraceae (*Takhtajania*)	**55.4**			cp	Zanne et al. 2014
	Canellales	Winteraceae (*Takhtajania*)	**49**	**49**	**52**	cp&nc	Wikström et al 2001
	Canellales	Winteraceae (*Takhtajania*)	**62**	**35**	**91**	cp&nc	Müller et al. 2015
	Caryophyllales	Didieraceae (Didieroideae)	**36.8**	**22.8**	**58.2**	cp&nc	Magallón et al. 2015
	Caryophyllales	Didieraceae (Didieroideae)	**58.1**			cp&nc	Magallón et al. 2015
	Caryophyllales	Didieraceae (Didieroideae)	**26.0**			cp	Tank et al. 2015
	Caryophyllales	Didieraceae (Didieroideae)	**26.0**			cp	Zanne et al. 2014
	Caryphyllales	Asteropeiaceae	**58**	**52**	**61**	cp&nc	Wikström et al 2001
	Caryphyllales	Asteropeiaceae + Physenaceae	**95.6**			cp&nc	Magallón et al. 2015
	Caryphyllales	Asteropeiaceae + Physenaceae	**96.2**	**91.2**	**101.6**	cp&nc	Magallón et al. 2015
	Caryphyllales	Asteropeiaceae + Physenaceae	**77.5**			cp	Tank et al. 2015

	Caryphyllales	Asteropeiaceae + Physenaceae	**77.5**			cp	Zanne et al. 2014
	Caryphyllales	Barbeuiaceae	**76.7**	**74.9**	**78.6**	cp&nc	Magallón et al. 2015
	Caryphyllales	Barbeuiaceae	**77.8**	**74.5**	**81.5**	cp&nc	Magallón et al. 2015
	Caryphyllales	Barbeuiaceae	47.93	43.83	54.35	cp	Tank et al. 2015
	Caryphyllales	Barbeuiaceae	**33.8**			cp	Zanne et al. 2014
	Malvales	Bixaceae (*Diegodendron*)	**42.4**			cp	Zanne et al. 2014
	Malvales	Sarcolaenaceae	**42.0**			cp	Zanne et al. 2014
	Malvales	Sarcolaenaceae	**28**	**14**	**28**	cp&nc	Wikström et al 2001
	Malvales	Sphaerosepalaceae (*Dialyceras* + *Rhopalocarpus*)	**67.2**			cp	Zanne et al. 2014
	Proteales	Proteaceae (*Dilobeia*)	**14.3**			cp	Zanne et al. 2014
	Proteales	Proteaceae (*Malagasia*)	**9.0**			cp	Zanne et al. 2014
	Solanales	Concolvulaceae (*Humbertia*)	**54.1**			cp	Zanne et al. 2014
	Solanales	Montiniaceae (*Kaliphora*)	**27.9**			cp	Zanne et al. 2014
Squamata	Blindsnakes	Xenotyphlopidae	**61**	**42**	**82**	nc	Crottini et al. 2012
	Blindsnakes	Xenotyphlopidae	**66**	**43**	**90**	nc	Crottini et al. 2012
	Blindsnakes	Xenotyphlopidae	**97**	**81**	**112**	nc	Vidal et al. 2010
	Blindsnakes	Typhlopidae (Madagascan *Typholps*)	**54**	**36**	**74**	nc	Crottini et al. 2012
	Blindsnakes	Typhlopidae (Madagascan *Typholps*)	**39**	**21**	**59**	nc	Crottini et al. 2012
	Blindsnakes	Typhlopidae (Madagascan *Typholps*)	**61.5**	**48**	**76.5**	nc	Vidal et al. 2010
	Boas	Boidae (*Acrantophis+Sanzinia*)	**61**	**48**	**75**	nc	Crottini et al. 2012
	Boas	Boidae (*Acrantophis+Sanzinia*)	**47**	**22**	**74**	nc	Crottini et al. 2012
	Boas	Boidae (*Acrantophis+Sanzinia*)	**77**	**68**	**89**	mt&nc	Noonan & Chippindale 2006
	Lamprophiidae snakes	Pseudoxyrhophiinae	**24**	**14**	**37**	nc	Crottini et al. 2012
	Lamprophiidae snakes	Pseudoxyrhophiinae	**28**	**15**	**41**	nc	Crottini et al. 2012
	Lamprophiidae snakes	Pseudoxyrhophiinae	**30.8**	**21.5**	**75.9**	mt&nc	Nagy et al. 2003
	Lamprophiidae snakes	Psammophiinae (*Mimophis*)	**19**	**10**	**31**	nc	Crottini et al. 2012
	Lamprophiidae snakes	Psammophiinae (*Mimophis*)	**22**	**10**	**37**	nc	Crottini et al. 2012
	Lamprophiidae snakes	Psammophiinae (*Mimophis*)	**12.9**	**8.2**	**29.5**	mt&nc	Nagy et al. 2003
	Madagascar iguanas	Opluridae	**90**	**62**	**120**	nc	Crottini et al. 2012
	Madagascar iguanas	Opluridae	**72**	**32**	**117**	nc	Crottini et al. 2012
	Madagascar iguanas	Opluridae	**162**	**147**	**178**	mt	Okajima & Kumazawa 2009
	Madagascar iguanas	Opluridae	**90**	**67**	**118**	nc	Noonan & Chippindale 2006
	Madagascar iguanas	Opluridae	**53**	**44**	**62**	nc	Townsend etal. 2011
	Chameleons	Chamaeleonidae	**54**	**34**	**77**	nc	Crottini et al. 2012
	Chameleons	Chamaeleonidae	**54**	**34**	**75**	nc	Crottini et al. 2012
	Chameleons	Chamaeleonidae (*Brookesia*)	**65**	**73**	**57**	mt&nc	Tolley et al. 2013

	Chameleons	Chamaeleonidae (*Calumma+Furcifer*)	**47**	**40**	**54**	mt&nc	Tolley et al. 2013
	Plated lizards	Gerrhosauridae (Zonosaurinae)	**61**	**32**	**96**	nc	Crottini et al. 2012
	Plated lizards	Gerrhosauridae (Zonosaurinae)	**37**	**17**	**63**	nc	Crottini et al. 2012
	Plated lizards	Gerrhosauridae (Zonosaurinae)	**66**	**53**	**85**	mt&nc	Raselimanana et al. 2009
	Plated lizards	Gerrhosauridae (Zonosaurinae)	**29**	**17**	**45**	mt&nc	Blair et al. 2015
	Plated lizards	Gerrhosauridae (Zonosaurinae)	**36**	**19**	**54**	mt&nc	Blair et al. 2015
	Plated lizards	Gerrhosauridae (Zonosaurinae)	**19**	**15**	**25**	mt&nc	Blair et al. 2015
	Skinks	Scincidae (Madagascan *Trachylepis*)	**19**	**9**	**37**	nc	Crottini et al. 2012
	Skinks	Scincidae (Madagascan *Trachylepis*)	**24**	**10**	**39**	nc	Crottini et al. 2012
	Skinks	Scincidae (Madagascan Scincinae)	**65**	**39**	**96**	nc	Crottini et al. 2012
	Skinks	Scincidae (Madagascan Scincinae)	**47**	**23**	**70**	nc	Crottini et al. 2012
	Geckos	Gekkonidae (*Phelsuma*)	**62**	**39**	**91**	nc	Crottini et al. 2012
	Geckos	Gekkonidae (*Phelsuma*)	**49**	**34**	**65**	nc	Crottini et al. 2012
	Geckos	Gekkonidae (*Lygodactylus*)	**62**	**39**	**91**	nc	Crottini et al. 2012
	Geckos	Gekkonidae (*Lygodactylus*)	**49**	**34**	**65**	nc	Crottini et al. 2012
	Geckos	Gekkonidae (*Blaesodactylus*)	**42**	**23**	**68**	nc	Crottini et al. 2012
	Geckos	Gekkonidae (*Blaesodactylus*)	**27**	**10**	**45**	nc	Crottini et al. 2012
	Geckos	Gekkonidae (*Paroedura*)	**57**	**34**	**86**	nc	Crottini et al. 2012
	Geckos	Gekkonidae (*Paroedura*)	**43**	**27**	**60**	nc	Crottini et al. 2012
	Geckos	Gekkonidae (Madagascan *Hemidactylus mercatorius*)	**4**	**1**	**10**	nc	Crottini et al. 2012
	Geckos	Gekkonidae (Madagascan *Hemidactylus mercatorius*)	**6**	**1**	**11**	nc	Crottini et al. 2012
	Leaf-tailed geckos	Gekkonidae (*Uroplatus*)	**51**	**29**	**78**	nc	Crottini et al. 2012
	Leaf-tailed geckos	Gekkonidae (*Uroplatus*)	**38**	**20**	**58**	nc	Crottini et al. 2012
	Leaf-tailed geckos	Gekkonidae (*Uroplatus*)	**40.5**	**29.8**	**52.5**	mt&nc	Raxworthy et al. 2008
	Leaf-tailed geckos	Gekkonidae (*Uroplatus*)	**46.4**	**32.9**	**61.1**	mt&nc	Raxworthy et al. 2008
	Leaf-tailed geckos	Gekkonidae (*Uroplatus*)	**38.9**	**27.3**	**51.1**	mt&nc	Raxworthy et al. 2008
Testudines	Turtles	Podocnemididae (*Erymnochelys*)	**112**	**73**	**159**	nc	Crottini et al. 2012
	Turtles	Podocnemididae (*Erymnochelys*)	**87**	**65**	**111**	nc	Crottini et al. 2012
	Turtles	Podocnemididae (*Erymnochelys*)	**78.5**			mt&nc	Vargas-Ramirez et al. 2008
	Turtles	Podocnemididae (*Erymnochelys*)	**76**	**66**	**96**	mt&nc	Noonan & Chippindale 2006
	Tortoises	Testudinidae (*Pyxis+Astrochelys*)	**79**	**33**	**134**	nc	Crottini et al. 2012
	Tortoises	Testudinidae (*Pyxis+Astrochelys*)	**16**	**6**	**30**	nc	Crottini et al. 2012
	Tortoises	Testudinidae (*Pyxis+Astrochelys+Aldabrachelys*)		**11.5**	**17.5**	mt	Palkovacs et al. 2002

	Tortoises	Testudinidae (*Pyxis+Astrochelys*)		**14**	**22**	mt	Caccone et al. 1998
Crocodilia	Crocodiles	Crocodylidae (Madagascan *Crocodylus niloticus*)	**5**	**0**	**20**	nc	Crottini et al. 2012
	Crocodiles	Crocodylidae (Madagascan *Crocodylus niloticus*)	**1**	**0**	**2**	nc	Crottini et al. 2012
Spiders	Golden orb weaver spiders	Nephilidae (Madagascan *Nephila*)	**2.46**	**0.6**	**5.3**	mt&nc	Kuntner & Agnarsson 2011a
	Hermit spiders	Nephilidae (Madagascan *Nephilengys*)	**1.9**		**7.4**	mt&nc	Kuntner & Agnarsson 2011b
	Pelican spiders	Archaeidae (*Eriauchenius*)	**136**	**100**	**173**	mt&nc	Wood et al. 2015
	Pelican spiders	Archaeidae (*Gracilicollis* group)	**154**	**115**	**191**	mt&nc	Wood et al. 2015
Snails	Caenogastropoda	Pachychilidae (*Madagasikara*)	**25.5**	**20.3**	**31.5**	mt	Köhler & Glaubrecht 2010
Flatworms	Monogenean flatworms	Polystomatidae (Madagascan *Metapolystoma*)	**8.6**	**4.3**	**14.2**	mt&nc	Verneau et al. 2009
	Monogenean flatworms	Polystomatidae (*Madapolystoma*)	**116.2**	**95.6**	**134.6**	mt&nc	Verneau et al. 2009

Table S2 – References

Aduse-Poku, K., Vingerhoedt, E. & Wahlberg, N. Out-of-Africa again: a phylogenetic hypothesis of the genus Charaxes (Lepidoptera: Nymphalidae) based on five gene regions. Molecular Phylogenetics and Evolution **53**, 463–478 (2009).

Azuma, Y., Kumazawa, Y., Miya, M., Mabuchi, K. & Nishida, M. Mitogenomic evaluation of the historical biogeography of cichlids toward reliable dating of teleostean divergences. BMC Evolutionary Biology **8**, 215 (2008).

Bartish, I. V., Antonelli, A., Richardson, J. E. & Swenson, U. Vicariance or long-distance dispersal: historical biogeography of the pantropical subfamily Chrysophylloideae (Sapotaceae). Journal of Biogeography **38**, 177–190 (2011).

Beresford, P., Barker, F. K., Ryan, P. G. & Crowe, T. M. African endemics span the tree of songbirds (Passeri): molecular systematics of several evolutionary 'enigmas'. Proceedings of the Royal Society B **272**, 849–858 (2005).

Blair, C. et al. Multilocus phylogenetic and geospatial analyses illuminate diversification patterns and the biogeographic history of Malagasy endemic plated lizards (Gerrhosauridae: Zonosaurinae). Journal of Evolutionary Biology **28**, 481–492 (2015).

Bossuyt, F., Brown, R. M., Hillis, D. M., Cannatella, D. C. & Milinkovitch, M. C. Phylogeny and biogeography of a cosmopolitan frog radiation: Late Cretaceous diversification resulted in continent-scale endemism in the family Ranidae. Systematic Biology **55**, 579–594 (2006).

Brady, S. G., Schultz, T. R., Fisher, B. L. & Ward, P. S. Evaluating alternative hypotheses for the early evolution and diversification of ants. Proceedings of the National Academy of Science **103**, 18172–18177 (2006).

Bukontaite, R., Ranarilalatiana, T., Randriamihaja, J. H. & Bergsten, J. In or Out-of-Madagascar?—Colonization patterns for large-bodied diving beetles (Coleoptera: Dytisicdae). PLoS ONE **10**, e0120777 (2015).

Caccone, A., Amato, G., Gratry, O. C., Behler, J. & Powell, J. R. A molecular phylogeny of four endangered Madagascar tortoises based on mtDNA sequences. Molecular Phylogenetics and Evolution **12**, 1–9 (1999).

Chatterjee, H., Ho, S., Barnes, I. & Groves, C. Estimating the phylogeny and divergence times of primates using a supermatrix approach. BMC Evolutionary Biology **9**, 259 (2009).

Chenoweth, L. B. & Schwarz, M. P. Biogeographical origins and diversification of the exoneurine allodapine bees of Australia (Hymenoptera, Apidae). Journal of Biogeography **38**, 1471–1483 (2011).

Clark, R. & Vogler, A. P. A phylogenetic framework for wing pattern evolution in the mimetic Mocker Swallowtail Papilio dardanus. Molecular Ecology **18**, 3872–3884 (2009).

Crottini, A. et al. Vertebrate time-tree elucidates the biogeographic pattern of a major biotic change around the K –T boundary in Madagascar. Proceedings of the National Academy of Science **109**, 5358–5363 (2012).

Daniels, S. R., Cumberlidge, N., Pérez-Losada, M., Marijnissen, S. A. & Crandall, K. A. Evolution of Afrotropical freshwater crab lineages obscured by morphological convergence. Molecular Phylogenetics and Evolution **40**, 227–235 (2006).

Douady, C. J., Catzeflis, F., Springer, M. S. & Stanhope, M. J. Molecular evidence for the monophyly of Tenrecidae (Mammalia) and the timing of the colonization of Madagascar by Malagasy tenrecs. Molecular Phylogenetics and Evolution **22**, 357–363 (2002).

Douady, C. J. & Douzery, E. J. P. Molecular estimation of eulipotyphlan divergence times and the evolution of "Insectivora". Molecular Phylogenetics and Evolution **28**, 285–296 (2003).
Federman, S. et al. The biogeographic origin of a radiation of trees in Madagascar: implications for the assembly of a tropical forest biome. BMC Evolutionary Biology **15**, 216 (2015).
Forthman, M. & Weirauch, C. Phylogenetics and biogeography of the endemic Madagascan millipede assassin bugs (Hemiptera: Reduviidae: Ectrichodiinae). Molecular Phylogenetics and Evolution **100**, 219–233 (2016).
Fuchs, J., Fjeldså, J. & Pasquet, E. An ancient African radiation of corvoid birds (Aves: Passeriformes) detected by mitochondrial and nuclear sequence data. Zoologica Scripta **35**, 375–385 (2006).
Giribet, G. & Edgecombe, G. D. Stable phylogenetic patterns in scutigeromorph centipedes (Myriapoda: Chilopoda: Scutigeromorpha): dating the diversification of an ancient lineage of terrestrial arthropods. Invertebrate Systematics **27**, 485–501 (2013).
Gunter, N. L., Weir, T. A., Slipinski, A., Bocak, L. & Cameron, S. L. If dung beetles (Scarabaeidae: Scarabaeinae) arose in association with dinosaurs, did they also suffer a mass coextinction
at the K-Pg Boundary. PLoS ONE **11**, e0153570 (2016).
Herrera, J. P. & Dávalos, L. M. Phylogeny and divergence times of lemurs inferred with recent and ancient fossils in the tree. Systematic Biology **65**, 772–791 (2016).
Horvath, J. E. et al. Development and application of a phylogenomic toolkit: resolving the evolutionary history of Madagascar's lemurs. Genome Research **18**, 489–499 (2008).
Janssen, T. et al. Neoendemism in Madagascan scaly tree ferns results from recent, coincident diversification bursts Evolution **62**, 1876–1889 (2008).
Jønsson, K. A. et al. Ecological and evolutionary determinants for the adaptive radiation of the Madagascan vangs. Proceedings of the National Academy of Science **109**, 6620–6625 (2012).
Kistler, L. et al. Comparative and population mitogenomic analyses of Madagascar's extinct, giant 'subfossil' lemurs. Journal of Human Evolution **79**, 45–54 (2015).
Kodandaramaiah, U. et al. Phylogenetics and biogeography of a spectacular Old World radiation of butterflies: the subtribe Mycalesina (Lepidoptera: Nymphalidae: Satyrini). BMC Evolutionary Biology **10**, 172 (2010).
Köhler, F. & Glaubrecht, M. Uncovering an overlooked radiation: molecular phylogeny and biogeography of Madagascar's endemic river snails (Caenogastropoda: Pachychilidae: Madagasikara gen. nov.). Biological Journal of the Linnean Society **99**, 867–894 (2010).
Koopman, M. M. & Baum, D. A. Phylogeny and biogeography of tribe Hibisceae (Malvaceae) on Madagascar. Systematic Botany **32**, 364–374 (2008).
Kuntner, M. & Agnarsson, I. Biogeography and diversification of hermit spiders on Indian Ocean islands (Nephilidae: Nephilengys). Molecular Phylogenetics and Evolution **59**, 477–488 (2011).
Kuntner, M. & Agnarsson, I. Phylogeography of a successful aerial disperser: the golden orb spider Nephila on Indian Ocean islands. BMC Evolutionary Biology **11**, 119 (2011).
Magallón, S., Gómez-Acevedo, S., Sánchez-Reyes, L. L. & Hernández-Hernández, T. A metacalibrated time-tree documents the early rise of flowering plant phylogenetic diversity. New Phytologist **20**7, 437–453 (2015).
Matschiner, M. et al. Bayesian phylogenetic estimation of clade ages support trans-Atlantic dispersal of cichlid fishes. Systematic Biology **0**, 1–20, doi:10.1093/sysbio/syw076 (2016).
Mitchell, K. J. et al. Ancient DNA reveals elephant birds and kiwi are sister taxa and clarifies

ratite bird evolution. Science **344**, 898-900 (2016).
Müller, S. et al. Intercontinental long-distance dispersal of Canellaceae from the New to the Old World revealed by a nuclear single copy gene and chloroplast loci. Molecular Phylogenetics and Evolution **84**, 205–219 (2015).
Nagy, Z. T., Joger, U., Wink, M., Glaw, F. & Vences, M. Multiple colonization of Madagascar and Socotra by colubrid snakes: evidence from nuclear and mitochondrial gene phylogenies. Proceedings of the Royal Society B **270**, 2613–2621 (2003).
Nobre, T., Eggleton, P. & Aanen, D. K. Vertical transmission as the key to the colonization of Madagascar by fungus-growing termites? Proceedings of the Royal Society B **277**, 359–365 (2010).
Noonan, B. P. & Chippindale, P. T. Dispersal and vicariance: the complex evolutionary history of boid snakes. Molecular Phylogenetics and Evolution **40**, 347–358 (2006).
Noonan, B. P. & Chippindale, P. T. Vicariant origin of Malagasy reptiles supports Late Cretaceous Antarctic land bridge. The American Naturalist **168**, 730–741 (2006).
Okajima, Y. & Kumazawa, Y. Mitogenomic perspectives into iguanid phylogeny and biogeography: Gondwanan vicariance for the origin of Madagascan oplurines. Gene **441**, 28–35 (2009).
Palkovacs, E. P., Gerlach, J. & Caccone, A. The evolutionary origin of Indian Ocean tortoises (Dipsochelys). Molecular Phylogenetics and Evolution **24**, 216–227 (2002).
Perelman, P. et al. A molecular phylogeny of living primates. PLoS Genetics **7**, e1001342 (2011).
Poux, C., Madsen, O., Glos, J., de Jong, W. W. & Vences, M. Molecular phylogeny and divergence times of Malagasy tenrecs: Influence of data partitioning and taxon sampling on dating analyses. BMC Evolutionary Biology **8**, 102 (2008).
Poux, C. et al. Asynchronous colonization of Madagascar by the four endemic clades of primates, tenrecs, carnivores, and rodents as inferred from nuclear genes. Systematic Biology **54**, 719–730 (2005).
Prum, R. O. et al. A comprehensive phylogeny of birds (Aves) using targeted next-generation DNA sequencing. Nature **526**, 569–573 (2015).
Raselimanana, A. P., Noonan, B. P., Karanth, K. P., Gauthier, J. & Yoder, A. D. Phylogeny and evolution of Malagasy plated lizards. Molecular Phylogenetics and Evolution **50**, 336–344 (2009).
Raxworthy, C. J. et al. Continental speciation in the tropics: contrasting biogeographic patterns of divergence in the Uroplatus leaf-tailed gecko radiation of Madagascar. Journal of Zoology **275**, 423–440 (2008).
Reddy, S., Driskell, A., Rabosky, D. L., Hackett, S. J. & Schulenberg, T. S. Diversification and the adaptive radiation of the vangas of Madagascar. Proceedings of the Royal Society B **279**, 2062–2071 (2012).
Rehan, S. M. et al. Molecular phylogeny of the small carpenter bees (Hymenoptera: Apidae: Ceratinini) indicates early and rapid global diserpsal. Molecular Phylogenetics and Evolution **55**, 1042–1054 (2010).
Renner, S. S., Strijk, J. S., Strasberg, D. & Thébaud, C. Biogeography of the Monimiaceae (Laurales): a role for East Gondwana and long-distance dispersal, but no West Gondwana. Journal of Biogeography **37**, 1127–1238 (2010).
Schaefer, H., Heibl, C. & Renner, S. S. Gourds afloat: a dated phylogeny reveals an Asian origin of the gourd family (Cucurbitaceae) and numerous oversea dispersal events. Proceedings of the

Royal Society B **276**, 843–851 (2009).
Sole, C. L., Wirta, H., Forgie, S. A. & Scholtz, C. H. Origin of Madagascan Scarabaeini dung beetles (Coleopetera: Scarabaeidae): dispersal from Africa. Insect Systematics & Evolution **42**, 29–40 (2011).
Springer, M. S. et al. Macroevolutionary dynamics and historical biogeography of primate diversification inferred from a species supermatrix. PLoS ONE **7**, e49521 (2012).
Strijk, J. S. et al. In and out of Madagascar: Dispersal to peripheral islands, insular speciation and diversification of Indian Ocean daisy trees (Psiadia, Asteraceae). PLoS ONE **7**, e42932 (2012).
Tank, D. C. et al. Nested radiations and the pulse of angiosperm diversification: increased diversification rates often follow whole genome duplications. New Phytologist **207**, 454–467 (2015).
Teeling, E. C. et al. A molecular phylogeny for bats illuminates biogeography and the fossil record. Science **307**, 580–584 (2005).
Tolley, K. A., Townsend, T. M. & Vences, M. Large-scale phylogeny of chameleons suggests African origins and Eocene diversification. Proceedings of the Royal Society B **280**, 20130184 (2013).
Toon, A. et al. Gondwanan radiation of the Southern Hemisphere crayfishes (Decapoda: Parastacidae): evidence from fossils and molecules. Journal of Biogeography **37**, 2275–2290 (2010).
Toussaint, E. F. A., Fikáček, M. & Short, A. E. Z. India–Madagascar vicariance explains cascade beetle biogeography. Biological Journal of the Linnean Society **118**, 982–991 (2016).
Townsend, T. M. et al. Phylogeny of iguanian lizards inferred from 29 nuclear loci, and a comparison of concatenated and species-tree approaches for an ancient, rapid radiation. Molecular Phylogenetics and Evolution **61**, 363–380 (2011).
Van Bocxlaer, I., Roelants, K., Biju, S. D., Nagaraju, J. & Bossuyt, F. Late Cretaceous vicariance in Gondwanan amphibians. PLoS ONE **1**, e74 (2006).
van der Meijden, A. et al. Nuclear gene phylogeny of narrow-mouthed toads (Family: Microhylidae) and a discussion of competing hypotheses concerning their biogeographical origins. Molecular Phylogenetics and Evolution **44**, 1017–1030 (2007).
van der Meijden, A., Vences, M., Hoegg, S. & Meyer, A. A previously unrecognized radiation of ranid frogs in Southern Africa revealed by nuclear and mitochondrial DNA sequences. Molecular Phylogenetics and Evolution **37**, 674–685 (2005).
Vargas-Ramírez, M., Castaño-Mora, O. V. & Fritz, U. Molecular phylogeny and divergence times of ancient South American and Malagasy river turtles (Testudines: Pleurodira: Podocnemididae). Organisms, Diversity & Evolution **8**, 388–398 (2008).
Vences, M., Freyhof, J., Sonnenberg, R., Kosuch, J. & Veith, M. Reconciling fossils and molecules: Cenozoic divergence of cichlid fishes and the biogeography of Madagascar. Journal of Biogeography **28**, 1091–1099 (2001).
Vences, M. et al. Multiple overseas dispersal in amphibians. Proceedings of the Royal Society B **270**, 2435–2442 (2003).
Verneau, O. et al. The double odyssey of Madagascan polystome flatworms leads to new insights on the origins of their amphibian hosts. Proceedings of the Royal Society B **276**, 1575–1583 (2009).
Vidal, N. et al. Blindsnake evolutionary tree reveals long history on Gondwana. Biology Letters **6**, 558–561 (2010).

Voelker, G. Systematics and historical biogeography of wagtails: dispersal versus vicariance revisited. The Condor **10**, 725–739 (2002).
Warren, B. H., Bermingham, E., Bowie, R. C., Prys-Jones, R. P. & Thébaud, C. Molecular phylogeography reveals island colonization history and diversification of western Indian Ocean sunbirds (Nectarinia: Nectariniidae). Molecular Phylogenetics and Evolution **29**, 67–85 (2003).
Warren, B. H., Bermingham, E., Prys-Jones, R. P. & Thébaud, C. Tracking island colonization history and phenotypic shifts in Indian Ocean bulbuls (Hypsipetes: Pycnonotidae). Biological Journal of the Linnean Society **85**, 271–287 (2005).
Wikström, N., Savolainen, V. & Chase, M. W. Evolution of the angiosperms: calibrating the family tree. Proceedings of the Royal Society B **268**, 2211–2220 (2001).
Wirta, H., Orsini, L. & Hanski, I. An old adaptive radiation of forest dung beetles in Madagascar. Molecular Phylogenetics and Evolution **47**, 1076–1089 (2008).
Wirta, H., Viljanen, H., Orsini, L., Montreuil, O. & Hanski, I. Three parallel radiations of Canthonini dung beetles in Madagascar. Molecular Phylogenetics and Evolution **57**, 710–727 (2010).
Wood, H. M., Gillespie, R. G., Griswold, C. E. & Wainwright, P. C. Why is Madagascar special? The extraordinarily slow evolution of pelican spiders (Araneae, Archaeidae). Evolution **69**, 462–481 (2015).
Wright, T. F. et al. A multilocus molecular phylogeny of the parrots (Psittaciformes): support for a Gondwanan origin during the Cretaceous. Molecular Biology and Evolution **25**, 2141–2156 (2008).
Yoder, A. D. et al. Single origin of Malagasy Carnivora from an African ancestor Nature **421**, 734–737 (2003).
Yoder, A. D. & Yang, Z. Divergence dates for Malagasy lemurs estimated from multiple gene loci: geological and evolutionary context. Molecular Ecology **13**, 757–773 (2004).
Zanne, A. E. et al. Three keys to the radiation of angiosperms into freezing environments Nature **506**, 89–92 (2014).

Table S3: Fossil taxa included in analysis.

group	Genus	species	Age Ma	Locality of deposit	Depository
Out-	*Triaplus*	*laticoxa*	221 - 235	Madygen area, Batken Region,Osh Oblast, Kyrgyzstan	PIN
Out-	*Haliplus*	*cretaceus*	112 - 125	Bon-Tsagan, outcrop 87.8, Bayankhongor Province, Mongolia	PIN
Out-	*Liadytes*	*longus*	125 - 150	Daya settlement, Glushkovo Formation, Russian Federation	PIN
Out-	*Mesodytes*	*rhantoides*	122 - 125	Yixian Formation, Liutiaogou, China	NIG
In-	*Angarogyrus*	*mongolicus*	112 - 125	Gurvan-Eren Formation, Govi-Altai, Mongolia	PIN
In-	*Angarogyrus*	*minimus*	175 - 183	Cheremkhovskaya Formation, Irkutsk, Russian Federation	PIN
In-	*Baissogyrus*	*savilovi*	112 - 125	Zaza Formation, Buryatia, Russian Federation	PIN
In-	*Mesogyrus*	*antiquus*	155 - 164	Karabastau Formation, Karatau-Mikhailovka, Kazakhstan	PIN
In-	*Mesogyrus*	*striatus*	122 - 150	Turga Formation, Undurga River, Russian Federation	PIN
In-	*Cretotortor*	*sp*	183-174	Liège deposit No IB 974, Bascharge, Luxemborg	MNHN
In-	*Cretotortor*	*zherichini*	89 - 94	Kzyl-Zhar deposits, Northeastern Karatau Range, Kazakhstan	PIN
In-	*Mesodineutes*	*amurensis*	61 - 66	Arkhara site, Darmakan Formation, Russian Federation	PIN
In-	*Gyretes*	*giganteus*	55 - 58	Menat, Pay-deDome, France	MNHN
In-	*Miodineutes*	*insignis*	11.0 - 12.0	Öhningen, Switzerland	ESSI

PIN: Borissiak Paleontological Institute, Russian Academy of Sciences, Moscow, Russia
MNHN: Muséum National d'Histoire Naturelle, Paris France
NIG: Nanjing Institute of Geology and Palaeontology, Chinese Academy of Sciences, Nanjing, China
ESSI: Erdwissenschaftliche Sammlung, Swiss Federal Institute of Technology, Zurich, Switzerland

Table S4: Additional taxa added to dataset.

Genus	species	voucher #	Collection data	COI	COII	12S	H3
Haliplus	*lineato-collis*	Hali	GenBank	AY071803	-	AY45647	AY745682
Macrogyrus	*howittii*	Ayhw 887	AUSTRALIA: Tasmania. Franklin Beach, Lake St. Clair. 10.i.2015 CHS Watts. MSBA	KX775606	-	KX775498	KX775705
Macrogyrus	*reichei*	Ayre 912	AUSTRALIA: VIC. Glenelg River nr. Dergholm, -37.36686, 141.2428. 75 m. 13.i.2015. Leg G.Gustafson. MSBA	KX775602	KX775654	KX775494	-
Macrogyrus	*striolatus*	Ayst 882	AUSTRALIA: NSW. Megalong Valley, -36.65629, 150.27377, 861 m. 04.i.2015. leg. G.Gustafson Forested strm GTG01042015A. MSBA	KX775607	KX775658	KX775499	KX775706
Andogyrus	*seria-topunc-tatus*	Adsr 886	Argentina. MSBA	KX775579	KX775629	KX775469	KX775679
Andogyrus	*colom-bicus*	Adco 828	VENEZUELA: Merida State 8°38.006'N, 71°09.762'W, 2037 m Monte Zerpa area; 20.vii.2009 leg. Short, Sites, Gustafson, Camacho; stream margin/pools VZ09-0720-01A/L-1098 ABTC-01476. MSBA	KX775578	KX775628	KX775468	KX775678
Macrogyrus	*toxopeusi*	AyC s829	INDONESIA: Papua: Poga 3°48.382'S, 138°34.780'E 2285-2330 m. ZSM	KX775605	KX775657	KX775497	KX775704
Macrogyrus	*purpur-ascens*	AyC s841	PAPUA NEW GUINEA: Morobe Prov. Pindiu. 6°27.147'S 147°29.574'E, 1470 m. 12.x.2009 leg. Inaho (PNG206). ZSM	KX775603	KX775655	KX775495	KX775702
Macrogyrus	*sumbawae*	Aysp 860	INDONESIA: Sumba: dry forest stream in limestone. 370 m 9°49.474'S 120°20.856'E (SUA08). ZSM	KX775617	KX775668	KX775509	KX775716
Macrogyrus	*sp. nr. blanchardii*	AyTs 834	PAPUA NEW GUINEA: E Highlands Prov. Onerunka, small creek, redsoil rock 6°20.936'S 145°46.874'E 1700 m. 21.v.2006 leg. John & Balke (PNG71). ZSM	KX775616	KX775667	KX775508	KX775715
Macrogyrus	*sp.*	AyTs 833	PAPUA NEW GUINEA: Central Prov. Woitape. 08°31.290'S 147°13.684'E 1700 m. i.2008 leg. Posman (PNG166). ZSM	KX775614	KX775665	KX775506	KX775713
Macrogyrus	*sp.*	AyC s831	PAPUA NEW GUINEA: Sandaun Prov. Mianmin 4°54.570'S 141°35.490'E 990 m. 23.x.2008. leg. Ibalim (PNG193). ZSM	KX775604	KX775656	KX775496	KX775703
Dineutus	*fulgidus*	DiDf 915	INDONESIA: Sumatra Barat, Solok, Alahan Panjank Road. 1190 m. 0°56.345'S 100°46.411'E. ZSM	KX775593	KX775643	KX775483	KX775692
Dineutus	*n. sp.*	DiDn 865	BALI: Telaga Forest, BLI07. ZSM	KX775594	KX775644	KX775484	KX775693
Dineutus	*tetra-canthus*	DiRt 908	PAPUA NEW GUINEA: Madang Prov. Wannang, 5°15.458'S 145°2.389'E 270 m. 31.x.2008. leg. Posman (PNG187) ZSM.	KX775598	KX775650	KX775490	KX775698
Dineutus	*longimanus longimanus*	Dilo 818	DOMINICAN REP.: Pedernales Prov. W of Pedernales on rd. to border with Haiti; 15 May 2010 18.154° -71.7582° colr. G. J. Svenson. MSBA	KX775585	KX775636	KX775475	KX775686

Dineutus	*pagdeni*	DiCp 918	SOLOMON ISLANDS: Guadal-canal ca. 4.5 km S of Barana vill. Forest nr. "Japanese Camp" & Moka riv. 9°30.3'S 159°58.9'E, 27m. 5-6.xii.2013 leg. Jiří Hájek. MSBA	KX775580	KX775631	KX775471	KX775681
Dineutus	*carolinus*	Dica 821	USA: TX: Hardin Co. 30.260° -94.525° 20m 28.vi.2013 colr. CK Faris & GT Gustafson. Mud bottomed bayou GTG06281301. MSBA	KX775583	KX775634	KX775473	KX775684
Dineutus	*assimilis*	Dias 819	USA: KS: Osage Co. pond off hwy 69 nr milemark 54. 38.031° -94.705° 254m 17.vi.2013 leg. CK Faris & GT Gustafson GTG06171301. MSBA	KX775582	KX775633	-	KX775683
Dineutus	*robertsi*	Diro 913	USA: Georgia: Warwoman Wld Mgmt Area. Tuckaluge Cr. 34.90155°N 83.30015°W. 533 m. 11July2012. KB Miller colr. KBM11071201. MSBA	KX775588	KX775639	KX775478	-
Porro-rhynchus	*landaisi*	Prla8 52	CHINA: Hainan Isl. Jianfengling Mts. Tiachi Lake rd. from Taichi village to 'Sector 5' 18°43.6-44.1'N 820-950m, 108°52.1-52.5'E 10.v.2011. M.Fikáček & Sh. Zhao lgt. Small slow-flowing stony river in the primary forest. MSBA	KX775618	KX775669	KX775510	KX775717
Enhydrus	sulcatus	Ehsu 856	BRAZIL: Rio de Janeiro: Cachoei Ras de Macacu - Regua. 01.vi.2013. Ponto do Ganesh (Parte Alta) col. Equipe Coleoptera. MSBA	KX775599	KX775651	KX775491	KX775699
Dineutus	*fairmairei*	DiCf 916	Fiji03. ZSM	-	KX775630	KX775470	KX775680
Dineutus	*australis*	Diau 911	AUSTRALIA: QLD. 19°41.153'S 145°49.536'E 14.Mar.2011. colr. KB Miller KBM14031102. MSBA	KX775592	KX775642	KX775482	-
Dineutus	*macro-chirus*	DiM m919	PAPUA NEW GUINEA: Central Prov. Kokoda Trek. 9°00.338'S 147°44.252'E. 1390 m. i.2008. leg. Posman (PNG 173). ZSM	-	KX775645	KX775485	-
Dineutus	*priscus*	DiMp 917	PAPUA NEW GUINEA: S Highlands Prov. Sopulkul, 30-35 km NE Mendi. 6°2.944'S 143°46.485'E. 2679 m. 16.vi.2006. Leg. John ex swamp into stream (PNG 79). ZSM	-	KX775646	KX775486	KX775694
Orecto-gyrus	*ornaticollis*	Ogor 901	MADAGASCAR: Analamanga Reg. Ankazobe dist. Ambohitantely Res. -18.1808°, 47.2901°, 1340 m. 22.xi.2014 leg. G. Gustafson. MAD14-76. MSBA	KX775625	-	KX775516	-
Orecto-gyrus	*heros*	Oghe 900	MADAGASCAR: Analamanga Reg. Ankazobe dist. Ambohitantely Res. -18.1808°, 47.2901°, 1340 m. 22.xi.2014 leg. G. Gustafson. MAD14-76. MSBA	KX775623	KX775674	KX775515	-
Patrus	*sp.*	Pasp 897	THAILAND: Kanchanaburi Prov. Huey Ka Yaeng at Ban Pracham Mai 16°21.0277'N, 101°19.541'E 428 m. 19.i.2015. gravel streams leg G.Gustafson. MSBA	KX775518	KX775626	KX775676	KX775720
Patrus	*sp.*	Pasp 896	THAILAND: Kanchanaburi Prov. Heuy Ou Long, opposite Keong Ka Wia Reforestation Station 14°33.199'N, 98°34.238'E 326 m. 19.i.2015. gravel streams leg G.Gustafson. MSBA	KX775517	KX775625	KX775675	-

Patrus	*sp.*	Pasp 898	THAILAND: Kanchanaburi Prov. Huey Ka Yaeng at Ban Pracham Mai 16°21.0277'N, 101°19.541'E 428 m. 19.i.2015. gravel streams leg G.Gustafson. MSBA	KX775519	KX775627	KX775677	KX775721
Gyrinus	*sericeo-limbatus*	Gysp 840	PAPUA NEW GUINEA: Morobe Prov. Pindiu. 6°27.147'S 147°29.574'E, 1470 m. 12.x.2009 leg. Inaho (PNG206). ZSM	KX775620	KX775671	KX775512	KX775718
Gyrinus	*maculi-ventris*	Gysp 837	USA: New Mexico, Cibola Co., Zuni Mountains. 18.v.2013. leg. S. Baca. 180513-A. MSBA	KX775621	KX775672	KX775513	-
Gyrinus	*dimorphus*	Gysp 839	USA: New Mexico, Cibola Co., Zuni Mtns, 35.40178°N, 108.44956°W. 30.v.2013. leg. G.Gustafson & S.Baca. GGSB053013C. MSBA	KX775622	KX775673	KX775514	KX775719

Depository abbreviations:
MSBA: Museum of Southwestern Biology, University of New Mexico, Albuquerque, NM, USA.
ZSM: Zoologische Staatssammlung München, Munich, Germany

Table S5: The effect of FBD parameters on divergence times estimates.

	Preferred	spec exp 1	spec exp 100
Net diversification prior:	exp(10)	exp(1)	exp(100)
Sampling proportion:	0.1	0.1	0.1
Piecewise FBD breakpoints:	1 timeslice	1 timeslice	1 timeslice
root	266 (255-272)	267 (256-272)	267 (256-272)
OG excl. Triaplus crown	236 (209-258)	237 (214-259)	236 (211-261)
Gyrinidae stem	255 (236-271)	256 (240-270)	256 (237-271)
**Gyrinidae crown**	**235 (214-255)**	**236 (214-253)**	**235 (213-256)**
Spanglerogyrinae crown	184 (174-202)	185 (174-202)	185 (174-202)
Heterogyrinae crown	183 (174-199)	184 (174-200)	183 (174-199)
**Heterogyrinae-Gyrininae**	**206 (187-226)**	**208 (189-227)**	**208 (188-226)**
Gyrininae crown	175 (152-198)	175 (153-198)	174 (152-194)
Gyrinini crown	98 (77-118)	97 (79-118)	97 (80-118)
Dineutini-Orectochilini	160 (139-182)	160 (138-180)	158 (139-178)
Dineutini crown	137 (118-158)	138 (117-158)	136 (118-156)
Orectochilini crown	136 (115-155)	138 (120-157)	134 (115-152)

Table S5 continued.

	Piecewise 2	Piecewise 3	Cryptic div.
Net diversification prior:	exp(10)	exp(10)	exp(10)
Sampling proportion:	0.1	0.1	0.01
Piecewise FBD breakpoints:	201 my	201 and 66 my	1 timeslice
root	265 (255-272)	263 (254-272)	261 (252-271)
OG excl. Triaplus crown	239 (214-262)	229 (194-255)	219 (191-245)
Gyrinidae stem	257 (241-271)	249 (226-268)	239 (218-261)
**Gyrinidae crown**	**238 (219-257)**	**229 (209-251)**	**225 (202-255)**
Spanglerogyrinae crown	184 (174-198)	180 (174-195)	185 (174-200)
Heterogyrinae crown	182 (174-198)	179 (174-191)	182 (174-194)
**Heterogyrinae-Gyrininae**	**203 (186-231)**	**198 (181-220)**	**198 (181-220)**
Gyrininae crown	173 (153-195)	166 (144-187)	158 (137-182)
Gyrinini crown	97 (79-117)	91 (70-117)	87 (68-105)
Dineutini-Orectochilini	158 (37-179)	152 (133-172)	142 (122-161)
Dineutini crown	137 (116-156)	131 (111-151)	121 (102-140)
Orectochilini crown	135 (115-153)	128 (108-149)	117 (101-136)

Table S6: The effect of relaxed clock models, clock base rate and among lineage rate variation on divergence times estimates.

	Preferred	wide cl.rate	igvar1	igvar100	TN02
relaxed clock	IGR	IGR	IGR	IGR	TN02
IGR var prior:	exp(10)	exp(10)	exp(1)	exp(100)	exp(10)
clock base rate prior:	logn.(-5.7,0.3)	logn.(-5.7,0.6)	logn.(-5.7,0.3)	logn.(-5.7,0.3)	logn.(-5.7,0.3)
root	266 (255-272)	266 (255-272)	267 (256-272)	267 (255-272)	266 (255-272)
OG excl. Triaplus crown	236 (209-258)	235 (210-257)	237 (212-261)	237 (212-259)	241 (220-261)
Gyrinidae stem	255 (236-271)	255 (237-270)	257 (238-272)	257(239-271)	257 (243-271)
**Gyrinidae crown**	**235 (214-255)**	**235 (214-254)**	**237 (216-255)**	**236 (216-256)**	**238 (221-254)**
Spanglerogyrinae crown	184 (174-202)	184 (174-202)	184 (174-202)	185 (174-203)	181 (174-193)
Heterogyrinae crown	183 (174-199)	183 (174-199)	184 (174-201)	184 (174-201)	181 (174-195)
**Heterogyrinae-Gyrininae**	**206 (187-226)**	**206 (187-225)**	**208 (188-229)**	**208 (188-229)**	**208 (192-224)**
Gyrininae crown	175 (152-198)	173 (152-195)	176 (153-198)	175 (153-198)	179 (162-196)
Gyrinini crown	98 (77-118)	96 (79-117)	99 (81-119)	98 (79-119)	134 (117-153)
Dineutini-Orectochilini	160 (139-182)	157 (139-177)	161 (141-183)	160 (139-182)	151 (135-168)
Dineutini crown	137 (118-158)	136 (116-157)	139 (118-158)	138 (118-159)	134 (118-152)
Orectochilini crown	136 (115-155)	134 (115-152)	136 (119-159)	136 (114-156)	107 (93-124)

Table S7: The effect of root age prior on divergence times estimates.

root prior	Preferred unif[252-273]	root unif wide unif[221-299]	root exp exp(off:252, m:272)	root exp wide exp(off:221, m:299)
root	266 (255-272)	286 (261-299)	278 (252-318)	303 (260-365)
OG excl. Triaplus crown	236 (209-258)	248 (218-279)	242 (212-280)	257 (220-300)
Gyrinidae stem	255 (236-271)	269 (244-295)	262 (237-300)	280 (244-322)
**Gyrinidae crown**	**235 (214-255)**	**245 (220-271)**	**241 (217-272)**	**254 (220-289)**
Spanglerogyrinae crown	184 (174-202)	186 (174-205)	185 (174-205)	187 (174-199)
Heterogyrinae crown	183 (174-199)	186 (174-205)	185 (174-203)	188 (174-210)
**Heterogyrinae-Gyrininae**	**206 (187-226)**	**215 (190-239)**	**211 (187-236)**	**220 (194-250)**
Gyrininae crown	175 (152-198)	180 (156-206)	177 (154-202)	186 (159-215)
Gyrinini crown	98 (77-118)	102 (82-122)	98 (79-120)	103 (84-126)
Dineutini-Orectochilini	160 (139-182)	164 (142-188)	161 (142-186)	170 (147-196)
Dineutini crown	137 (118-158)	141 (120-164)	139 (117-162)	147 (125-172)
Orectochilini crown	136 (115-155)	140 (120-162)	137 (116-161)	145 (123-168)

Table S8: The effect of sampling assumption on divergence times estimates.

	Preferred	Random sampl.	Fossiltips
fossils as ancestors	yes	yes	no
sampling extant taxa	diversified	random	random
root	266 (255-272)	269 (260-272)	269 (261-272)
OG excl. Triaplus crown	236 (209-258)	247 (227-264)	247 (227-265)
Gyrinidae stem	255 (236-271)	264 (251-272)	265 (253-272)
**Gyrinidae crown**	**235 (214-255)**	**244 (226-260)**	**247 (230-262)**
Spanglerogyrinae crown	184 (174-202)	180 (174-197)	184 (175-202)
Heterogyrinae crown	183 (174-199)	182 (174-201)	188 (176-208)
**Heterogyrinae-Gyrininae**	**206 (187-226)**	**217 (199-235)**	**220 (200-238)**
Gyrininae crown	175 (152-198)	194 (173-214)	194 (175-215)
Gyrinini crown	98 (77-118)	113 (96-133)	113 (94-134)
Dineutini-Orectochilini	160 (139-182)	181 (158-200)	182 (163-202)
Dineutini crown	137 (118-158)	158 (138-178)	159 (139-179)
Orectochilini crown	136 (115-155)	157 (139-175)	158 (140-178)

Table S9: The effect of treeprior on divergence times estimates.

Treeprior<br>sampling	Preferred<br>FBD<br>diversified	Random sampl.<br>FBD<br>random	IGR rel. clock<br>uniform<br>NA	TK02 rel clock.<br>uniform<br>NA
root	266 (255-272)	269 (260-272)	268 (257-272)	269 (261-272)
OG excl. Triaplus crown	236 (209-258)	247 (227-264)	256 (241-269)	249 (232-263)
Gyrinidae stem	255 (236-271)	264 (251-272)	266 (255-272)	268 (259-272)
**Gyrinidae crown**	**235 (214-255)**	**244 (226-260)**	**258 (246-268)**	**256 (245-265)**
Spanglerogyrinae crown	184 (174-202)	180 (174-197)	189 (175-215)	187 (175-209)
Heterogyrinae crown	183 (174-199)	182 (174-201)	206 (182-236)	195 (178-220)
**Heterogyrinae-Gyrininae**	**206 (187-226)**	**217 (199-235)**	**248 (231-263)**	**234 (221-247)**
Gyrininae crown	175 (152-198)	194 (173-214)	239 (219-257)	220 (205-232)
Gyrinini crown	98 (77-118)	113 (96-133)	157 (132-188)	176 (158-193)
Dineutini-Orectochilini	160 (139-182)	181 (158-200)	230 (207-251)	205 (190-219)
Dineutini crown	137 (118-158)	158 (138-178)	211 (185-235)	190 (175-205)
Orectochilini crown	136 (115-155)	157 (139-175)	208 (184-234)	167 (151-186)

Table S10: The effect of node dating versus TED.

	Preferred	node205	node221	node252
Fossils	included	excluded	excluded	excluded
root prior	unif[252-273]	unif[205-273]	unif[221-273]	unif[252-273]
dating	TED	node	node	node
root	266 (255-272)	235 (206-266)	241 (221-268)	261 (252-271)
OG excl. Triaplus crown	236 (209-258)	200 (158-245)	207 (165-250)	228 (187-262)
Gyrinidae stem	255 (236-271)	235 (206-266)	241 (221-268)	261 (252-271)
**Gyrinidae crown**	**235 (214-255)**	**213 (188-243)**	**213 (185-245)**	**217 (187-252)**
Spanglerogyrinae crown	184 (174-202)	NA	NA	NA
Heterogyrinae crown	183 (174-199)	NA	NA	NA
**Heterogyrinae-Gyrininae**	**206 (187-226)**	**182 (174-205)**	**184 (174-208)**	**185 (174-210)**
Gyrininae crown	175 (152-198)	162 (143-186)	163 (143-188)	167 (147-191)
Gyrinini crown	98 (77-118)	97 (76-118)	98 (77-121)	100 (79-124)
Dineutini-Orectochilini	160 (139-182)	150 (130-172)	151 (131-173)	154 (134-176)
Dineutini crown	136 (115-155)	128 (107-152)	130 (109-153)	134 (112-157)
Orectochilini crown	137 (118-158)	128 (107-149)	129 (109-152)	134 (114-155)
